# Integrative analysis of the embryonic origin of adult neural stem cells in forebrain subventricular zones

**DOI:** 10.1093/nsr/nwag516

**Published:** 2026-08-29

**Authors:** Huan Wu, Ke Tang, Ruimin Xu, Tao Li, Keke Liang, Xuanxuan Wang, Deyuan Huang, Yijia Yang, Yanxuan Yin, Jiayang Xie, Chong Li, Xiaoyu Liu, Qing-Ran Bai, Chenfei Wang, Qin Shen, Shaorong Gao

**Affiliations:** Shanghai Key Laboratory of Maternal and Fetal Medicine, Clinical and Translational Research Center of Shanghai First Maternity and Infant Hospital, School of Life Sciences and Technology, Tongji University; Frontier Science Center for Stem Cell Research, Tongji University, Shanghai 200092, China; Key Laboratory of Spine and Spinal Cord Injury Repair and Regeneration of Ministry of Education, Orthopedic Department of Tongji Hospital, School of Life Sciences and Technology, Tongji University; Frontier Science Center for Stem Cell Research, Tongji University, Shanghai 200092, China; Shanghai Key Laboratory of Maternal and Fetal Medicine, Clinical and Translational Research Center of Shanghai First Maternity and Infant Hospital, School of Life Sciences and Technology, Tongji University; Frontier Science Center for Stem Cell Research, Tongji University, Shanghai 200092, China; Department of Neurology, Shanghai Tongji Hospital, School of Medicine, Tongji University, Shanghai 200092, China; Department of Neurology, Shanghai Tongji Hospital, School of Medicine, Tongji University, Shanghai 200092, China; Shanghai Key Laboratory of Maternal and Fetal Medicine, Clinical and Translational Research Center of Shanghai First Maternity and Infant Hospital, School of Life Sciences and Technology, Tongji University; Frontier Science Center for Stem Cell Research, Tongji University, Shanghai 200092, China; Shanghai Key Laboratory of Maternal and Fetal Medicine, Clinical and Translational Research Center of Shanghai First Maternity and Infant Hospital, School of Life Sciences and Technology, Tongji University; Frontier Science Center for Stem Cell Research, Tongji University, Shanghai 200092, China; Shanghai Key Laboratory of Maternal and Fetal Medicine, Clinical and Translational Research Center of Shanghai First Maternity and Infant Hospital, School of Life Sciences and Technology, Tongji University; Frontier Science Center for Stem Cell Research, Tongji University, Shanghai 200092, China; Shanghai Key Laboratory of Maternal and Fetal Medicine, Clinical and Translational Research Center of Shanghai First Maternity and Infant Hospital, School of Life Sciences and Technology, Tongji University; Frontier Science Center for Stem Cell Research, Tongji University, Shanghai 200092, China; Shanghai Key Laboratory of Maternal and Fetal Medicine, Clinical and Translational Research Center of Shanghai First Maternity and Infant Hospital, School of Life Sciences and Technology, Tongji University; Frontier Science Center for Stem Cell Research, Tongji University, Shanghai 200092, China; Shanghai Key Laboratory of Maternal and Fetal Medicine, Clinical and Translational Research Center of Shanghai First Maternity and Infant Hospital, School of Life Sciences and Technology, Tongji University; Frontier Science Center for Stem Cell Research, Tongji University, Shanghai 200092, China; Shanghai Key Laboratory of Maternal and Fetal Medicine, Clinical and Translational Research Center of Shanghai First Maternity and Infant Hospital, School of Life Sciences and Technology, Tongji University; Frontier Science Center for Stem Cell Research, Tongji University, Shanghai 200092, China; Key Laboratory of Spine and Spinal Cord Injury Repair and Regeneration of Ministry of Education, Orthopedic Department of Tongji Hospital, School of Life Sciences and Technology, Tongji University; Frontier Science Center for Stem Cell Research, Tongji University, Shanghai 200092, China; Key Laboratory of Spine and Spinal Cord Injury Repair and Regeneration of Ministry of Education, Orthopedic Department of Tongji Hospital, School of Life Sciences and Technology, Tongji University; Frontier Science Center for Stem Cell Research, Tongji University, Shanghai 200092, China; Key Laboratory of Spine and Spinal Cord Injury Repair and Regeneration of Ministry of Education, Orthopedic Department of Tongji Hospital, School of Life Sciences and Technology, Tongji University; Frontier Science Center for Stem Cell Research, Tongji University, Shanghai 200092, China; Tongji University Brain and Spinal Cord Clinical Research Center, Shanghai 200092, China; Shanghai Key Laboratory of Maternal and Fetal Medicine, Clinical and Translational Research Center of Shanghai First Maternity and Infant Hospital, School of Life Sciences and Technology, Tongji University; Frontier Science Center for Stem Cell Research, Tongji University, Shanghai 200092, China

**Keywords:** Vcam1, embryonic origin, adult NSCs, quiescence, lineage tracing, scMulti-omics

## Abstract

In the mammalian brain, adult neural stem cells (NSCs) located in the ventricular–subventricular zone (V–SVZ) of lateral ventricles and dentate gyrus continuously generate new neurons and glia throughout life. The adult NSCs in V–SVZ are derived from ventricular radial glial cells (RGCs) which become quiescent during embryonic development. However, the origin and establishment of this adult NSC pool remain elusive, primarily due to the challenge of tracing the lineage from embryonic RGCs to their quiescent adult successors. Here, by leveraging Vcam1—a marker continuously expressed from embryonic RGCs to adult NSCs, we unravel the dynamic process and regulatory mechanism underlying the fate bifurcation that gives rise to quiescent seed cells. We demonstrate that Vcam1^+^ embryonic RGCs directly contribute to the adult NSC pool. A subpopulation of quiescent Vcam1^+^ RGCs emerges early and is maintained throughout development, while a parallel population of proliferative Vcam1^+^ progenitors actively replenishes this quiescent reservoir. Through the combinatory analysis of single-cell multiomics and clonal lineage tracing of Vcam1^+^ cells, we identify the TGF-β/Smad3 pathway as a critical regulator of quiescence. Our work thus provides a mechanistic dissection of how the foundational adult NSC pool is established through precisely regulated fate decisions during embryogenesis, offering new insights into the principles of lifelong neurogenesis.

## INTRODUCTION

The development of the mammalian brain is complicated and highly ordered. Neural stem cells (NSCs), with the potential to self-renew and produce neuronal and glial cell types [1], are the cellular foundation for constructing brain structures and functions. Previous studies have shown that NSCs exist widely in the embryonic developing brain, but become restricted to two main regions in the adult nervous system: the ventricular-subventricular zone (V–SVZ), along the lateral ventricular wall, and the subgranular zone (SGZ) of the dentate gyrus [2–4].

During forebrain development, embryonic NSCs first appear as neuroepithelial cells lining the ventricular surface, then transform into radial glial cells (RGCs), which give rise to neurons and glial cells [5,6]. At late embryonic stages, most RGCs differentiate and migrate away, while a distinct subpopulation of slowly dividing RGCs becomes the embryonic origin of the adult NSCs, transitioning to specialized B1 astrocytes in the V–SVZ [7–9]. These type B1 cells, known as postnatal/adult NSCs, continuously generate glial cells and new neurons that migrate over a long distance to the olfactory bulb (OB) throughout life. The existence of adult NSCs presents a therapeutic potential for regenerative medicine through activating endogenous NSCs in cases of brain injury and cognitive disorders [10–15].

A pioneering study using the lineage tracing mouse line *Hopx-CreER^T2^* to delineate the embryonic origin and establishment of NSCs in adult SGZ presented a ‘continuous’ model depicting the generation behavior of adult NSCs [16]. However, the embryonic origin and mechanisms responsible for lineage specification of adult NSCs in V–SVZ remain elusive. One important challenge in studying the embryonic origin of adult NSCs in V–SVZ is the absence of tools for specific identification of embryonic progenitors of B1 cells (pre-B1 cells) *in vivo*. Previous studies investigating the origin of adult V–SVZ NSCs relied on fate-mapping approaches focused on slowly dividing cells [8,9]. The development of novel methods continuously marking embryonic RGCs to adult B1 cells would facilitate dissecting the developmental process of adult NSCs from the embryonic stage.

Our previous studies have revealed that the vascular cell adhesion molecule 1 (Vcam1) is specifically expressed in RGCs in the developing forebrain [17] and on the apical process of type B1 cells in the adult V–SVZ [18]. Although most RGCs lose Vcam1 expression during mid-embryonic development, Vcam1 is sustainedly expressed in the slowly dividing pre-B1 cells as they undergo transition from late embryonic to the adult state [17]. Persistent Vcam1 expression is required for NSC maintenance from embryo to adult [17–20]. These observations suggest that Vcam1 may serve as a valuable tool for tracing the lineage development of embryonic origin of adult V–SVZ NSCs.

In this study, we generated two knock-in mouse lines—*Vcam1* reporter and *Vcam1-CreER^T2^*—to investigate the developmental behavior of Vcam1-expressing RGCs. Using the dual-fluorescence reporter system, we profiled dynamic distribution of Vcam1^+^ NSCs throughout embryonic neurogenesis to adult. Lineage tracing with *Vcam1-CreER^T2^* revealed that embryonic Vcam1^+^ RGCs contribute directly to the adult NSC pool in the V–SVZ. During embryonic neurogenesis, Vcam1^+^ RGC population is heterogeneous, comprising both quiescent and actively dividing subpopulations. The quiescent subpopulation, which emerges by E10.5 and persists into adulthood, represents a primary source of adult NSCs, while the proliferative subpopulation gives rise to neurons as well as continuous generates post-dividing Vcam1^+^ RGCs to expand constituents of the quiescent pool. Single-cell transcriptomic analysis and pseudotemporal trajectory construction indicated that Vcam1^+^ cells undergo fate trifurcation during embryonic neurogenesis, with one branch culminating in a quiescent state that is maintained into adulthood. Integrated analysis of scRNA-seq and scATAC-seq data identified a regulatory network underlying quiescence. Functional studies demonstrated that phosphorylated Smad3, a key effector of the TGF-β pathway, plays a critical role in directing NSCs toward a quiescent fate. Together, our work uses Vcam1 as a powerful genetic hook to dissect the lineage progression and fate specification of embryonic progenitors that contribute to the adult NSC reservoir.

## RESULTS

### Vcam1 continuously marks NSCs from embryo to adult

To investigate the expression pattern of Vcam1^+^ cells at different development stages in the forebrain, we generated the *Vcam1* reporter transgenic mouse line (Fig. 1A). Within the reporter line, Vcam1^+^ cells are able to be marked by enhanced green fluorescent protein (EGFP) expression. When crossed with a recombinase Cre transgenic mouse line, Cre-expressing cells would remove the EGFP coding sequence between loxP sites and switch Vcam1^+^ cells from green to red by tdTomato (tdT) expression (Fig. 1A).

**Figure 1.**
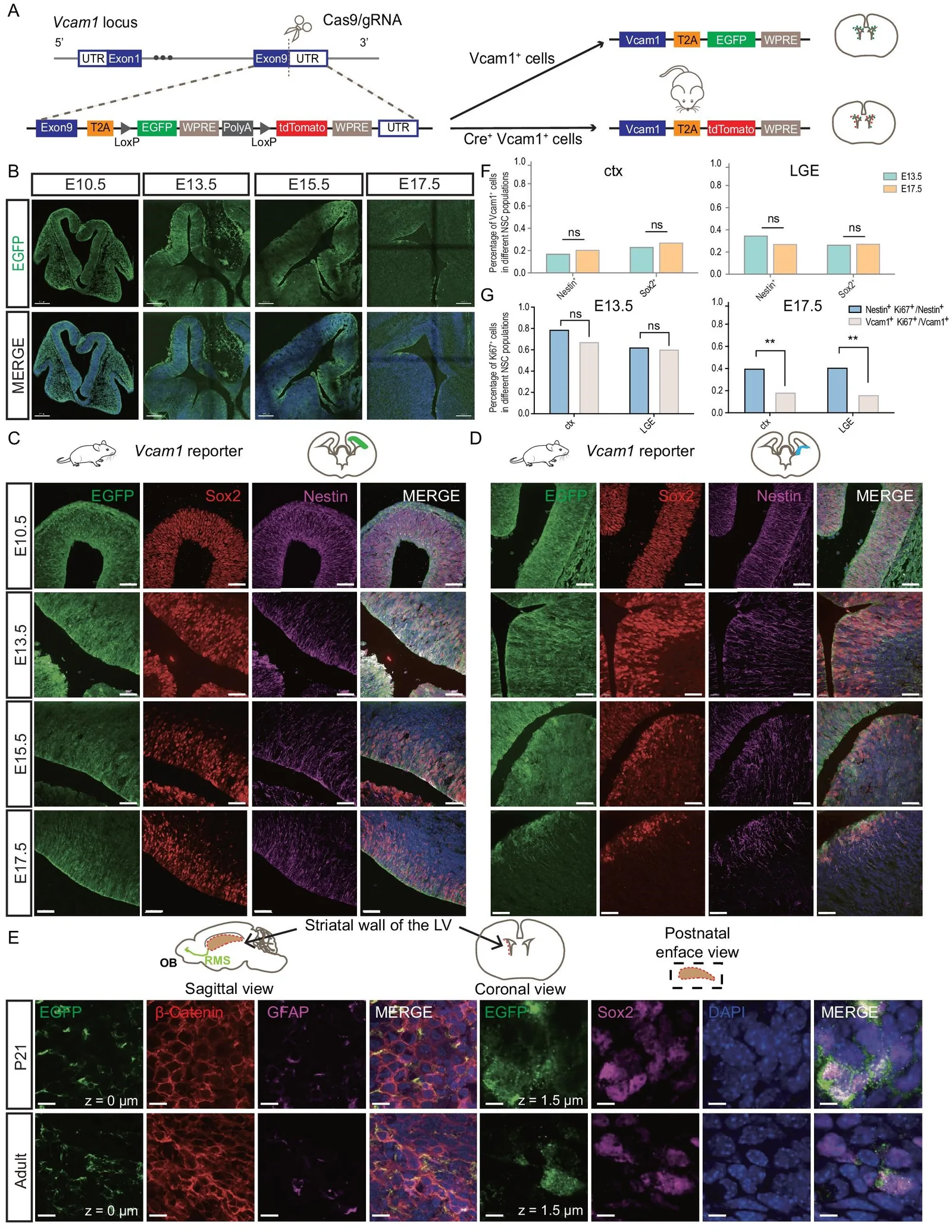
VCAM1 Continuously Marks a Subset of NSCs from Embryo to Adult. (A) Strategy for generating the *Vcam1* reporter allele. (B) Representative confocal images of immunofluorescent (IF) staining with EGFP at the indicated stages of the *Vcam1* reporter mouse in the cortex. *n* = 6–8 brains in each embryonic stage; scale bar, 150 μm. (C) Representative confocal images of IF staining for EGFP, Sox2 and Nestin at the indicated stages of the *Vcam1* reporter mouse in the cortex. *n* = 6–8 brains in each embryonic stage; scale bar, 40 μm. (D) Representative confocal images of IF staining of EGFP, Sox2 and Nestin at the indicated stages of the *Vcam1* reporter mouse in the LGE. *n* = 6–8 brains in each embryonic stage; scale bar, 40 μm. (E) Schematic diagram of the striatal wall of the LV in sagittal and coronal view (top). En face wholemount staining of the SVZ showing EGFP co-localized with β-catenin and GFAP, as well as EGFP co-stained with Sox2 and nuclear DAPI in V–SVZ wholemount preparations at P21 and adult stages. Confocal *z*-slices at *z* = 0 μm (sagittal) and *z* = 1.5 μm (en face wholemount) are displayed with individual channel and merged images. Scale bar, 3 μm. OB, olfactory bulb; RMS, rostral migratory stream. (F) Statistical analysis of Vcam1^+^ cells in other NSC populations in the cortex and LGE at E13.5 and E17.5. Data are presented as mean ± SEM. ns, not significant (two-way ANOVA). (G) Statistical analysis of Ki67^+^ cells in Vcam1^+^ and Sox2^+^ cells in the cortex and LGE at E13.5 and E17.5. Data were presented as mean ± SEM. Statistical significance was tested by one‐way ANOVA. **P < 0.01.

We first confirmed the cell-type labeling specificity of the *Vcam1* reporter mouse line. Immunostaining of embryonic brain sections showed that EGFP expression, consistent with endogenous Vcam1 protein, was restricted to the germinal zone of both the cortex (ctx) and lateral ganglionic eminence (LGE) from E13.5 to E17.5 (Fig. S1A and B). Vcam1 immunoreactivity was exclusively found in EGFP^+^ cells, though the subcellular localization differed due to the diffuse cytoplasmic distribution of EGFP (Fig. S1A, B). Postnatal whole-mount staining of the enface of the V–SVZ further validated the specificity, showing Vcam1 expression only in EGFP^+^ cells on the ventricular surface (Fig. S1C).

To further verify the reporter system, we crossed *Vcam1* reporter mice with either *Nestin-Cre* or *Emx1-Cre* lines. In *Nestin-Cre; Vcam1* reporter (N-VR) embryos at E14.5, tdT was present in the ventricular zone (VZ), confirming that a subset of Vcam1^+^ cells belong to the Nestin^+^ progenitor lineage (Fig. S1D). In *Emx1-Cre; Vcam1* reporter (E-VR) embryos, tdT was restricted to the dorsal cortex, while EGFP remained detectable in ventral regions, illustrating both lineage overlapping in cortex and regional specificity of the recombination in reporter line (Fig. S1E).

We next characterized the temporal expression pattern of Vcam1 using our reporter line by immunostaining brain sections from the onset of neurogenesis (E10.5), the earliest developmental timepoint analyzed, to adulthood (Fig. 1B). During embryonic stages, the EGFP signal intensity progressively enriched in the VZ of both the cortex and LGE as development proceeded. After birth, EGFP expression declined markedly in the neocortex and became restricted to the striatal side of the lateral ventricle. In the adult V–SVZ, EGFP^+^ cells were observed along the striatal ventricular wall and the dorsolateral edge of the V–SVZ, indicating specific localization within the neurogenic niche.

To confirm the identity of EGFP^+^ cells, we performed immunohistological and morphological analyses. During embryonic stages, most EGFP^+^ cells in the cortex and LGE exhibited radial glial morphology and expressed the RGC markers Nestin and Sox2 (Fig. 1C, D). We also performed whole-mount staining of the postnatal and adult V–SVZ. Imaging at both the surface (*z* = 0) and deeper (*z* = 1.5 µm) planes revealed that EGFP signal was specifically enriched in the center of pinwheel structures surrounded by ependymal cells—localizing to the apical endfeet of B1 cells (Fig. 1E). This pattern precisely matches the published Vcam1 staining in Kokovay *et al.* (Cell Stem Cell, 2012) [18]. Deeper imaging further confirmed that GFP^+^Vcam1^+^ cells expressed Sox2, identifying them as adult NSCs (Fig. 1E). These results provide direct evidence that Vcam1 continuously marks NSCs from embryo to adult.

It is important to note that the EGFP signal in our reporter line exhibits a diffuse cytoplasmic localization, distinct from the membrane-associated pattern observed with Vcam1 antibody staining. Moreover, RGCs are tightly packed side-by-side in the VZ, making individual cell boundaries unresolvable on coronal sections. This combination makes the EGFP signal appear relatively homogeneous in section and complicates precise quantification. To obtain accurate quantitative data while circumventing this tissue-density artifact, we performed acute staining of dissociated primary cells from the cortex and LGE at E13.5 and E17.5 (Fig. S2A; see also Hu, Chen *et al.*, 2017) [17]. Within Nestin⁺ or Sox2⁺ stem/progenitor populations, Vcam1 marked a relatively constant 20%–40% of cells (Fig. 1F), confirming that Vcam1 is indeed restricted to a subset of NSCs rather than a pan-progenitor marker.

To further investigate the relationship between Vcam1 expression and quiescence, we combined this analysis with Ki67 staining. At E13.5, the proportion of Ki67^+^ cells within the Vcam1^+^ population was comparable to that in the general Nestin^+^ population. However, by E17.5, a significant and consistent enrichment of Vcam1 expression was observed specifically within the Ki67^–^ (quiescent) population (Fig. 1G). These data demonstrate not only that Vcam1 marks a subset of NSCs, but also that it acquires a progressively stronger preference for quiescent cells during late embryonic development.

To independently validate this finding, we performed EdU pulse-chase labeling to identify slow-cycling, quiescent cells. EdU was administered intraperitoneally at E10.5 at 2-h intervals to maximize labeling efficiency, and brain tissues were collected at E17.5 (Fig. S2B). To enable precise quantification at the single-cell level, we microdissected the cortex and LGE from fresh E17.5 brains and performed acute dissociation followed by immunostaining on the primary cells (Fig. S2C). This approach revealed that Vcam1^+^ cells contained a significantly higher proportion of EdU label-retaining cells (LRCs) compared to those expressing Nestin in the cortex and LGE (Fig. S2D), indicating that Vcam1 exhibits higher specificity for labeling the quiescent NSC subpopulation during embryonic development than broader RGC marker, both in the cortex and LGE.

To further substantiate the reliability of our staining and quantification, we performed flow cytometry analysis (Fig. S2D). In the cortex, the proportion of LRCs in Vcam1^+^ cells was 9.7% and 10.45% (*n* = 2), compared to 4.23% and 4.37% in Nestin^+^ cells. In the LGE, Vcam1^+^ cells contained 7.69% and 15.79% LRCs (*n* = 2), whereas Nestin^+^ cells contained 2.40% and 2.45%. These flow cytometry results are consistent with our immunofluorescence data, showing a higher proportion of EdU LRCs within the Vcam1^+^ population than within the Nestin^+^ population across both regions.

Taken together, these data suggest that Vcam1 consistently identifies NSCs from embryonic stages to adulthood; however, it signifies a specific subset with characteristics not entirely aligned with the overall progenitor population, especially concerning their relation to quiescence and the preservation of NSCs.

### Generation of the Vcam1-CreER^T2^ allele for *in vivo* fate mapping of individual Vcam1^+^ NSCs in the mouse brain

To examine the fate of Vcam1^+^ neural progenitors, we constructed a *Vcam1-CreER^T2^* knockin allele and bred the mice with the Ai9 reporter line (Fig. 2A, B). In this *Vcam1-CreER^T2^; Ai9* mouse line (designated as V-A), we could specifically label Vcam1^+^ progenitors in a temporally controlled manner by tamoxifen (TAM) injection. Each brain was serially sectioned for immunostaining, and each clone was three dimensionally reconstructed in its entirety (Fig. 2C) to recover all labeled cells.

**Figure 2.**
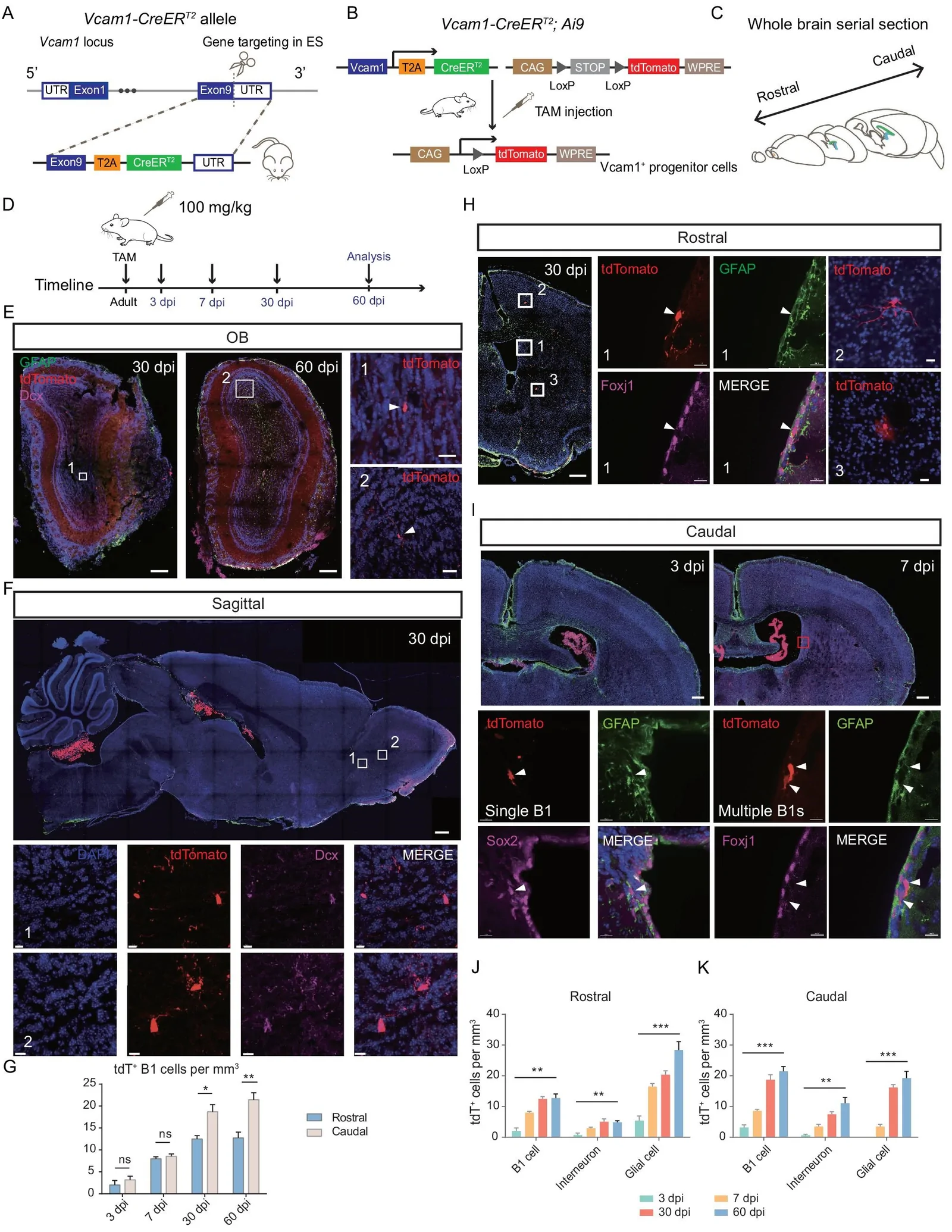
Vcam1-CreER*^T2^* Marks Adult NSCs in the V–SVZ. (A) Strategy for generating the *Vcam1-CreER^T2^* allele. (B) Strategy of intersectional lineage analysis using *Vcam1-CreER^T2^; Ai9* mouse line. (C) Serial sectioning of the whole brain from rostral to caudal direction for 3D reconstruction of all labeled clones. (D) Adult *Vcam1-CreER^T2^; Ai9* mice were given a single injection of tamoxifen (TAM) for fate mapping at population level at different time points in (E)–(K). (E) Confocal images of tdT^+^ interneurons in the OB at 30 dpi and 60 dpi (left) with higher magnification of the boxed area (right). Low-magnification image is a projection image of multiple sections, and the high-magnification images show a subset of *z* sections for colocalization. Arrowheads signify the tdT^+^ neuroblasts with long radial process. Scale bars, 50 μm (left) and 10 μm (right). (F) Confocal images of tdT^+^ interneurons in the OB from sagittal view (upper) at 30 dpi. The boxed area is shown at a higher magnification (lower). TdT^+^ cells in box 1 and box 2 are positive for Dcx, indicating the identity of immature neuroblasts migrating to the OB. Scale bars, 200 μm (upper) and 10 μm (lower). (G) The quantification of the tdT^+^ population in the rostral and caudal sections is shown. Values represent mean ± SEM (*n* = 3 mouse brains, at least three sections in each region are quantified in each brain). (H) Confocal images of tdT^+^ cells in the rostral brain section at 30 dpi (left). The boxed area is shown at a higher magnification (right). Box 1 contains Gfap^+^FoxJ1^−^ B1 cells lining the lateral ventricle (top right, arrows). Box 2 contains a mature interneuron in the cortex (bottom right) and box 3 contains a glial cell in the ventral area near the ventricle (bottom right). Scale bars, 200 μm (left) and 10 μm (right). (I) Confocal images of tdT^+^ cells in the caudal brain section at 3 dpi (left) and 7 dpi (right). Magnification of the boxed area shows a single Gfap^+^Sox2^+^ B1 cell with apical endfeet anchored to the ventricle at 3 dpi (bottom left). Boxed area at 7 dpi contains multiple Gfap^+^FoxJ1^−^ B1s with same apical endfeet lining the lateral ventricle. Scale bars, 200 μm (top) and 10 μm (bottom). (J) Quantification of the tdT^+^ population composition in rostral sections. Values represent mean ± SEM (*n* = 3–4 mice in each group, ^∗∗^*P* < 0.01, ^∗∗∗^*P* < 0.001; Two-way ANOVA with Tukey’s multiple comparison test). (K) Quantification of the tdT^+^ population composition in caudal sections. Values represent mean ± SEM (*n* = 3–4 mice in each group, ^∗∗^*P* < 0.01, ^∗∗∗^*P* < 0.001; Two-way ANOVA with Tukey’s multiple comparison test).

To validate the accuracy and Cre-mediated recombination efficiency in *Vcam1-CreER^T2^; Ai9* mice, we first screened the mouse line and identified that *Vcam1-CreER^T2^; Ai9* did not display any leaky tdT expression in the entire forebrain in the absence of tamoxifen injection (*n* = 6 animals). Then, we performed a single dose of 50 mg/kg of tamoxifen induction at E10.5, and analyzed brain slices 24 h post-induction (1 dpi) to ensure that the cells had just been labeled. We identified the cells by immunostaining for Sox2 and Vcam1. All tdT -labeled cells exhibited clear Vcam1 expression along their apical endfeet and were uniformly positive for the RGC marker Sox2 (Fig. S3A). These validations reinforce the specificity of our lineage tracing model.

To ensure unequivocal clonal analysis, we adjusted the TAM dose to achieve very sparse labeling in different developmental stages. In the lineage tracing experiments during the embryonic stage, we used a single dose of 20 mg/kg of tamoxifen for induction. After tamoxifen induction on V-A mice at different embryonic stages, we counted the number of labeled clones at 1 day post induction (1 dpi). We found that 20 mg/kg of tamoxifen resulted in sparse labeled 5–15 cell clones per hemisphere, and the number of clones did not change over time (Fig. S3B, *n* = 35 animals in total). In the long-term clonal lineage tracing experiments, we used a single dose of 10 mg/kg of tamoxifen for induction at E13.5 and E17.5, separately, and verified the number of clones by performing whole-brain imaging by light-sheet imaging after brain clearing (Videos S2, S3), then we statistically analyzed the number of clones and distances between different cells within the clones (Fig. S3C, *n* = 20 animals in total).

To analyze the confidence of clonally labeled cells in each clone, we have applied nearest neighbor distance (NND) analysis to the three-dimensional reconstruction data sets [21,22]. We used Imaris for automatic cell recognition and NND analysis in three-dimensional images of each hemisphere. The laterally migrated cluster was defined as the columnar/radial assembly of neurons perpendicular to the neocortical pial surface. The rostrally migrated cluster was defined as the rostral assembly of neurons along the rostral migratory stream (RMS). For detecting laterally migrated clusters, a rectangular cuboid with a lateral width of 200 μm (*X*-axis) and a vertical length corresponding to the radial length of the cortex between the pial surface and the VZ (600–800 μm at different embryonic stages, 1000 μm at P7 and 1200 μm at P21-30; *Y*-axis) was used to scan through every three consecutive sections (45 μm each; *Z*-axis) along the pial surface. For rostrally migrated cells, a similar process was used involving cylinders with a cylindrical length of 1000 μm (*X*-axis) and a diameter of 200 μm (*Y*-axis). We compared the data sets of clonally labeled progenies with spatially random simulated data sets containing the same number of data points within the same volume repeated 100 times. The NND distribution of clonally labeled progenies was shifted toward shorter distances compared with that of the random simulation data sets (Fig. S3D, E), suggesting that each cluster represents a clone that arises from a single neural progenitor. We further established surfaces for the cells and discovered that the processes of cells within the same cluster exhibit mutual contact, thereby validating the reliability of our clustering analysis criteria (Fig. S3F; the image shown is from Fig. 3G, section 18, as an example; Video S1).

**Figure 3.**
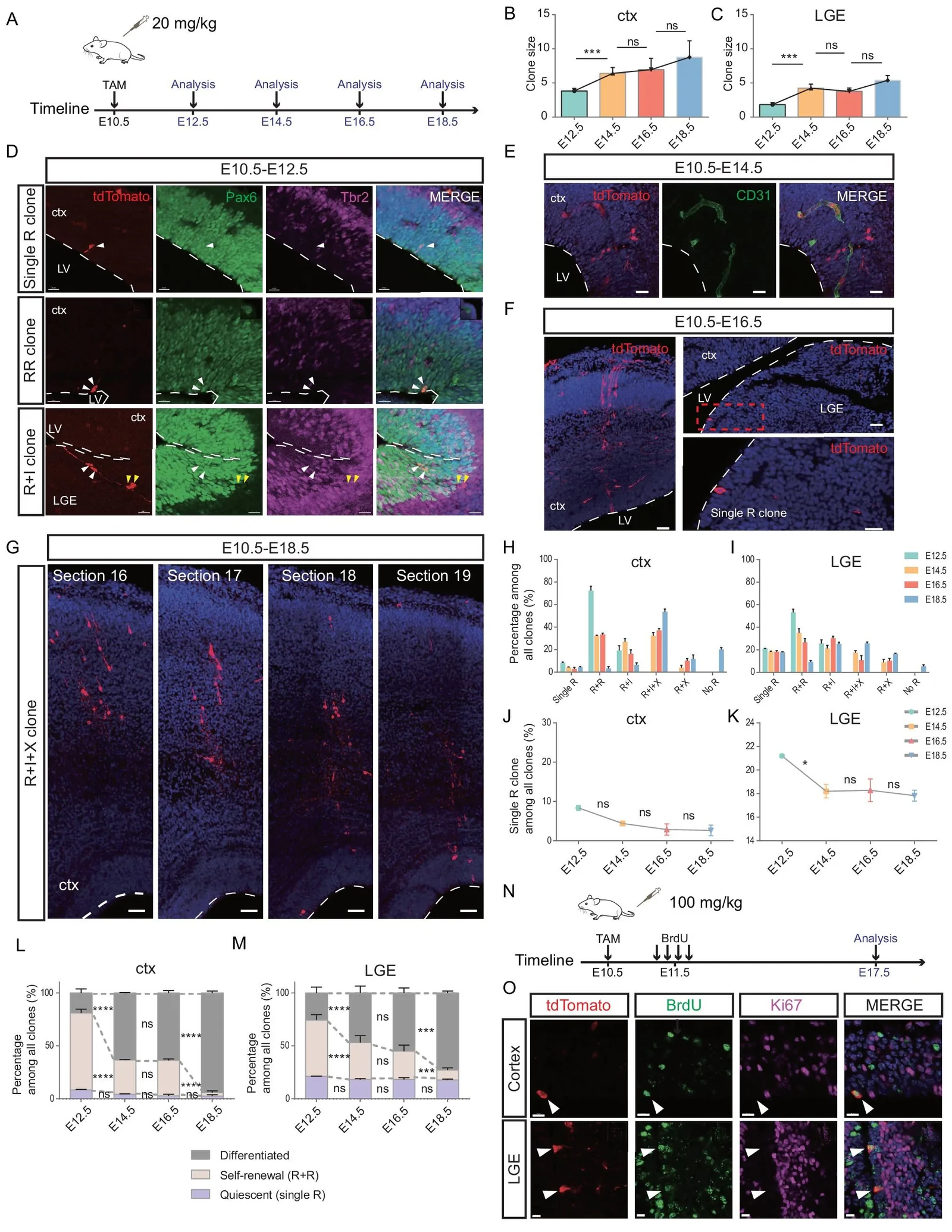
Clonal Lineage-Tracing of Vcam1^+^ Neural Precursors in the Embryonic Cortex and LGE. (A) Schematic illustration of the time-couse assay in embryonic period. Timed pregnant *Vcam1-CreER^T2^; Ai9* mice were given a single injection of tamoxifen (TAM) to label embryos at E10.5 for clonal lineage-tracing analysis in (B)–(M). (B and C) The quantification of the clone size of TAM induced tdT^+^ clones in the cortex (B) and GE (C). Values represent mean ± SEM (*n* = 6–8 brains; ***P < 0.001; unpaired *t* test). (D) Confocal images of clones at E12.5 with Pax6 and Tbr2 immunostaining. Images in the first row contain a single R clone consisting of a Pax6^+^ RGC in the VZ, and the arrow indicates the RGC with apical endfeet. Images in second row show an RR clone consisting of two Pax6^+^ RGCs (arrowheads). Images in the bottom row contain a clone consisting of Pax6^+^ RGCs and Tbr2^+^ IPCs, with arrowheads marking the Pax6^+^ RGCs and the Tbr2^+^ IPCs separately. Dashed lines depict the borders of the lateral ventricle. Scale bar, 20 μm. (E) Confocal images of a clone at E14.5 with CD31 immunostaining. Dashed lines depict the borders of the lateral ventricle. Scale bar, 20 μm. (F) Confocal images of two clones at E16.5, one containing RGCs, IPCs and neurons in the cortex (left), and the other containing only one RGC in GE (right). The boxed area (top right) is shown at a higher magnification (bottom right). Dashed lines depict the borders of the lateral ventricle. Scale bar, 50 μm (left, top right) and 20 μm (bottom right). (G) Confocal images of a clone at E18.5. Consecutive brain sections were stained for tdTomato and with DAPI. Scale bar, 50 μm. (H and I) Quantifications of the percentage of clone compositions in the cortex (H) and GE (I). Clones were classified into six categories: single R (1 RGC only), RR (≥2 RGCs only), R + I (≥1 RGC and ≥1 IPC), R + I + X (≥1 RGC and ≥1 IPC; ≥1 neuron or glial cell), R + X (≥1 RGC and ≥1 neuron or glial cell); No R (no RGC). Values represent mean ± SEM (*n* = 3–8 brains; 20–60 total clone numbers). (J and K) Quantification of the percentage of single R clones in the cortex (J) and GE (K). Values represent mean ± SEM (*n* = 3–8 brains; 20–60 total clone numbers, ^∗^*P* < 0.05; One-way ANOVA with Tukey’s multiple comparison test). (L and M) Quantifications of the percentage of clone compositions in the cortex (L) and GE (M). Clones were further classified into three categories: quiescent (single R clones); self-renewal (RR clones); differentiated (consisting of R + I; R + I + X; R + X; No R clones). Values represent mean (*n* = 3–8 brains; 20–60 total clone numbers, ^∗∗∗^*P* < 0.001, ^∗∗∗∗^*P* < 0.0001; Two-way ANOVA with Tukey’s multiple comparison test). (N) Schematic illustration of the tamoxifen induction assay. (O) Representative images of tdTomato, BrdU and Ki67 immunostaining in the cortex and LGE. Scale bar, 10 μm. TAM, tamoxifen.

Since Vcam1 was previously shown to be highly enriched around the blood vessels in VZ [23], we stained CD31 to eliminate the interference of blood vessel signal with the clonal signal of progeny cells (Fig. S3G). Taken together, these results suggest that the V-A mouse line enables us to precisely map the fate of individual Vcam1^+^ cells in the process of development.

### Vcam1-CreER^T2^ marks adult NSCs with limited self-renewal and multilineage differentiation capacity in the V–SVZ

Adult NSCs (B1 cells) reside within the walls of the lateral ventricles. A small percentage of B1 cells self-renew to maintain the stem cell pool, while most B1 cells give rise to neuroblasts that migrate a long distance to the OB, where they differentiate into multiple types of inhibitory interneurons [2,24,25].

To assess the heterogeneity of Vcam1 expression in B1 cells, we first integrated published single-cell RNA sequencing data of NSCs from the adult V–SVZ [26]. Uniform Manifold Approximation and Projection (UMAP) visualization and quantitative analysis revealed the distribution and proportion of Vcam1-expressing cells across different cell types in the V–SVZ. Vcam1 was found to be predominantly expressed in B cells and astrocytes, with minor expression in ependymal cells and C cells (Fig. S4A, B). Within the B cell cluster, 52.00% of cells were Vcam1^+^, underscoring a direct association between Vcam1 expression and the adult B1 NSC population.

We also examined the expression patterns of two other established NSC markers, CD133 (Prom1) and Egfr, in the same dataset. Egfr was mainly localized to the C cell cluster, with lower expression in mitotic cells and B cells (Fig. S4A, B), supporting its role as a marker for activated NSCs and their progeny, such as C cells. In contrast, Prom1 was highly expressed in endothelial cells, followed by ependymal cells and mitotic cells, and to a lesser extent in B and C cells (Fig. S4A, B), indicating its utility in labeling ependymal cells situated in close proximity to B1 cells, along with a subset of B and C cells. Among B cells, 28.97% were Egfr^+^ and only 10.79% were Prom1^+^, demonstrating that Vcam1 exhibits higher specificity for B1 cells compared to these markers.

Given the membrane localization of CD133 and Egfr, which complicates accurate quantification in tissue sections, we performed acute dissociation and staining of V–SVZ tissues from mice at different postnatal ages to precisely determine the proportion of Vcam1^+^ cells among CD133^+^ and Egfr^+^ populations (Fig. S4C–E). We observed that the percentage of Vcam1^+^ cells within both CD133^+^ and Egfr^+^ populations increased with age—from 28.26% to 59.25% in CD133^+^ cells, and from 27.55% to 57.75% in Egfr^+^ cells—with the most pronounced rise occurring between P7 and P21 (Fig. S4E). These findings suggest that the proportion of Vcam1-expressing cells among key NSC marker-positive populations increases during postnatal development, reaching a plateau in adulthood. Single-cell expression analysis further confirmed that Vcam1 served as a more specific marker for B1 cells than CD133 or Egfr.

To directly compare the percentage and behavioral differences of the Vcam1^+^ subpopulation within the B1 pool, we re-analyzed the single-cell dataset from Cebrian-Silla, Nascimento *et al.* (2021). Compared with other classical B1 cell markers (e.g. Nestin, Prom1, Egfr), Vcam1 exhibited the highest positive ratio in B1 cells, indicating that Vcam1^+^ B1 cells represent the major subpopulation of adult B1 cells (Fig. S4F). We then analyzed proliferative activity by measuring the percentage of Ki67^+^ cells within each marker-defined B1 subpopulation. Strikingly, the Ki67^+^ ratio in Vcam1^+^ B1 cells was significantly lower than that in B1 cells positive for Nestin, Prom1, or Egfr (Fig. S4G), suggesting that Vcam1^+^ B1 cells are a more quiescent NSC subpopulation residing in the adult brain.

To examine the fate of Vcam1-expressing NSCs in the adult forebrain subventricular zone (V–SVZ), adult V-A mice (8 weeks old) were administered a single high dose of tamoxifen (100 mg/kg) and analyzed at 3, 7, 30, and 60 days postinjection (dpi) (Fig. 2D).

We first performed immunostaining on OB samples from injected V-A mice (Fig. 2E). A small number of tdT^+^ neuroblasts (1–6 cells per OB) exhibiting extended long processes were observed at both 30 dpi (*n* = 4 animals) and 60 dpi (*n* = 3 animals), indicating that Vcam1^+^ cells can function as adult NSCs and generate OB interneuron lineage. Similarly, tdT^+^ cells were detected in sagittal OB sections at 30 dpi (Fig. 2F). These cells co-expressed doublecortin (Dcx), a marker of neuroblasts and immature neurons, confirming their identity as immature OB neuroblasts (Fig. 2F).

For population-level fate mapping, we quantified the number and types of tdT^+^ cells in coronal sections across the whole brain. Analysis of tdT^+^ B1 cell density (cells per mm³) revealed a time-dependent increase in both rostral and caudal regions, with a higher distribution in caudal sections at 30 and 60 dpi (Fig. 2G). In rostral sections at 30 dpi, we identified Gfap^+^ B1 cells along the lateral ventricular walls (Foxj1-negative, excluding ependymal identity), as well as tdT^+^ interneurons in the striatum and glial cells (identified by morphology), demonstrating the multilineage differentiation potential of Vcam1^+^ B1 cells (Fig. 2H). In caudal sections, most tdT^+^ signal localized to the choroid plexus, consistent with known Vcam1 expression on the apical surface of choroid plexus epithelial cells [27].

At 3 dpi, we observed only single Gfap^+^Sox2^+^ B1 cells with apical endfeet attached to the ventricular surface. By 7 dpi, however, clones containing more than one Gfap^+^Foxj1^–^ B1 cell were detected, with endfeet anchored at shared ventricular points, indicating self-renewal capacity of Vcam1^+^ B1 cells (Fig. 2I). Due to the relatively low labeling efficiency of B1 cells in adulthood (resulting in only 10–20 tdT^+^ B1 cells per mm³ by 60 dpi), we focused on quantitative analysis of tdT^+^ cell population densities across groups rather than detailed clonal dynamics.

In both rostral and caudal sections, the densities of B1 cells, interneurons, and glial cells increased over time (Fig. 2J, K). Together, these results demonstrate that Vcam1^+^ cells in the adult V–SVZ exhibit self-renewal capacity and multilineage differentiation potential within the short-term tracing window of 3–60 days.

### Embryonic Vcam1^+^ radial glia exhibit dual fates with early emergence of a quiescent pool

Our immunofluorescence analysis in *Vcam1* reporter mouse showed that Vcam1 was expressed from an early embryonic stage (E10.5) and continuously expressed during embryonic development in both cortex and LGE. To examine the behavior of Vcam1^+^ RGCs during embryonic development, we performed clonal lineage tracing using the *Vcam1-CreER^T2^; Ai9* mouse line. We injected timed pregnant female V-A mice with tamoxifen to label embryos at E10.5 and optimized the tamoxifen dose for clonal lineage tracing. Each brain was serially sectioned for immunostaining, and each clone was three dimensionally reconstructed for analysis (Video S4).

To delineate the lineage of Vcam1^+^ RGCs and examine their clonal properties, we performed time-course analyses by using *Vcam1-CreER^T2^; Ai9* mouse line. A single injection of tamoxifen (20 mg/kg) at E10.5 resulted in sparse labeled cell clusters at E12.5 (5–10 cell clones per hemisphere, *n* = 12 brains). Brains were analyzed at E12.5, E14.5, E16.5 and E18.5, respectively (Fig. 3A). Clone size and composition were examined separately in cortex and LGE (abbreviated as GE in the following text). Time-course analysis showed a significant increase of clone size in early embryonic stage between E12.5 and E14.5, but such an increase in late embryonic developmental process after E14.5 was not significant in both cortex and GE (Fig. 3B and C). These results indicated a short period of rapid proliferation and differentiation in the early development but slowly dividing trend in the late stage of Vcam1^+^ cell lineage. Remarkably, clone size in GE was significantly smaller than cortex in every analyzed embryonic stage, suggesting a relatively lower dividing capacity of GE Vcam1^+^ RGCs (Fig. 3B and C). We classified the composition of induced clones into six categories: single R (1 RGC only), RR (≥2 RGCs only), R + I (≥1 RGC and ≥1 IPC), R + I + X (≥1 RGC and ≥1 IPC; ≥1 neuron or glial cell), R + X (≥1 RGC and ≥1 neuron or glial cell); No R (no RGC). At E12.5 (2 dpi), we observed single R clones consisting of only 1 Pax6^+^ RGC, RR clones with more than 1 Pax6^+^ RGCs with apical endfeet anchored to the same position, and R + I clones containing Tbr2^+^ (cortex) or Ascl1^+^ (GE) IPCs (Fig. 3D). At E14.5 and E16.5, with increased clone size, cells with neuronal identity appeared (based on morphology and position), and vascular signals were excluded by CD31 immunostaining (Fig. 3E and F). Intriguingly, single R clones could be observed at late embryonic stage, indicating a proportion of Vcam1^+^ cells enter quiescent state during embryonic development (Fig. 3F). At E18.5, we observed clones with wider distribution of progeny cells (Fig. 3G). Moreover, No R clones appeared at E18.5 in both cortex and GE, suggesting that a number of Vcam1^+^ RGCs were depleted at late developmental stage. We analyzed the percentage of different types of clones during the time course, and clones with differentiated cells were increasing in the proportion with the developmental process (Fig. 3H and I). Strikingly, we identified a proportion of single R clones in both cortex and GE that continuously presented during development. In these two regions, the proportion of single R clones significantly decreased during early embryonic time, but maintained at a percentage until late development (2.71% ± 1.38% in cortex;17.38% ± 0.44% in GE) (Fig. 3J and K). Moreover, we observed a large percentage of R + R clones among all clones from E12.5 to E16.5 which decreased dramatically at E18.5, indicating that a large percentage of Vcam1^+^ cells kept in a state of self-renewal to expand stem cell population from early to middle stage (Fig. 3L and M). In addition, a significantly higher percentage of quiescent and self-renewal clones were retained until late development in GE (25.93%) compared with cortex (9.09%), consistent with results that late embryonic RGCs in the GE are the embryonic origin of most young adult NSCs [9].

To further confirm the emergence of quiescent Vcam1^+^ RGCs at the early embryonic stage, we injected 20 mg/kg of tamoxifen at E10.5, then injected BrdU four times with 2-h intervals at E11.5 and sacrificed embryos at E17.5. BrdU label retention was detected in the labeled tdT^+^ cells along the ventricular surface in both the cortex and LGE region (Fig. 3N). We have observed tdT^+^ single R clones co-labeled with BrdU without Ki67 expression in these two regions, indicating at least a subset of tdT-labeled cells acquire quiescence before E13.5 (Fig. 3O).

To characterize the developmental properties of Vcam1^+^ RGCs across embryonic stages, we administered tamoxifen (50 mg/kg) to timed-pregnant V-A mice at E10.5, E12.5, E14.5, and E16.5, and collected brain tissues after 2 days post-induction (dpi) for each group (Fig. S5A, B). We analyzed clone size following the same labeling-to-analysis interval to assess the proliferative capacity of Vcam1^+^ cells across developmental time. Clone size progressively decreased from E10.5 to E16.5, indicating a developmental decline in the dividing activity of Vcam1^+^ RGCs (Fig. S5C, D). Furthermore, clones in GE were consistently smaller than those in the cortex, suggesting regionally distinct proliferation dynamics, with GE Vcam1^+^ RGCs exhibiting lower dividing capacity.

We next examined the composition of clone types in the cortex and GE. Notably, by late embryogenesis, the two regions displayed divergent clonal fate distributions. In the E16.5 cortex, ∼60% of clones comprised slowly dividing (single R) or self-renewing (RR) RGCs, whereas the rest clones underwent rapid differentiation (Fig. S5E). In contrast, over 80% of clones in the GE maintained a slowly dividing or self-renewing state within the 2-day tracing window (Fig. S5F).

These findings suggest that Vcam1^+^ RGCs constitute a heterogeneous population during embryogenesis, encompassing NSCs with distinct fate tendencies. The differential distribution of these subpopulations between the cortex and GE underlies regional disparities in the preservation of adult NSCs, ultimately influencing the size of the seed cell pool retained in each region.

To directly assess whether the relatively quiescent behavior observed in Vcam1^+^ progenitors is a shared property among NSC populations or represents a distinct lineage feature, we performed parallel clonal lineage tracing using *Nestin-CreER^T2^; Ai9* mice as a direct comparator (Fig. S5G–H). Nestin is broadly expressed in neural progenitors and serves as a reference for the general progenitor pool. By directly comparing Vcam1^+^ and Nestin^+^ lineages under identical experimental conditions, we sought to determine whether Vcam1^+^ progenitors exhibit unique proliferative and differentiative properties relative to a broader progenitor population. First, we analyzed clone size as a readout of proliferative capacity across different brain regions (Fig. S5I). At E12.5, no significant difference in clone size was observed between Vcam1^+^ and Nestin^+^ lineages in either the cortex or GE. However, by E18.5, Nestin^+^ progenitors generated significantly larger clones than Vcam1^+^ cells in both regions, indicating that the Nestin^+^ population possesses greater proliferative capacity over longer developmental timescales. Second, we characterized the composition of all traced clones based on cellular phenotypes, following the same classification scheme used in Fig. 3 (single R, R+R, R+I, R+I+X, etc.). At E12.5 (2 days post-labeling), Nestin^+^ cells already gave rise to a significantly higher proportion of differentiated clones (R+I category) in both cortex and GE compared to Vcam1^+^ cells. Notably, in the GE, Nestin^+^ clones showed a significantly lower proportion of single R clones (quiescent-like radial glia) relative to Vcam1^+^, suggesting that Vcam1^+^ progenitors retain a more quiescent state even at early stages (Fig. S5J, top). By E18.5, in the cortex, Nestin^+^ cells produced significantly more differentiated clones (R+I+X) than Vcam1^+^ cells, further supporting that a higher fraction of Nestin^+^ progenitors adopt differentiation fates. In the GE, even more striking differences emerged: Nestin^+^ clones maintained significantly fewer single R clones compared to Vcam1^+^ clones, accompanied by significantly higher proportions of proliferative (R+R) and differentiated (R+I+X and no R) clones (Fig. S5J, bottom). Together, these comparative data demonstrate that, relative to the broader Nestin-expressing progenitor pool, Vcam1^+^ progenitors exhibit a significantly more quiescent character and reduced proliferative and differentiative capacity over embryonic development. This functional divergence supports the conclusion that the quiescence-like behavior observed in Vcam1^+^ lineage is not a generic property of all neural progenitors but rather a distinctive feature of the Vcam1^+^ subpopulation, which may contribute to regionally distinct NSC pool maintenance.

### Long-term fate mapping defines temporal dynamics of embryonic Vcam1^+^ NSC pools

Vcam1 was previously shown to be expressed in RGCs of the developing cerebral cortex and GE, and the expression was retained throughout the continuum of embryonic RGCs to pre-B1 cells and then to the adult quiescent NSCs [17,18]. Consistent with these findings, immunohistological analysis in our *Vcam1* reporter mouse line confirmed sustained Vcam1 expression in a subpopulation of NSCs from embryonic stages to adulthood (Fig. 1). These observations raised the possibility that the *Vcam1-CreER^T2^* line also might be used as a tool to enrich targeting RGCs to trace the potential embryonic origin of postnatal and adult NSCs in V–SVZ.

To directly assess whether embryonic Vcam1^+^ RGCs give rise to adult B1s, we performed high-dose tamoxifen injection at E13.5 and E17.5, respectively, then analyzed their fate at the population level at P60 (adult) (Fig. S6A). Consistent with our previous results, we found tdT^+^ cells in OB, RMS, V–SVZ, and thalamus (Fig. S6B and C). Gfap^+^ tdT^+^ cells were observed in the V–SVZ (Fig. S6C, Box1), suggesting that Vcam1-expressing cells give rise to NSC lineage cells and persist as adult NSCs. In addition, a subset of tdT⁺ cells was found within the V–SVZ but at a distance from the ventricular surface. These cells were Gfap^−^, lacked ventricular contact, and exhibited chain-like migration along the rostral axis, identifying them as neuroblasts. This population was thus distinct from Gfap⁺ B1 cells, which resided with their cell bodies in the SVZ and maintained contact with the ventricle (Fig. S6C Box1). Meanwhile, Dcx^+^ tdT^+^ cells were observed in the RMS toward OB, indicating their identity of neuroblasts. We further examined the percentage of those tdT^+^ cell types (Fig. S6C Box2). In embryonic development, E13.5 labeled Vcam1^+^ cells could differentiate into a large number of neurons and glial cells. In contrast, E17.5 labeled Vcam1^+^ RGCs switch to generating more glia cells (Fig. S6D and E). A larger percentage of tdT^+^ B1 cells originated from E17.5 labeled Vcam1^+^ RGCs were observed at P60, suggesting more Vcam1^+^ RGCs at late embryonic stage transformed into slowly proliferating seed cells of adult NSCs.

To delineate the lineage trajectories of these embryonic origin seed cells, we conducted long-term clonal lineage tracing of Vcam1^+^ RGCs labeled at E13.5 and E17.5, analyzing whole-brain serial sections at P7 and P30. Sparse labeling was achieved using a low tamoxifen dose (10 mg/kg) to ensure unambiguous clonal resolution (Fig. 4A, D).

**Figure 4.**
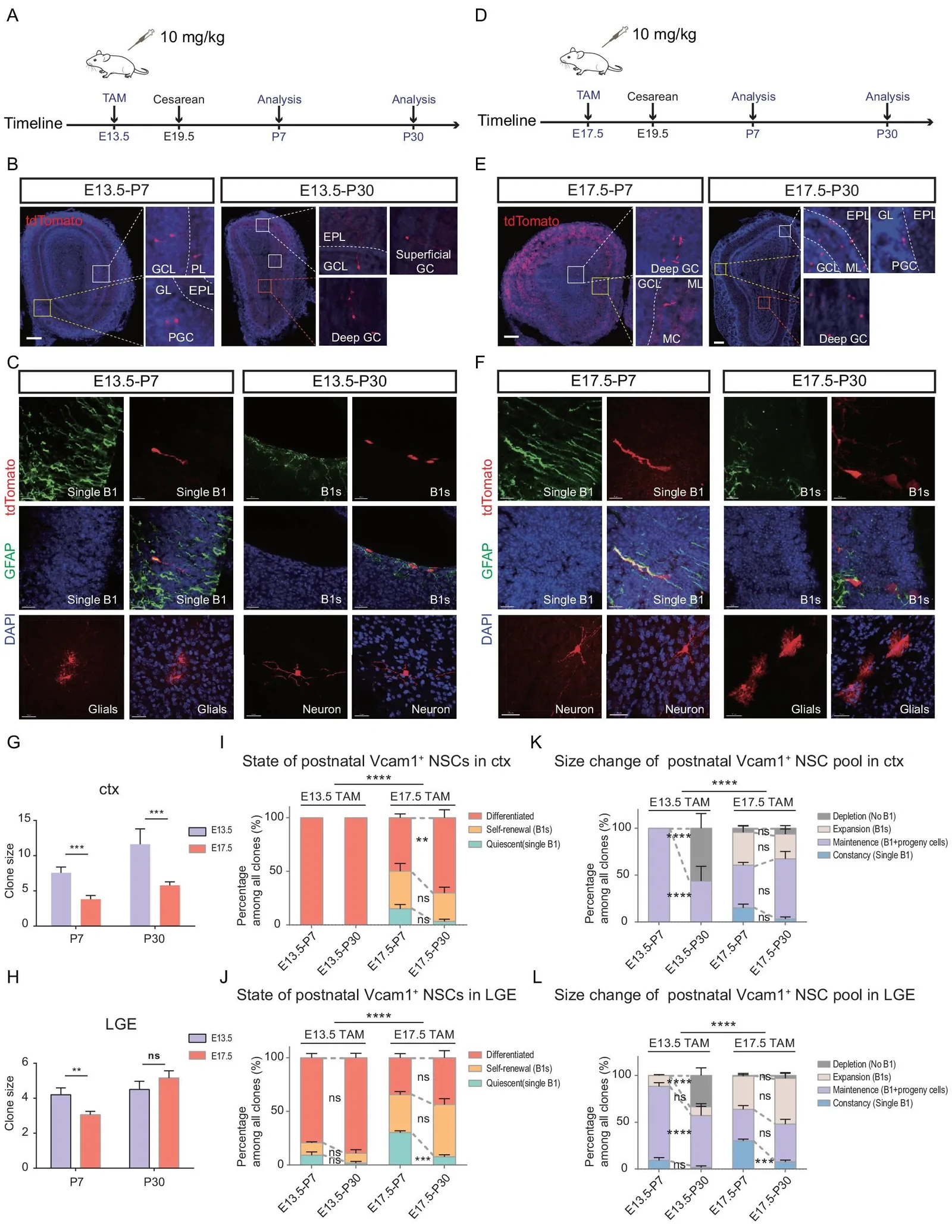
Embryonic Vcam1^+^ precursors give rise to adult NSCs in the V–SVZ (A) Schematic illustration of the long-term clonal lineage tracing from embryonic to postnatal days. Timed pregnant *Vcam1-CreER^T2^; Ai9* mice were given a single injection of tamoxifen (TAM) to label embryos at E13.5, brains were analyzed at P7 or P30 for clonal analysis (B)–(D). (B) Confocal images of tdT^+^ interneurons in the OB at P7 and P30 with higher magnification of the boxed area (right). Dashed lines depict the borders of layers in olfactory bulb. Scale bars, 200 μm. (C) Confocal images of tdT^+^ cells in the coronal sections at P7 and P30. Single B1 and B1 cells lining the lateral ventricle are Gfap^+^. Neurons in cortex and glial cells in the ventral area near the ventricle are identified by morphology. Scale bars, 15 μm. (D) Schematic illustration of the long-term clonal lineage tracing from embryonic to postnatal days. Timed pregnant *Vcam1-CreER^T2^; Ai9* mice were given a single injection of tamoxifen (TAM) to label embryos at E17.5, and brains were analyzed at P7 or P30 for clonal analysis (B)–(D). (E) Confocal images of tdT^+^ interneurons in the OB at P7 and P30 with higher magnification of the boxed area (right). Dashed lines depict the borders of layers in olfactory bulb. Scale bars, 200 μm. (F) Confocal images of tdT^+^ cells in the coronal sections at P7 and P30. Single B1 and B1 cells lining the lateral ventricle are Gfap^+^. Neurons in cortex and glial cells in the ventral area near the ventricular are identified by morphology. Scale bars, 15 μm. (G and H) Quantifications of the clone size in the cortex (G) and GE (H) in four experimental groups. Values represent mean ± SEM (*n* = 18 brains; 147 total clone numbers; ***P < 0.001; unpaired t test). (I and J) Quantifications of the percentage of clone compositions in the cortex (I) and GE (J). Clones were further classified into three categories: quiescent (single B1 clones); self-renewal (B1s clones); differentiated (consisting of B1 + I; B1 + I + X; B1 + X; No B1 clones). Values represent mean ± SEM (*n* = 4–5 brains; 30–50 total clone numbers, ^∗∗^*P* < 0.005, ^∗∗∗^*P* < 0.0001; Two-way ANOVA with Tukey’s multiple comparison test). (K and L) Quantifications of the percentage of clone compositions in the cortex (K) and GE (L). Clones were further classified into three categories: constancy (single B1 clones); maintenance (consist of B1 + I; B1 + I + X; B1 + X clones); expansion (B1s clones); depletion (No B1 clones). Values represent mean ± SEM (*n* = 4–5 brains; 30–50 total clone numbers, ^∗∗∗^*P* < 0.001, ^∗∗∗∗^*P* < 0.0001; Two-way ANOVA with Tukey’s multiple comparison test).

In animals labeled at E13.5, tdT^+^ interneurons were observed in the OB at both P7 and P30 (Fig. 4B). These included multiple interneuron subtypes—such as deep and superficial granule cells, and periglomerular cells—reflecting the regional diversity of their embryonic Vcam1^+^ RGC origins (Fig. 4B). In forebrain sections, tdT^+^ Gfap^+^ B1 cells were detected along the lateral ventricular walls at P7 and P30, confirming a direct lineage from E13.5 RGCs to postnatal B1 cells (Fig. 4C). We also observed tdT^+^ neurons and glial cells in brain parenchyma, indicating that E13.5 Vcam1^+^ RGCs contribute to both embryonic neurogenesis and gliogenesis (Fig. 4C).

We next analyzed clones labeled at E17.5 (Fig. 4D–F). Similarly, diverse tdT^+^ OB interneurons were present at P7 and P30, demonstrating that late embryonic Vcam1^+^ cells continue to produce OB interneuron lineages postnatally (Fig. 4E). In forebrain sections, we identified B1 cells with apical endfeet anchored to the ventricle, alongside interneurons and glial cells, confirming that Vcam1^+^ RGCs at E17.5 directly give rise to postnatal B1 cells, glia, and OB interneurons (Fig. 4F). Notably, a substantial fraction of clones contained multiple B1 cells with apical endfeet clustered at the same ventricular position, indicating persistent self-renewal capacity of Vcam1^+^ RGCs in the postnatal period (Fig. 4F).

We systematically analyzed the clone size and further classified clonal compositions into six categories and integrated data from E13.5 and E17.5 labeling induction timepoints to compare cortical and GE Vcam1^+^ lineages (Fig. 4G–L). Clone size increased from P7 to P30 in both E13.5- and E17.5-labeled cortical clones, consistent with continued proliferation over time (Fig. 4G). In the GE, clone size also increased from P7 to P30 in the E17.5 labeled clones, but remained stable in the E13.5 labeled clones, suggesting that early-labeled GE RGCs largely complete their expansion before P7 (Fig. 4H).

Clonal composition analysis revealed that in the cortex, all E13.5-labeled Vcam1^+^ clones had differentiated by P7, whereas E17.5-labeled clones retained a significant proportion of quiescent or self-renewing B1 cells at both P7 and P30 (Fig. 4I). This indicates that Vcam1^+^ cortical RGCs labeled later in embryogenesis are more likely to be retained as adult NSCs. In the GE, both E13.5- and E17.5-induced clones contained quiescent and self-renewing B1 cells, though the latter group exhibited a higher proportion of undifferentiated clones (Fig. 4J).

We further tracked the dynamics of labeled NSC pools from embryogenesis to postnatal stages. In the cortex, half of the E13.5-labeled B1 cells were lost by P30, whereas E17.5-labeled B1 cells were largely maintained or expanded through self-renewal (Fig. 4K). In the GE, most E13.5-labeled B1 cells persisted at P7, but ∼40% were lost by P30. In contrast, E17.5-labeled B1 cells in the GE were largely maintained or increased in number, underscoring their role as a durable source of adult NSCs (Fig. 4L).

To directly address whether Vcam1-derived adult NSCs have experienced quiescence, we performed a BrdU pulse-chase experiment. At E15.5, we administered Tamoxifen to *Vcam1-CreER^T2^; Ai9* embryos to induce tdT labeling in Vcam1^+^ cells, followed by continuous BrdU injections at E16.5 to label proliferating cells. At P21, we analyzed Tdt^+^Sox2^+^ B1 cells in the lateral ventricle (Fig. S6F). Consistent with Fig. 4, we identified single B1 clones (isolated tdT^+^Sox2^+^ cells without progeny), which have not divided from E15.5 to P21 and are BrdU^−^, directly demonstrating a Vcam1^+^ subpopulation that remains continuously quiescent and is retained as postnatal B1 cells (Fig. S6G, top). In addition, within multicellular clones containing both B1 cells and differentiated progeny, we observed a subset of tdT^+^Sox2^+^ B1 cells that retained BrdU at P21 (Fig. S6G, bottom). Although these Vcam1^+^ cells had undergone cell division, the persistent BrdU signal indicates they experienced only a transient proliferation around E16.5 and then entered sustained quiescence.

Taken together, our lineage tracing data points to a model in which the Vcam1⁺ NSC pool is dynamically replenished over developmental time. Vcam1^+^ cells labeled at E12.5 largely contribute to differentiation during embryonic and early postnatal development, yet approximately half of them are retained as B1 cells in the adult V–SVZ. In contrast, Vcam1^+^ cells labeled at E17.5 were largely maintained or even increased in number into adulthood, highlighting their role as a persistent source of adult NSCs. Among these cells labeled at E17.5, a subset remains quiescent, while others undergo only a transient proliferation before entering quiescence. In essence, the early RGC pool slowly differentiates yet preserves a substantial fraction as adult B1 cells, while the late pool is more efficiently maintained. The transition from the early pool to the late pool indicates that the reserve of Vcam1-derived NSCs is dynamically replenished during embryonic development, ensuring a sustainable source of NSCs for adult neurogenesis.

### Single-cell transcriptome profiling of Vcam1^+^ NSCs

To resolve the cellular heterogeneity of Vcam1^+^ RGCs, we microdissected the cortex and LGE from *CAG-Cre; Vcam1* reporter embryos at E13.5 and E17.5 respectively (Fig. 5A). Single-cell suspensions were prepared and tdT^+^ cells were isolated by FACS (Fig. 5A, B). Using the 10x Genomics Chromium platform, we generated single-cell RNA-seq (scRNA-seq) data for all samples, and additionally obtained matched scATAC-seq data for E17.5 cortical and GE cells. Due to technical limitations, scATAC-seq was not performed for E13.5 samples. After quality control, 36 893 high-quality tdT^+^ transcriptomes and 16 485 tdT^+^ epigenomes were retained (Fig. S7A and B).

**Figure 5.**
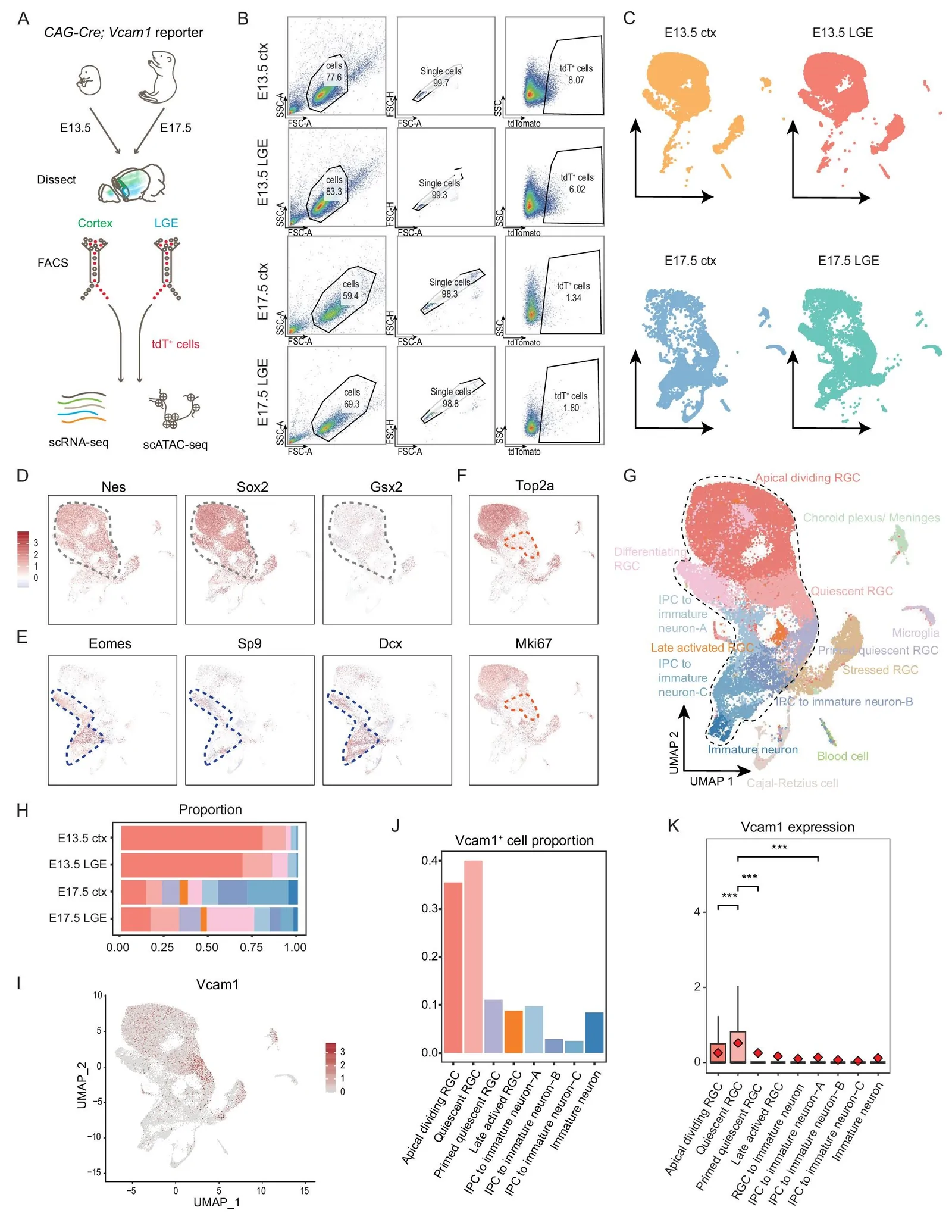
scRNA-Seq analysis of E13.5 and E17.5 Vcam1^+^ NSCs from cortex and LGE (A) Schematic of the workflow of scRNA-Seq and scATAC-Seq analysis, including sample preparation, cell sorting and library preparation. (B) Representative FACS plots showing the isolated single tdT^+^ cells (C) UMAP plots of cells from each sample. (D–F) Expression of marker genes associated with embryonic RGC (Nestin, Sox2, Gsx2); IPC (Eomes, Sp9); Newborn neuron (Dcx) on UMAP (G) UMAP of cells colored by cluster. (H) Bar charts showing cell clusters proportions of different samples. (I) Expression of Vcam1 on UMAP. (J) Bar plot showing the proportion of Vcam1^+^ cells across major neural cell types. (K) Box plot illustrating the distribution of Vcam1 expression levels across the major neural cell types. Red diamonds denote the mean expression value for each population. We reported the mean rather than the median here because the high proportion of Vcam1^−^ cells would obscure the expression trend in Vcam1^+^ populations when using the median. Statistical significance was assessed using a two-sided Wilcoxon rank-sum test. **P < 0.001.

UMAP visualization identified 19 transcriptionally distinct cell clusters (Fig. S7C). Cells within clusters showed high Rogue scores, reflecting strong intra-cluster consistency (Fig. S7D). Transcriptional profiles of tdT^+^ cells from cortex and GE at the same stage were highly similar (Figs 5C, S7E), suggesting shared molecular states across regions.

Cell-type markers analysis confirmed that most cells expressed RGC markers (Nestin, Sox2, Gsx2; Fig. 5D), while a subset expressed differentiation markers (Eomes, Sp9, Dcx; Fig. 5E). At E13.5, the majority of cells were RGCs, whereas by E17.5, a fraction retained RGC identity and others entered neurogenic trajectories (Fig. 5D–F). Notably, we also identified a subset of RGCs that did not express differentiated cell markers and proliferation markers (Top2a, Mki67), indicating the presence of a subpopulation of quiescent Vcam1^+^ RGCs (Fig. 5F).

Similar global gene expression patterns were observed in cell clusters composed of sorted cells from the cortex and GE at the same stage. To exclude the possible regional errors caused by manual dissection during library preparation, we compared the expression pattern of region-specific RGC markers and IPC markers in different samples (Fig. S7F). From the UMAP distribution, it can be observed that the region-specific RGC markers exhibit similar expression patterns among the cell clusters (Fig. S7F Left), supporting similarities of gene expression pattern between tdT^+^ RGCs from different brain regions. However, the expression patterns of region-specific IPC markers in the UMAP analysis exhibit a distinct distribution among cell clusters (Fig. S7F Right). We supposed that the different gene expression patterns of IPCs lie in the different proliferation abilities and the expression of general NSC markers of IPCs from different regions (e.g. Sox2, Nestin) in cortical IPCs compared to those in GE IPCs. The IPCs in GE are more concentrated in the differentiating RGC population we defined, indicating their transition from RGCs to newborn IPCs.

To improve cell-state annotation, we applied a gene-set-based classification using previously established markers (Table S1), regrouping the 19 clusters into 16 discrete populations with distinct molecular signatures (Figs 5G, S8A). Non-neurogenic lineages (Cajal-Retzius cells, choroid plexus/meninges, microglia, blood cells) and stressed RGCs within which mitochondrial genes account for a high percentage of expressed genes indicating cell death were excluded from further analysis.

According to the cell proportions in different samples, we observed that the apical dividing RGC population was greatly decreased, while populations with differentiation signatures were greatly increased from E13.5 to E17.5 in both regions (Fig. 5I). Quiescent RGC proportions were consistently higher in the LGE than cortex at both E13.5 (16.56% vs. 13.00%) and E17.5 (16.19% vs. 8.99%), aligning with lineage tracing data and supporting regional differences in adult NSC development (Fig. 5I).

We then verified expression of Vcam1 in the defined cell clusters. From the UMAP, Vcam1 is primarily distributed in the RGC clusters, especially in the quiescent RGC cluster (Fig. 5J). The proportion of Vcam1 expressing cells in different clusters did not exceed 50%, which can be attributed to potential RNA signal loss, and insufficient sequencing depth and stochastic RNA expression (Fig. 5J). RNA expression is often stochastic, turning on and off on a short time scale. RNA presence can therefore fluctuate even in cells that are steadily Vcam1 (tdT) protein positive. Interestingly, we found distinct proportions of Vcam1^+^ cells and Vcam1 expression levels in different clusters (Fig. 5J–K). Apical dividing and quiescent RGCs showed the highest Vcam1^+^ proportions and expression levels, whereas primed quiescent RGCs exhibited lower levels, and differentiated cells showed the lowest level (<10%; Fig. S8B, C). A significant Vcam1 expression drop occurred between quiescent and primed quiescent RGCs, suggesting Vcam1 downregulation precedes overt differentiation (Fig. 5K). Furthermore, the absence of Vcam1 expression persisted in differentiated cells, as evidenced by the significant difference in Vcam1 expression levels between quiescent RGCs and differentiated cells (Fig. 5K). Consistently, Vcam1 expression at both E13 and E17 was predominantly restricted to apical dividing and quiescent RGCs, with quiescent RGCs showing the highest expression levels (Fig. S8D, E). Accordingly, we hypothesized that cells in these clusters were Vcam1^−^ tdT^+^ cells derived from Vcam1^+^ cells that have recently lost Vcam1 expression but still retained tdT labeling. Therefore, cells in these clusters, as the progeny of Vcam1^+^ cells, can also reveal the lineage relationship of Vcam1^+^ cells at the single-cell level.

Differential gene expression analysis identified genes enriched in the quiescent RGCs cluster (Table S2), including markers previously linked to NSC quiescence (e.g. Mfge8, Ednrb, Ttyh1, Mt3, Ptprz1). Immunofluorescence validation at E17.5 confirmed that Apoe, a top differentially expressed gene enriched in quiescent RGCs cluster, was expressed in Sox2^+^ RGCs and exhibited regional bias: 8.4% of cortical versus 19.7% of LGE Sox2^+^ RGCs were Apoe^+^ (Fig. S9A–E). Nearly 90% of Apoe^+^ cells were Ki67^−^ in both regions, supporting its association with quiescence (Fig. S9E). Furthermore, established adult hippocampal quiescent NSC markers (e.g. Aldoc, Cd9, Clu, Entpd2, Mfge8, Gstm1) [28–31] were similarly enriched in embryonic quiescent RGCs (Fig. S9F), suggesting a conserved molecular signature of quiescence across brain regions and developmental stages.

Together, these results illuminate the dynamic behavior and transcriptional landscape of Vcam1^+^ lineages during embryonic development and reinforce Apoe as a regionalized marker of quiescent NSCs [30,31].

### The developmental trajectory of Vcam1^+^ cells toward the quiescent state enriched in a specific branch

To further analyze the lineage specification of Vcam1^+^ cells, we performed RNA velocity analysis (Fig. 6A). The velocity results exhibited a pattern that the apical dividing cells serve as root which branched distinct pathways, including one leading to the quiescent RGCs. Then we used STREAM [32] to generate developmental cell fate trajectories. Consistently, the pseudotime analysis identified a common pool of progenitor cells that subsequently diverged into three branches (Fig. 6B). Mapping the cluster annotations onto the pseudotime identified that the initial unspecified branch (S0–S1) consisted mostly of apical dividing RGCs. Subsequently, the root cells separated into two branches (S1–S2, S1–S3). Branch S1–S2 showed a similar cell type composition to the initial branch. In contrast, branch S1–S3 contained more cell types, including quiescent RGCs and IPCs that differentiate toward neurons. At the S3 branching point, the cells were bifurcated into two branches. The S3–S4 branch mainly comprised quiescent cells and primed quiescent cells (46% and 21%, respectively), while the S3–S5 branch was mainly occupied by differentiated cells committed to the neuronal fate (91%) (Fig. 6B, C). Next, we projected the sample types onto the pseudotime and found that cells from the cortex and GE exhibited similar developmental trajectories (Fig. S10A). Similarly, cells from E13.5 and E17.5 also exhibit similar developmental trajectories, albeit with some distributional differences. Cells at E13.5 were primarily concentrated in the initial branch (S0–S1), whereas cells at E17.5 were mostly distributed in the subsequent branches (S3–S4, S3–S5) (Fig. S10A). The proportion of cells derived from the Cortex versus the GE was remarkably similar, especially at the critical branch points (S1–S3 and S3–S4) (Fig. S10B). This indicates that the fundamental branching structure of the trajectory is not primarily driven by regional identity. Instead, the major variable we observed was the contribution of cells from different developmental stages across pseudotime. This shift in the temporal composition of cells along the trajectory is, in fact, an inherent and expected feature of a *bona fide* developmental process. It validates that our pseudotime analysis accurately recapitulates a true biological progression over time.

**Figure 6.**
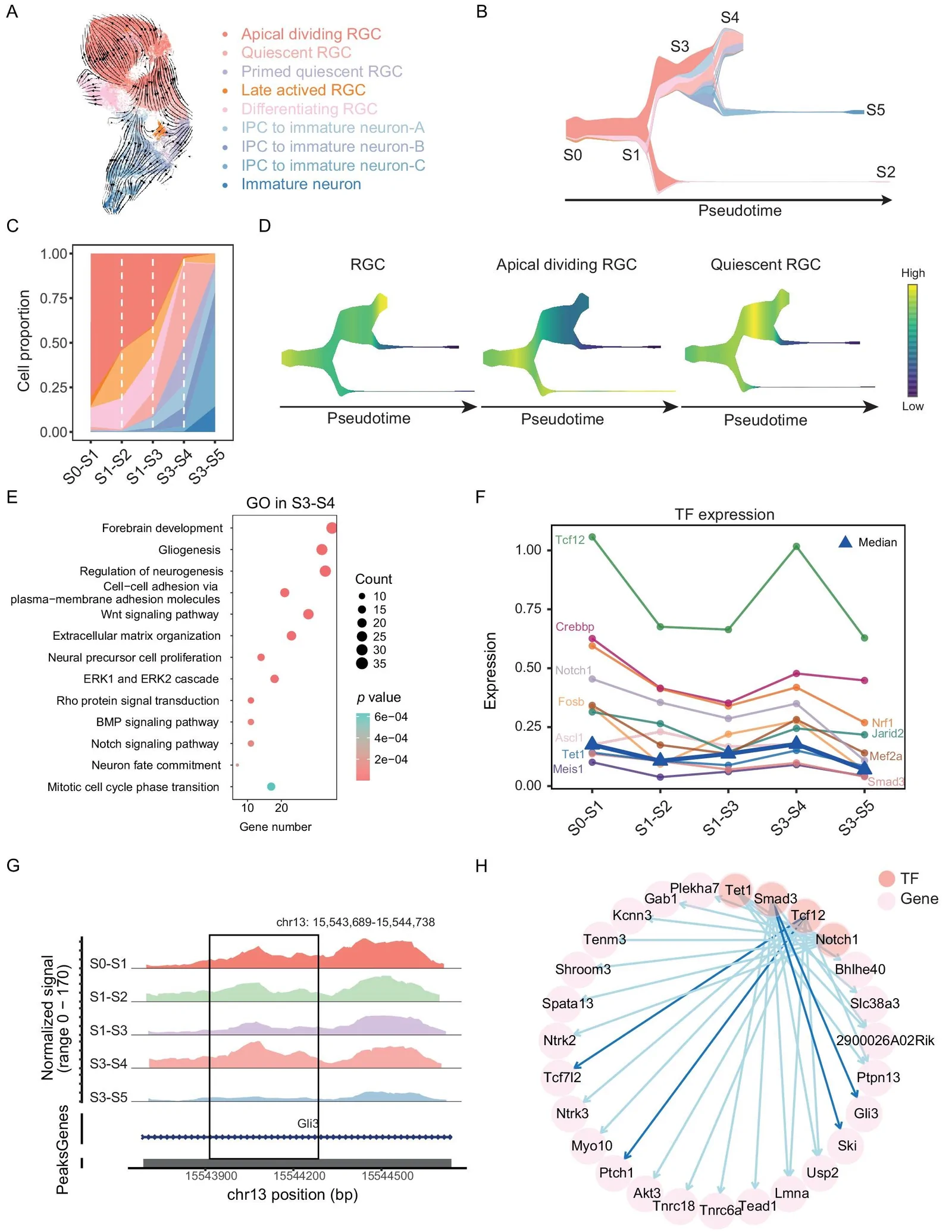
Gene expression and chromatin accessibility dynamics along the pseudo-time trajectory (A) RNA velocity of cells subtypes. (B) Pseudo-time analysis showing developmental trajectories of cells in each cluster from E13.5–E17.5. (C) Line charts showing transition proportion of cell clusters along the pseudo-time trajectory. (D) Expression of gene sets (Table S1) along developmental pseudotime. (E) Gene ontology analysis for branch S3–S4. (F) Expression of 11 predicted TFs in each branch. Box plots show the inter-quartile ranges (boxes), maximum/minimum values (whiskers) and medians (central horizontal lines) (G) Pseudo-bulk accessibility tracks of the Gli3 gene region in each branch. (H) Gene regulatory network showing TF-gene interactions specific in fate determination toward quiescent-state.

We then determined the state of different branches by examining the expression of canonical markers (Fig. S10C). Embryonic RGC markers (Sox2, Vim, Slc1a3) were highly expressed in the initial branch (S0–S1) and persisted in the terminal branch (S3–S4). The proliferation marker Mki67 was also highly expressed in the initial branch, but only concentrated in one distal branch S1–S2, indicating the active self-renewal state enriched branch S1–S2 and the quiescent state enriched branch S3–S4. Furthermore, we analyzed genes that are highly expressed in adult B1 cells relative to astrocytes (Cebrian-Silla *et al.*, 2021, eLife) and found that these genes are significantly enriched in the S3-S4 branch (Fig. S10D). This provides additional evidence that the trajectory from RGCs toward quiescence and subsequently to adult B1 cells is primarily concentrated within the S3–S4 branch.

Next, we examined the expression patterns of cluster-specific gene sets (Table S1) along the pseudotime trajectory, further substantiating the distinct cellular states represented by the three terminal branches (Fig. 6D). Through differential gene expression analysis, we discovered a list of enriched genes for each branch as potential novel markers (Table S3). All branches exhibited unique gene profiles that distinguished them from each other (Fig. S11A).

It is noteworthy that Vcam1 continued to be expressed from the initial branch to the quiescent-state-enriched branch (S3–S4), meanwhile its expression was significantly reduced in the neuronal differentiation branch and active self-renewal branch (S3–S5, S1–S2) (Fig. S10C). This Vcam1 dynamic expression pattern indicated that Vcam1^+^ cells actively participate in neurogenesis during early development. As development progresses, the majority of Vcam1^+^ cells transition into a relatively quiescent state to become seed cells of adult NSCs (S3–S4), while a small portion enters a self-renewing state to maintain the stem cell pool (S1–S2). Additionally, a subset of progeny cells lost Vcam1 expression and differentiated into neurons (S3–S5).

### Transcriptome and chromatin accessibility dynamics during the quiescent state transition

To gain insight into lineage signatures and fate determination mechanism of Vcam1^+^ cells into quiescent state, we further investigated the dynamics of transcriptome and epigenome during the trajectory transition. Quiescent state cells develop from transitional branch S1–S3, and at the S3 branch point, root cells bifurcate to distinct cell fates. Therefore, we focused on the transcriptome and epigenome differences between the two branches: S1–S3 and S3–S4 during the cell fate determination toward a quiescent state. Firstly, we performed a differential gene expression analysis between branches S1–S3 and S3–S4, and a total of 489 differentially expressed genes (DEGs) were identified. Gene ontology (GO) analysis of the S3–S4 enriched genes showed their involvement in forebrain development, specifically participating in neurogenesis and gliogenesis (Fig. 6E). These genes also participate in cell-cell adhesion through plasma-membrane adhesion molecules and extracellular matrix organization, which is consistent with the reported function of Vcam1 [17,18] and in line with our observation of continuous expression of Vcam1 in branch S3–S4 (Fig. S11C). Then, we leveraged the scATAC-seq data to depict the dynamic changes in chromatin accessibility. Differentially expressed peaks (DEPs) between branches S1–S3 and S3–S4 were identified, and their nearest associated genes (DEPs-gene) were inferred using Signac [33]. To integrate the scRNA-Seq and scATAC-Seq profiles, we then screened out the overlapping subset of filtered DEGs and DEPs-genes. The Lisa method was used to forecast the potential upstream transcription factors (TFs) influencing the DEGs [34] based on these overlapping genes. Targets of these TFs were identified by joint analysis with publicly available ChIP-sequencing (ChIP-seq) data from the Cistrome DB database [35].

Based on the above analysis, we screened out 43 filtered genes specific to branch S3–S4 and predicted 15 potential regulatory TFs (Table S4). Coincident with gene expression, the average expression levels of these TFs in branch S3–S4 are higher compared to other branches, especially during the transition from branch S1–S3 to S3–S4 (Fig. 6F). Furthermore, chromatin accessibility of the filtered genes was verified. For instance, the loci of Gli3 showed quiescent-state specific open conformation with significant differences in the transition process (S1–S3 to S3–S4), as well as between distal branches (S3–S4 and S3–S5) with distinct cell fates (Fig. 6G). Further, the quiescent-state specific genes and TFs were subjected to network analysis which showed that Tet1, Smad3, Tcf12 and Notch1 might be key regulatory factors in determining the fate of quiescence (Fig. 6H). These TFs are all of specifically high expression in the quiescent state branch (Fig. S11) and have been reported to be involved in the fate determination of progenitor cells during neural development [36–39]. Among these TFs, we found Tcf12 is consistently expressed in the neurogenic region from embryonic to adult stages, and its postnatal expression parallels ongoing adult neurogenesis [38]. The similar expression pattern of Tcf12 and Vcam1 suggests that Tcf12 may also serve as a marker for adult NSCs and their seed cells in embryonic stage. By leveraging network analysis, we found quiescent-state specific genes like Gli3 [40,41] and Ski [42] were downstream of the same TF, Smad3, suggesting that Smad3 may regulate quiescent state specification through TGF-β signaling and Shh signaling (Fig. S11C, D). Meanwhile, we identified genes like Ptch1 [43] and Tcf7l2 [44] that were regulated by Tcf12, indicating the potential involvement of Shh signaling and Wnt/β-catenin signaling in Tcf12-modulated quiescent state specification (Fig. S11C, D). Together, these results suggest a complicated network of quiescent state determination involving TGF-β signaling, Shh signaling and Wnt/β-catenin signaling. To sum up, our analysis revealed lineage signatures and key regulatory factors for quiescent Vcam1^+^ RGC and delineate the relevant regulatory pathways by establishing TF-gene networks.

### Smad3 participates in determining quiescent cell fate through TGF-β pathway

Members of the Smad family of signal transduction molecules are components of a critical intracellular pathway that transmits TGF-β signals from the cell surface into the nucleus. Following stimulation by TGF-β, Smad3 become phosphorylated at carboxyl terminal serine residues (Ser465 and 467 on Smad2; Ser423 and 425 on Smad3) by TGF-β Receptor I. Phosphorylated Smad 2/3 can form complex with Smad4 and translocate to the nucleus to regulate target genes expression [45–47].

In the pseudotime trajectory, genes associated with TGF-β (especially Smad3) were enriched in the branch S3-S4 (quiescent state) (Fig. S12A). Similar to Vcam1, we observed higher cell proportion and expression level of *Smad3* in both apical dividing RGC cluster and quiescent RGC clusters compared with other cell types (Fig. S12B). However, the expression of Smad3 was not very exclusive to the quiescent cluster. Immunohistochemical staining at E17.5 brain revealed Smad3 protein in Sox2^+^ RGCs, though the signal was predominantly cytoplasmic and did not show a clearl difference between Ki67^+^ and Ki67^-^ cells (Fig. S12C). This pattern is consistent with previous reports that Smad3 subcellular localization is dynamic and context-dependent, with cytoplasmic retention observed even in its activated state [46,48,49]. To further corroborate its expression in VZ, we examined *in situ* hybridization (ISH) data from the Allen Brain Atlas [http://developingmouse.brain-map.org/gene/show/16897], which confirmed *Smad3* mRNA enrichment in the embryonic VZ (Fig. S12D). Notably, ISH data across developmental stages revealed that Smad3 signal was strongest at early fate-determination stages (E11.5, E13.5) and dramatically reduced by E15.5 and E18.5. The upregulation of Smad3 along the trajectory from S1–S3 to S3–S4 suggests that Smad3 contributes to the acquisition of quiescence rather than merely maintaining the quiescent state. Unlike marker genes with relatively static expression patterns, dynamically regulated factors such as Smad3 are likely to be involved in fate determination and thus of greater functional significance in the developmental transition toward quiescence.

To functionally characterize the TGF-β/Smad3 signaling pathway, we constructed an overexpression vector encoding Smad3 mutant (Smad3-Mut) by substituting two C-terminal serine residues (S423 and S425) with alanine, which abolishes TGF-β-induced phosphorylation, A vector overexpressing wild-type Smad3 (Smad3-WT) was built for evaluating effects of phosphorylation on Smad3 functions.

We first introduced these constructs into cortical RGCs via *in utero* electroporation (IUE) at E14.5 and analyzed the brains at E16.5 (Fig. 7A–D). Overexpression of Smad3-WT significantly increased the proportion of Sox2^+^ RGCs, whereas Smad3-Mut reduced it, indicating that Smad3 phosphorylation regulates the size of the RGC pool (Fig. 7B). Smad3-WT also decreased the proportion of Ki67^+^Sox2^+^GFP^+^ cells, suggesting a reduction in RGC proliferation. Co-electroporation of Smad3-Mut with Smad3-WT partially rescued this phenotype, supporting a competitive mechanism (Fig. 7C). Furthermore, Vcam1 signal intensity was significantly higher in the RGCs overexpressing Smad3-WT group than in overexpressing Smad3-Mut group, consistent with a role for phosphorylated Smad3 in promoting a quiescent, Vcam1-high state (Fig. 7D).

**Figure 7.**
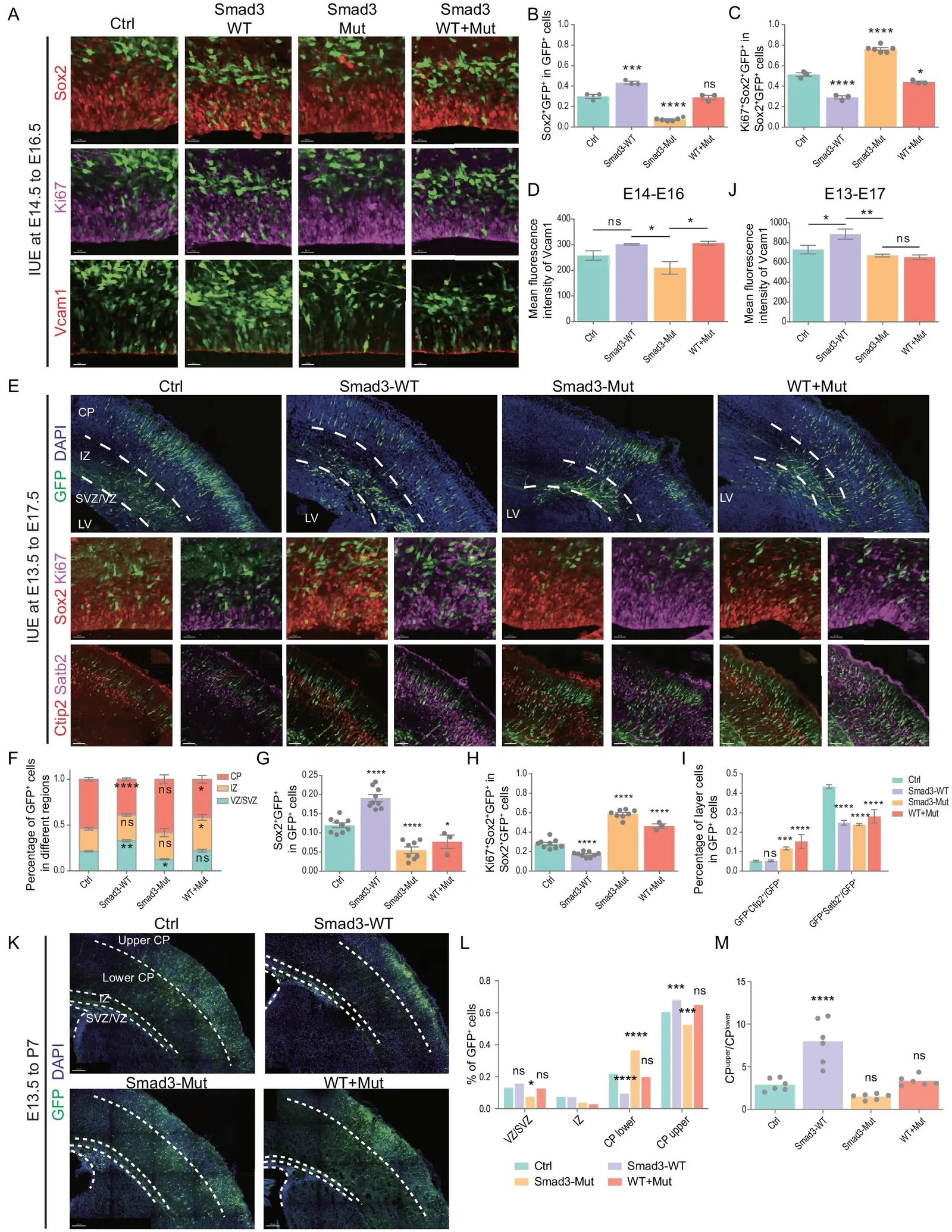
Function analysis of Smad3 by IUE. (A) Immunofluorescent staining of coronal sections of the E16.5 mouse cortex 2 day post‐IUE. Sox2, Ki67, Vcam1 were co‐stained with GFP. Scale bars: 20 μm. (B) Quantification of the relative ratio of Sox2 and GFP double‐positive cells versus GFP‐positive cells. (C) Quantification of the relative ratio of Ki67, Sox2, GFP positive cells versus Sox2, GFP double-positive cells. (D) Statistical analysis of Vcam1 intensity among different groups. Data were presented as mean ± SEM. Statistical significance was tested by two‐way ANOVA with multiple comparisons. *P < 0.05. (E) Immunofluorescent staining of coronal sections of the E17.5 mouse cortex 4 days post‐IUE (top). Sox2 and Ki67 were co‐stained with GFP (middle top). Ctip2 and Satb2 were co‐stained with GFP (middle bottom). Scale bars: 100 μm (GFP distribution), 50 μm (Ctip2/Satb2 staining), 20 μm (Sox2/Ki67 staining). (F) Quantification of GFP‐positive cells in VZ/SVZ, IZ, and CP at E17.5 (4 days post-IUE). (G) Quantification of the relative ratio of Sox2 and GFP double‐positive cells versus GFP‐positive cells at E17.5 (4 days post-IUE). (H) Quantification of the relative ratio of Ki67, Sox2, GFP positive cells versus Sox2, GFP double-positive cells at E17.5 (4 days post-IUE). (I) Quantification of the relative ratio of Ctip2/Satb2 and GFP double‐positive cells versus GFP‐positive cells (4 days post-IUE). (J) Statistic analysis of Vcam1 intensity among different groups (4 days post-IUE). Data were presented as mean ± SEM. Statistical significance was tested by two‐way ANOVA with multiple comparisons. *P < 0.05, **P < 0.01. (K) Immunofluorescent staining of coronal sections of the P7 mouse cortex. Scale bars: 150 μm. (L) Quantification of GFP‐positive cells in VZ/SVZ, IZ, and CP at P7. Data were presented as mean ± SEM. Statistical significance was tested by two‐way ANOVA. ***P < 0.001. ****P < 0.0001. (M) Quantification of the relative ratio of GFP positive cells in upper CP versus those in lower CP. Data were presented as mean ± SEM. Statistical significance was tested by one‐way ANOVA. ****P < 0.0001.

To assess the impact of Smad3 on neurogenesis, we electroporated Smad3-Mut and Smad3-WT at E13.5 and analyzed brains at E17.5 (Fig. 7E–J). Smad3-WT overexpression increased GFP^+^ cell retention in the VZ/SVZ and reduced their migration to the cortical plate (CP), whereas Smad3-Mut led to premature depletion of VZ/SVZ-resident cells (Fig. 7E, F). Correspondingly, Smad3-WT increased the proportion of Sox2^+^ cells and decreased proliferation (Ki67^+^), while Smad3-Mut enhanced proliferation and promoted deeper-layer neuron differentiation, reflected by an increased proportion of Ctip2^+^ deep-layer neurons and a reduced number of Satb2^+^ upper-layer neurons (Fig. 7G–I). Vcam1 intensity was elevated in the Smad3-WT group, further supporting its role in sustaining a quiescent progenitor state (Fig. 7J).

In a long-term experiment, electroporation at E13.5 and analysis at P7 revealed that Smad3-WT overexpression maintained GFP^+^ cells in the VZ/SVZ and increased GFP^+^ upper-layer neurons, consistent with delayed differentiation and NSC pool preservation (Fig. 7K, L). In contrast, Smad3-Mut reduced VZ/SVZ retention of GFP^+^ cells and GFP^+^ upper-layer neurons while increasing GFP^+^ deep-layer neurons, confirming that loss of Smad3 phosphorylation drives premature differentiation and progenitor pool depletion (Fig. 7K, L).

## DISCUSSION AND CONCLUSION

During early neural development, majority of NSCs display morphological homogeneity yet undergo divergent fate specification. The mechanisms governing NSC fate determination and differentiation potential remain incompletely understood. By systematically characterizing the cellular properties, lineage trajectories, and behavioral dynamics of the Vcam1^+^ NSC subpopulation, our study elucidates one embryonic origin of adult neural progenitors in the mouse V–SVZ and identifies molecular pathways underlying NSC fate commitment and maintenance.

### Lineage tracing reveals spatiotemporal dynamics of Vcam1^+^ NSC pools

Using dual-fluorescent reporter and inducible CreER^T2^ systems, we established a genetic platform for real-time visualization and lineage tracing of Vcam1^+^ RGCs. Unlike existing models such as *Hopx-CreER^T2^* [16], which labels hippocampal neural progenitors, or *Nestin-CreER^T2^*, which broadly targets pan-neural progenitors, *Vcam1-CreER^T2^* specifically enables more specifically tracing of embryonic origins of V–SVZ adult NSCs. Acute staining on the primary neural cells and single-cell transcriptomic comparison confirmed that Vcam1 exhibits higher specificity for quiescent RGCs than other RGC markers (e.g. Nestin, Sox2 and Pax6) particularly during late embryogenesis (Figs 1, S2, S13).

Long-term clonal analysis revealed that quiescent Vcam1^+^ cells emerge as early as E10.5 and are dynamically maintained throughout embryogenesis. While early-labeled clones (e.g. E10.5 labeling) contained quiescent single-R clones, the proportion of quiescent RGCs increased over developmental progress, indicating continuous replenishment from self-renewing Vcam1^+^ RGCs (Figs 3, S5). Critically, we uncovered a developmental-temporal stratification of NSC fate: early-labeled Vcam1^+^ RGCs (E13.5) differentiate into neuronal and glial progenies, with half of them contributing to B1 cells by early postnatal stages, whereas late-labeled cells (E17.5) contributed significantly to the adult NSC pool, particularly in the GE (Fig. 4). These findings suggest that the adult quiescent NSC pool is not established solely during early embryogenesis but is dynamically replenished through a late-embryonic wave of Vcam1^+^ RGCs. Clone size analysis further indicated a developmental decline in proliferative activity among Vcam1^+^ RGCs, consistent with a progressive shift toward quiescence (Fig. 3B, C).

Regional comparisons revealed similar clonal compositions in the cortex and GE, though the GE exhibited a higher proportion of quiescent and self-renewing clones (Figs 3, S5). This regional bias aligns with the preferential preservation of adult NSCs in the striatal V–SVZ and highlights the role of localized niche factors in shaping NSC dynamics.

Our integrated analysis enables the construction of a comprehensive lineage progression model for Vcam1^+^ seed cells (Fig. 8A). This roadmap delineates how quiescent Vcam1^+^ cells are established beginning at E10.5 and persist throughout embryogenesis. It further illustrates the dynamic replenishment mechanism wherein self-renewing Vcam1^+^ RGCs continuously contribute to the quiescent pool during mid- to late-embryonic neurogenic stages. The model highlights the regional disparity between cortical and GE-derived lineages, with the latter exhibiting enhanced retention of quiescent NSCs. Most importantly, it captures the temporally regulated embryonic process wherein early stage-originated quiescent RGC population are progressively supplied by late stage-originated populations, ultimately establishing the adult NSC reservoir.

**Figure 8.**
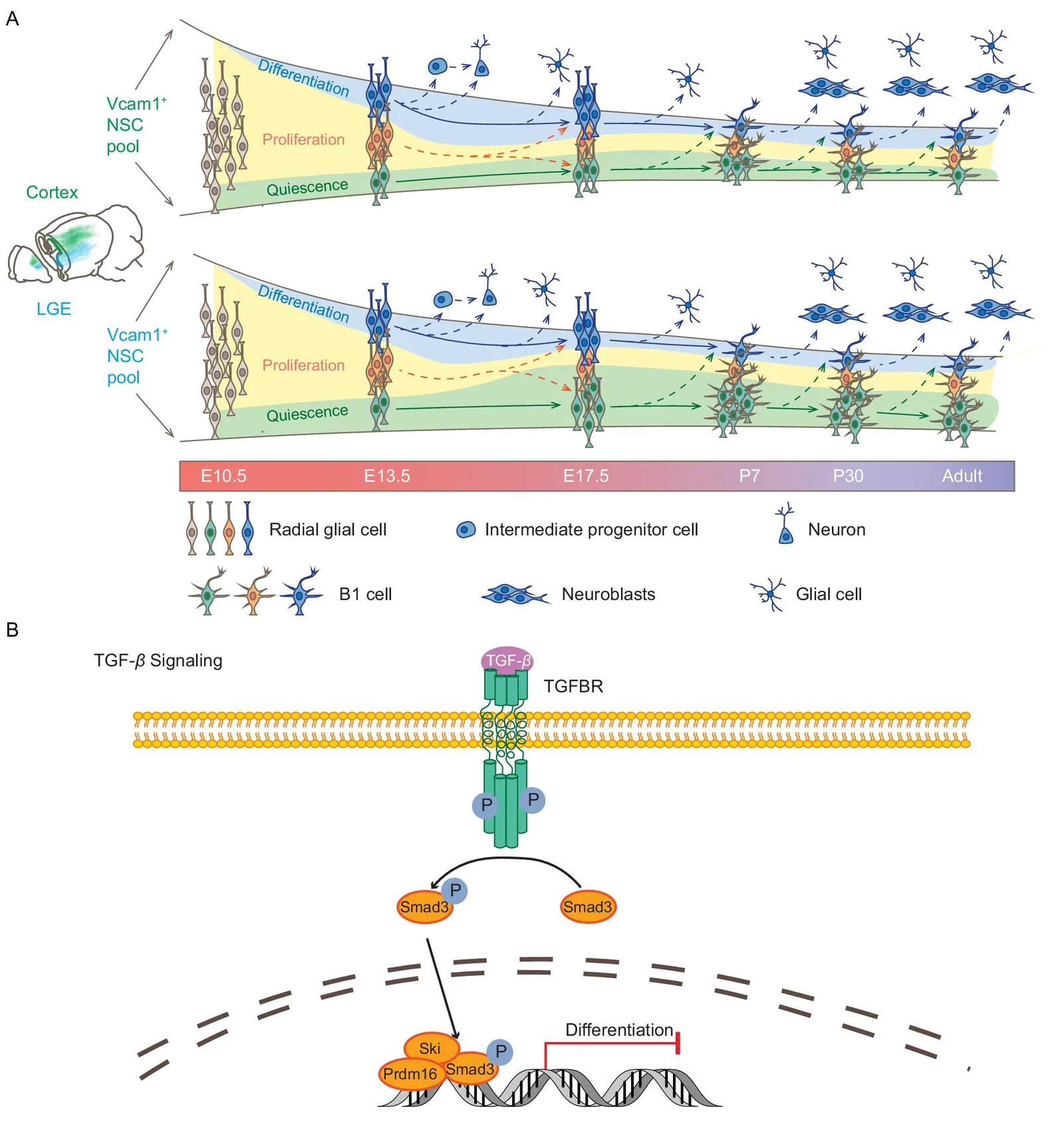
Summary of Vcam1^+^ NSC pool dynamics during development and the mechanism underlying this process. (A) Summary of the developmental dynamics of Vcam1^+^ NSCs. As the embryo develops, the Vcam1^+^ stem cell pool gradually decreases. Quiescent Vcam1^+^ cells appear at the beginning of neurogenesis and are maintained at a certain proportion throughout the embryonic stage. In addition, new RGCs generated by self-renewal will also replenish into the quiescent RGC pool to expand the number of quiescent RGCs during mid-embryonic development. Quiescent Vcam1^+^ RGCs as seed cells of adult NSCs will be activated and differentiate after birth in the order of their generation in embryonic stage. As a result, only quiescent Vcam1^+^ RGCs at late embryonic stage will be preserved until P30 or even adulthood as adult NSCs. Besides, compared to the cortex, a higher proportion of quiescent cells preserved in the late stage of embryonic development in the GE, explaining the higher number of adult NSCs in the striatal side of V–SVZ. (B) Summary of molecular regulators and regulating pathway of Vcam1^+^ RGCs across embryonic development.

### Single-cell multiomics delineates a quiescence-enriched trajectory

Integrative scRNA-seq and scATAC-seq profiling of Vcam1^+^ lineages uncovered a heterogeneous yet structured developmental continuum. Pseudotime reconstruction revealed a dedicated trajectory branch (S3–S4) enriched for quiescent RGCs, representing potential seed cells for adult NSCs (Fig. 6). The gene signature specific to this branch-specific included known quiescence-associated factors such as lysosomal genes, consistent with transcriptional profiles reported in hippocampal quiescent NSCs [16], and was further validated in our dataset (Fig. S14).

Notably, while cortical NSC development has been previously described as following a ‘sequential model,’ Vcam1-enriched approach revealed a coexisting ‘set-aside’ subpopulation that deviates early toward quiescence. This subpopulation is conserved across cortex and GE but differs in abundance, suggesting that regional disparities in the size of adult NSC pools arise from quantitative rather than qualitative differences in lineage specification.

Regulatory network analysis identified key transcription factors—including Tet1, Smad3, Tcf12, and Notch1—associated with the quiescent state. Functional validation by overexpression of phospho-deficient Smad3 demonstrated that TGF-β/Smad3 signaling promotes Vcam1 expression and suppresses proliferation, thereby directing RGCs toward quiescence (Fig. 7). These findings position pSmad3 as a pivotal regulator of quiescence entry.

After the majority of embryonic neurogenesis ceased around E17.5, RGCs are thought to switch to initiate astrogliogenesis. It remains an interesting question in future study whether some of the quiescent RGCs will give rise to adult astrocytes afterward. A series of single-cell data analysis from later perinatal stage, postnatal stage to adult would be required to fully resolve this.

Collectively, our study repositions Vcam1 not merely as a marker but as a functional entry point to dissect the developmental logic of NSC fate divergence. We demonstrate that embryonic Vcam1^+^ NSC population is dynamic, and quiescent Vcam1^+^ NSCs population forms early and is replenished by Vcam1 persistent NSCs across development. TGF-β/Smad3-mediated pathway regulates this progress. These insights establish a foundational framework for understanding how developmental timing, regional identity, and molecular signaling pathways collectively orchestrate the establishment of the adult NSC reservoir.

### Limitations of study

Our study reveals an embryonic origin of adult NSCs in the mouse V–SVZ. While these findings define one developmental trajectory contributing to the adult V–SVZ, they do not exclude the possibility of additional embryonic origins for distinct neural progenitor populations in the mammalian adult brain. Consistent with this view, a study by Berg and colleagues demonstrated that Hopx-expressing neural progenitor cells in the embryonic dentate gyrus contribute to lifelong neurogenesis in the adult SGZ, supporting a continuous model of developmental origin for adult neurogenesis [16]. These observations collectively highlight the heterogeneity of adult NSCs and the likelihood that multiple embryonic progenitor pools contribute to the maintenance of neurogenesis in distinct adult niches.

Since the *Vcam1CreER^T2^; Ai9* mice only have monochromatic fluorescence for lineage tracing (tdT), we believe that future integration of multicolor lineage tracing methods, such as the StarTrack method or dual-label mosaic analysis (MADM), will facilitate more precise clonal analysis and deeper validation.

Due to the requirements of sample amount for single-cell multi-omics sequencing, it is challenging to collect a sufficient number of Vcam1^+^ RGCs at E13.5 cortex and GE respectively by FACS for scRNA-ATAC sequencing. Future advances in the scRNA-ATAC techniques would expand analysis on molecular regulatory network of Vcam1^+^ RGCs at early neurogenesis stage from both transcriptomic level and epigenetic level.

While this study focuses on the embryonic origins of adult NSCs and the critical period of seed cell fate determination, the newly generated *Vcam1-CreER^T2^; Ai9* mouse line provides a powerful tool for future studies on the Vcam1⁺ subpopulation within the adult neurogenic niche. Comparative lineage analysis of the Vcam1-lineage versus other classical NSC lineages in the adult stage will be critical to illuminate how heterogeneity of adult NSCs contributes to the homeostasis of adult neurogenesis and neural repair upon injury and disease. These important questions, along with the potential differences between cortical and subcortical progenitors, remain fascinating areas for dedicated investigation in future studies.

## MATERIALS AND METHODS

### Ethics statement

All animal experiments were reviewed and approved by the IACUC of Tongji University (ID: TJAB09024101). All applicable institutional and national guidelines for the care and use of animals were strictly followed. Both sexes were used across embryonic, postnatal, and adult stages without sex-stratified analyses. The *Vcam1* reporter and *Vcam1-CreER^T2^* knockin alleles were generated via CRISPR/Cas9 and ES-cell targeting, respectively, confirmed by PCR, sequencing, and Southern blot.

### Tamoxifen, BrdU and EdU administration

Tamoxifen (20 mg/mL in corn oil/10% ethanol) was intraperitoneally injected into pregnant dams at indicated embryonic days. For embryonic labeling followed by postnatal analysis, embryos were recovered by C-section at E19.5 and fostered. For cell-cycle exit, BrdU (50 mg/kg) was injected 24 h before sacrifice; for slow-dividing cells, EdU (5 mg/kg) was given four times at 2-h intervals at E11.5.

### Immunohistochemistry and wholemount immunostaining

Embryonic heads or isolated brains were fixed in 4% PFA, cryoprotected in sucrose, and sectioned (45–60 μm). Postnatal mice were transcardially perfused. Sections were blocked, incubated with primary antibodies at 4°C overnight, followed by fluorophore-conjugated secondaries. SVZ wholemounts were dissected and stained with primary antibodies for 48 h at 4°C. For acute cell staining, dissociated cortical/GE cells were plated on PLL-coated coverslips and fixed 3 h later.

### Flow cytometry

Dissociated cells were acquired on a BD LSRFortessa (488/561/640 nm lasers). At least 50 000 events were collected, and data were analyzed with FlowJo. Positive gates were set against isotype controls.

### Confocal microscopy and image processing

Z-stack images (1.5 μm step) were acquired on Zeiss LSM 880 or Olympus SpinSR. 3D reconstruction used ImageJ Fiji and Imaris 10. Cell–cell distances and NND analyses were performed on 3D datasets.

### Clonal analysis

Sparse labeling was achieved by tamoxifen dose titration (<10 clones/hemisphere). Serial coronal sections were stained for tdT, and clones were imaged entirely. Cell types were assessed by marker expression (Nestin/Sox2 for progenitors; Tbr2/Ascl1 for IPCs; GFAP for B1 cells) and morphology. Clone origin (cortical vs. LGE) was assigned based on migration path (radial vs. tangential). Radial clusters were scanned with a 200 μm (width) × 600–1200 μm (length) cuboid; tangential clusters with 200 μm diameter × 1000 μm cylinders. Results were compared to 100 × random simulations.

### *In utero* electroporation (IUE)

Pregnant dams were anesthetized with isoflurane. Plasmid DNA (2–3 μg/μL, mixed with Fast Green) was injected into lateral ventricles of embryos, followed by electroporation using an ECM830 system.

### Single-cell multiomics

tdT⁺ cells from Vcam1-reporter cortex/LGE at E13.5 and E17.5 were FACS-sorted. scRNA-seq and scATAC-seq libraries were prepared (10× Genomics) and sequenced on NovaSeq 6000 (150-bp paired-end). Raw data were processed with Cell Ranger and analyzed using Seurat (scRNA) and Signac (scATAC). Cells with >200 genes (scRNA) or 1000–20 000 fragments (scATAC) were included. Mitochondrial-rich clusters and non-neural cell types (microglia, blood, etc.) were excluded. Trajectory inference used the STREAM pipeline. Differential expression genes (DEGs) and peaks (DEPs) were identified; upstream TFs were predicted via LISA and Cistrome DB.

### Quantification and statistical analysis

Data are mean ± SEM. Statistical significance was assessed by two-tailed Student’s *t*-test or one-way ANOVA with Dunnett’s posttest (Prism). *P* < 0.05 was considered significant. *n* values are detailed in figure legends.

## Supplementary Material

nwag516_Supplemental_Files

## Data Availability

The raw sequence data reported in this paper have been deposited in the Genome Sequence Archive (Genomics, Proteomics & Bioinformatics 2021) in National Genomics Data Center (Nucleic Acids Res 2022), China National Center for Bioinformation/Beijing Institute of Genomics, Chinese Academy of Sciences (GSA: CRA015800) that are publicly accessible at https://ngdc.cncb.ac.cn/gsa. The source code is available in the Zenodo repository at the following link: https://zenodo.org/records/10902002. Additionally, other processed data can be accessed from the Zenodo repositories at the following link: https://zenodo.org/records/10902117.
