# Supplementary material for: Integrative analysis of the embryonic origin of adult neural stem cells in forebrain subventricular zones
Source: Natl Sci Rev. 2026 Aug 29;13(18):nwag516. doi: 10.1093/nsr/nwag516 (PMC13629234; doi:10.1093/nsr/nwag516)
Supplement: Supplementary file 1 [file nwag516_supplemental_files.zip › supplementary files/figs1.170.wuhuan.0717.pdf]

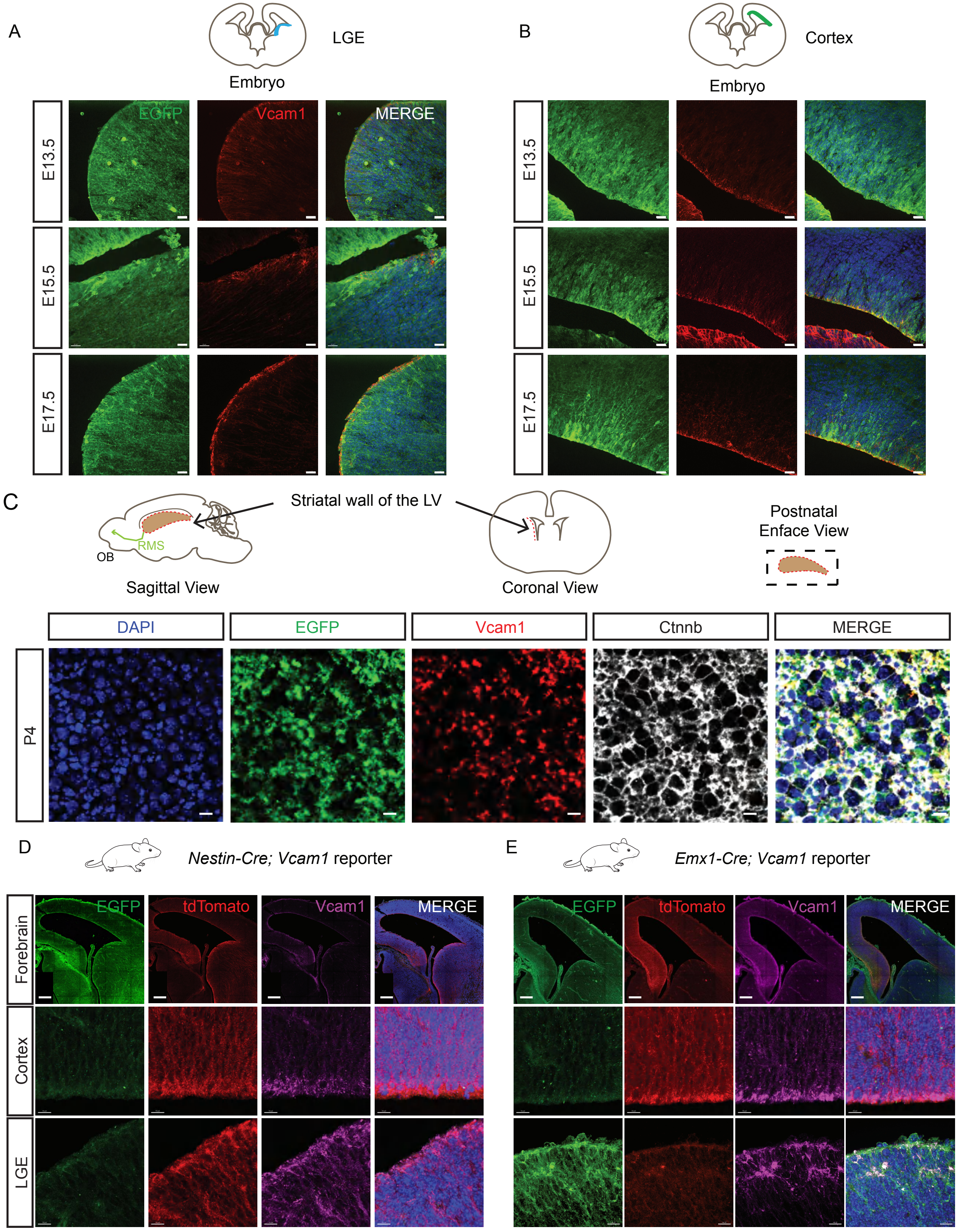
A
LGE
Embryo
EGFP
Vcam1
MERGE
E13.5
E15.5
E17.5
B
Cortex
Embryo
C
Striatal wall of the LV
OB
RMS
Sagittal View
Coronal View
Postnatal
Enface View
DAPI
EGFP
Vcam1
Ctnnb
MERGE
P4
D
Nestin-Cre; Vcam1 reporter
E
Emx1-Cre; Vcam1 reporter
EGFP
tdTomato
Vcam1
MERGE
Forebrain
Cortex
LGE
