# Supplementary material for: Integrative analysis of the embryonic origin of adult neural stem cells in forebrain subventricular zones
Source: Natl Sci Rev. 2026 Aug 29;13(18):nwag516. doi: 10.1093/nsr/nwag516 (PMC13629234; doi:10.1093/nsr/nwag516)
Supplement: Supplementary file 1 [file nwag516_supplemental_files.zip › supplementary files/figs10.170.wuhuan.0717.pdf]

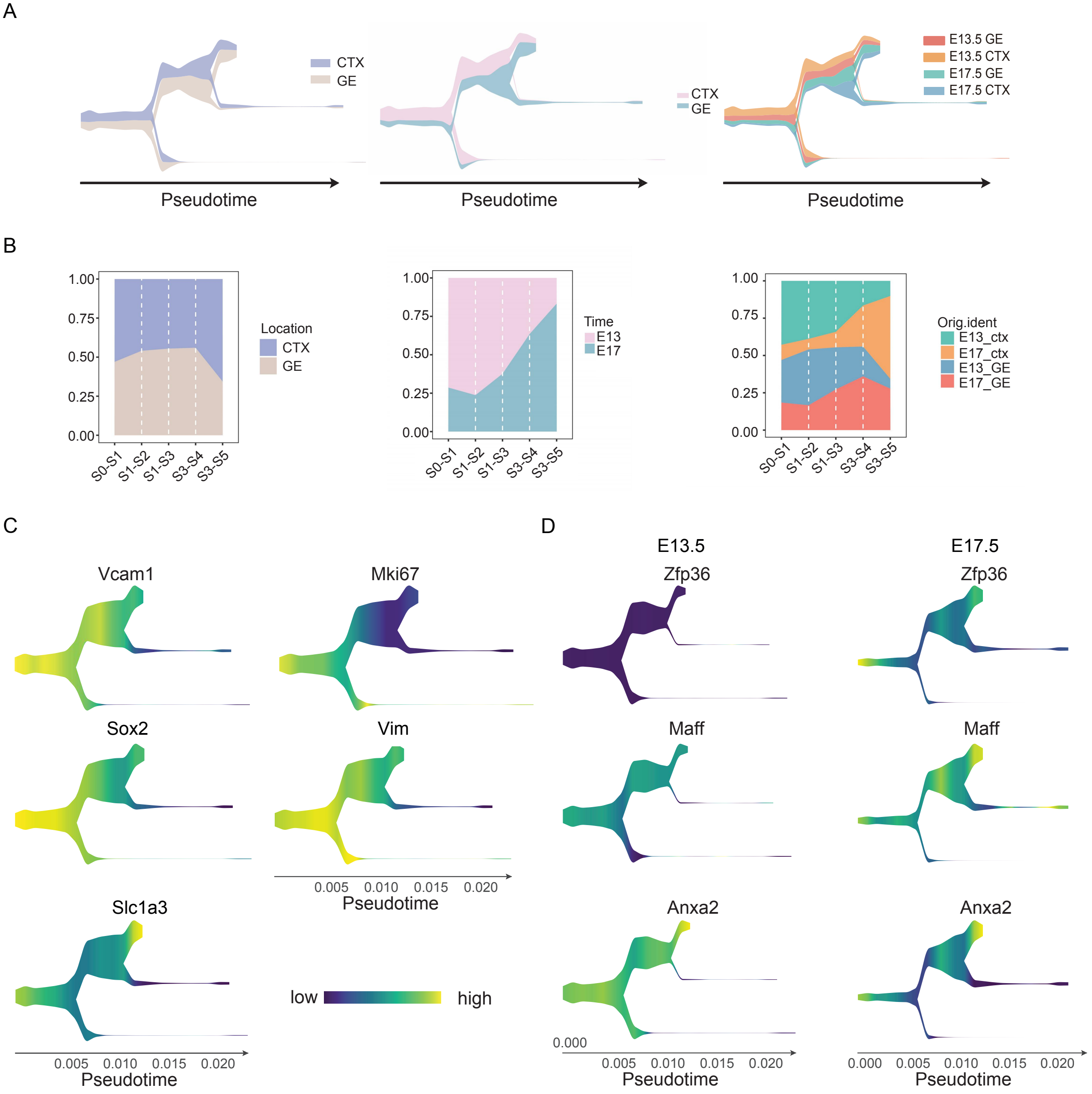
A
CTX
GE
Pseudotime
CTX
GE
Pseudotime
E13.5 GE
E13.5 CTX
E17.5 GE
E17.5 CTX
Pseudotime
B
1.00
0.75
0.50
0.25
0.00
S0-S1
S1-S2
S1-S3
S3-S4
S3-S5
Location
CTX
GE
Time
E13
E17
Orig.ident
E13_ctx
E17_ctx
E13_GE
E17_GE
C
Vcam1
Mki67
Sox2
Vim
Slc1a3
low
high
0.005 0.010 0.015 0.020
Pseudotime
D
E13.5
E17.5
Zfp36
Maff
Anxa2
0.000
Pseudotime
