# Supplementary material for: Integrative analysis of the embryonic origin of adult neural stem cells in forebrain subventricular zones
Source: Natl Sci Rev. 2026 Aug 29;13(18):nwag516. doi: 10.1093/nsr/nwag516 (PMC13629234; doi:10.1093/nsr/nwag516)
Supplement: Supplementary file 1 [file nwag516_supplemental_files.zip › supplementary files/figs11.170.wuhuan.0717.pdf]

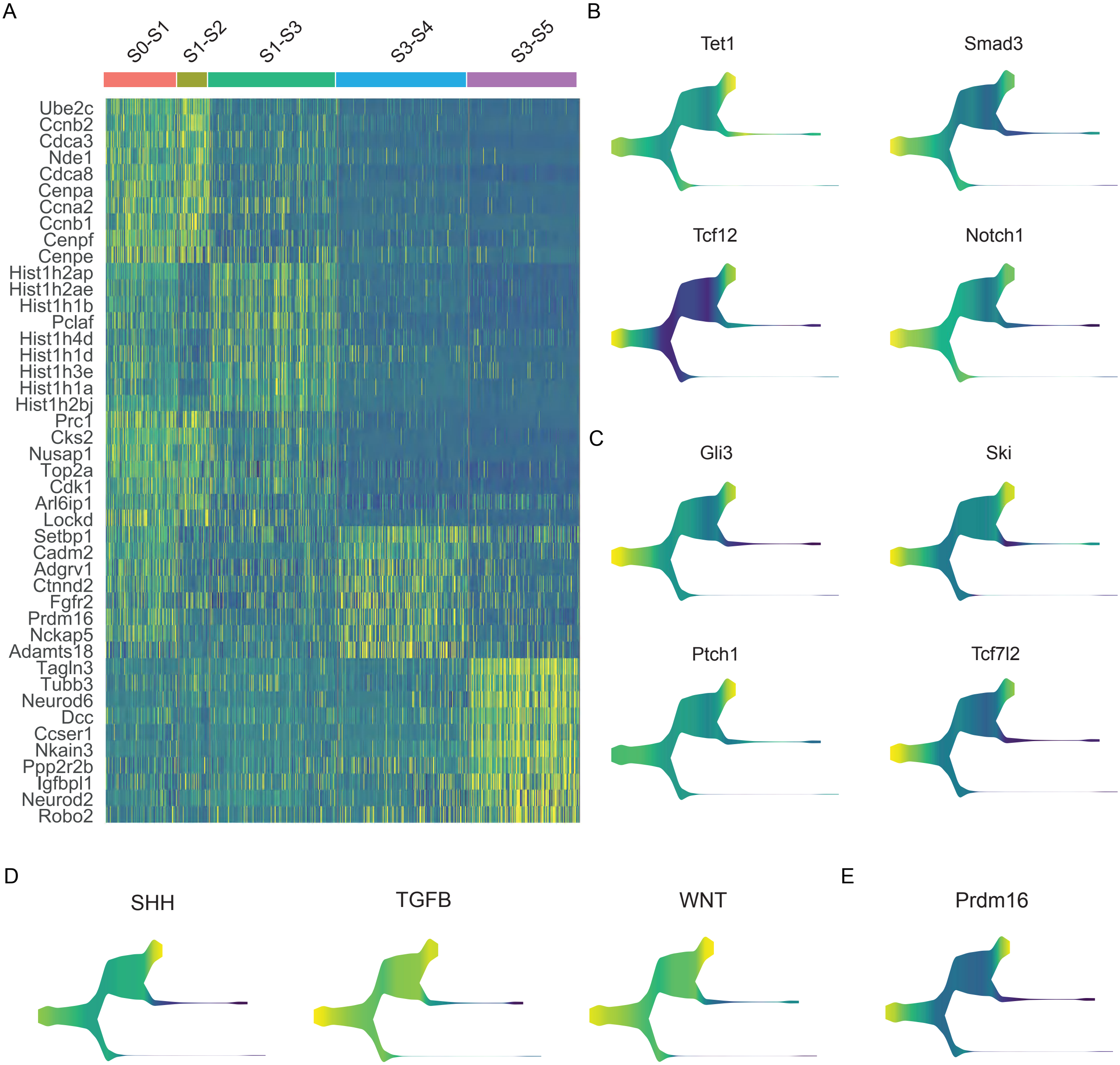
A
S0-S1
S1-S2
S1-S3
S3-S4
S3-S5
Ube2c
Ccnb2
Cdca3
Nde1
Cdca8
Cenpa
Ccna2
Ccnb1
Cenpf
Cenpe
Hist1h2ap
Hist1h2ae
Hist1h1b
Pclaf
Hist1h4d
Hist1h1d
Hist1h3e
Hist1h1a
Hist1h2bj
Prc1
Cks2
Nusap1
Top2a
Cdk1
Arl6ip1
Lockd
Setbp1
Cadm2
Adgrv1
Ctnnd2
Fgfr2
Prdm16
Nckap5
Adamts18
Tagln3
Tubb3
Neurod6
Dcc
Ccser1
Nkain3
Ppp2r2b
Igfbpl1
Neurod2
Robo2
B
Tet1
Smad3
Tcf12
Notch1
C
Gli3
Ski
Ptch1
Tcf7l2
D
SHH
TGFB
WNT
E
Prdm16
