# Supplementary material for: Integrative analysis of the embryonic origin of adult neural stem cells in forebrain subventricular zones
Source: Natl Sci Rev. 2026 Aug 29;13(18):nwag516. doi: 10.1093/nsr/nwag516 (PMC13629234; doi:10.1093/nsr/nwag516)
Supplement: Supplementary file 1 [file nwag516_supplemental_files.zip › supplementary files/figs13.170.wuhuan.0717.pdf]

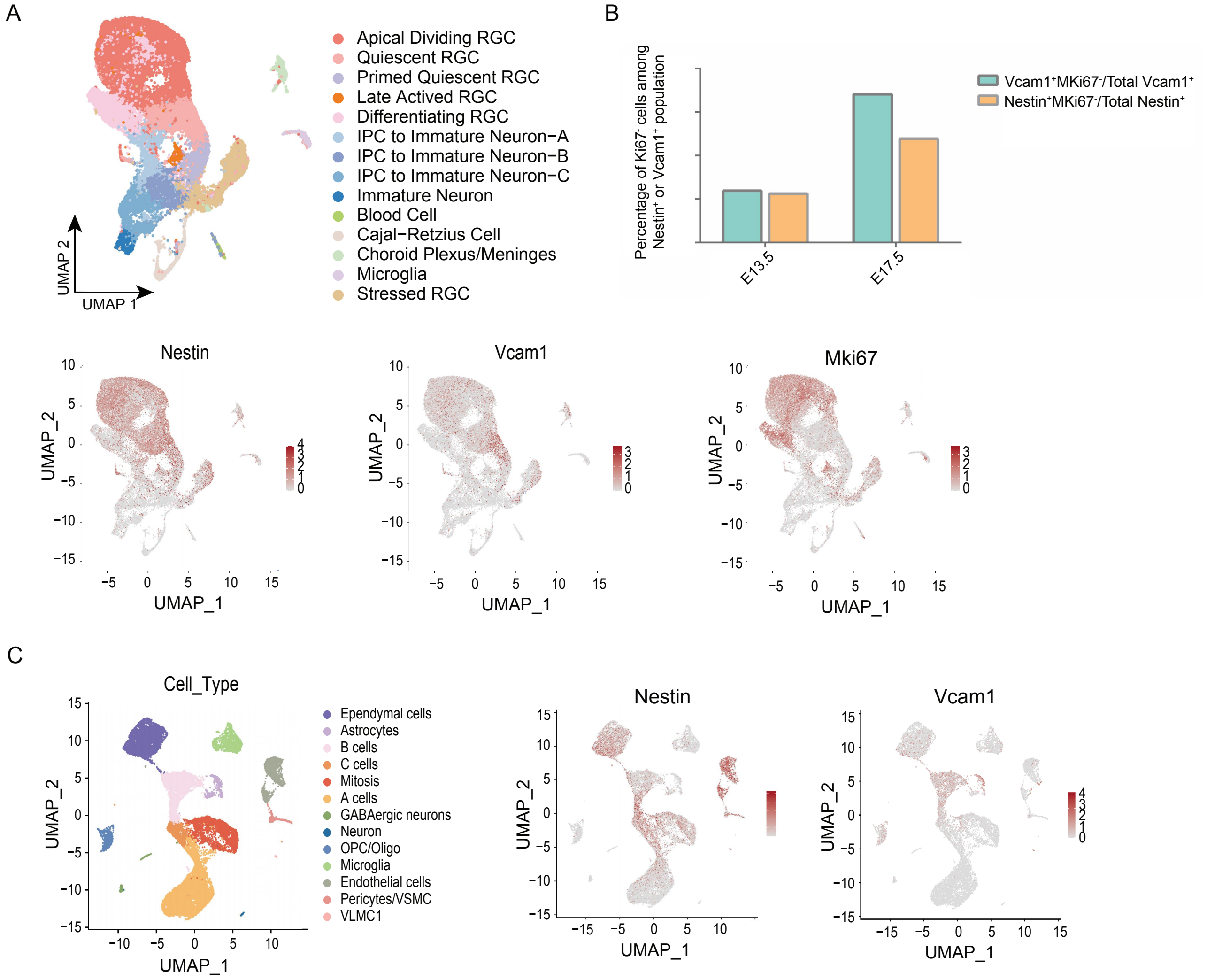

A
Apical Dividing RGC
Quiescent RGC
Primed Quiescent RGC
Late Actived RGC
Differentiating RGC
IPC to Immature Neuron−A
IPC to Immature Neuron−B
IPC to Immature Neuron−C
Immature Neuron
Blood Cell
Cajal−Retzius Cell
Choroid Plexus/Meninges
Microglia
Stressed RGC
UMAP 2
UMAP 1
B
Percentage of Ki67- cells among Nestin+ or Vcam1+ population
Vcam1+MKi67-/Total Vcam1+
Nestin+MKi67-/Total Nestin+
E13.5
E17.5
Nestin
Vcam1
Mki67
UMAP_2
UMAP_1
C
Cell_Type
Ependymal cells
Astrocytes
B cells
C cells
Mitosis
A cells
GABAergic neurons
Neuron
OPC/Oligo
Microglia
Endothelial cells
Pericytes/VSMC
VLMC1
Nestin
Vcam1
UMAP_2
UMAP_1
