# Supplementary material for: Integrative analysis of the embryonic origin of adult neural stem cells in forebrain subventricular zones
Source: Natl Sci Rev. 2026 Aug 29;13(18):nwag516. doi: 10.1093/nsr/nwag516 (PMC13629234; doi:10.1093/nsr/nwag516)
Supplement: Supplementary file 1 [file nwag516_supplemental_files.zip › supplementary files/figs14.170.wuhuan.0717.pdf]

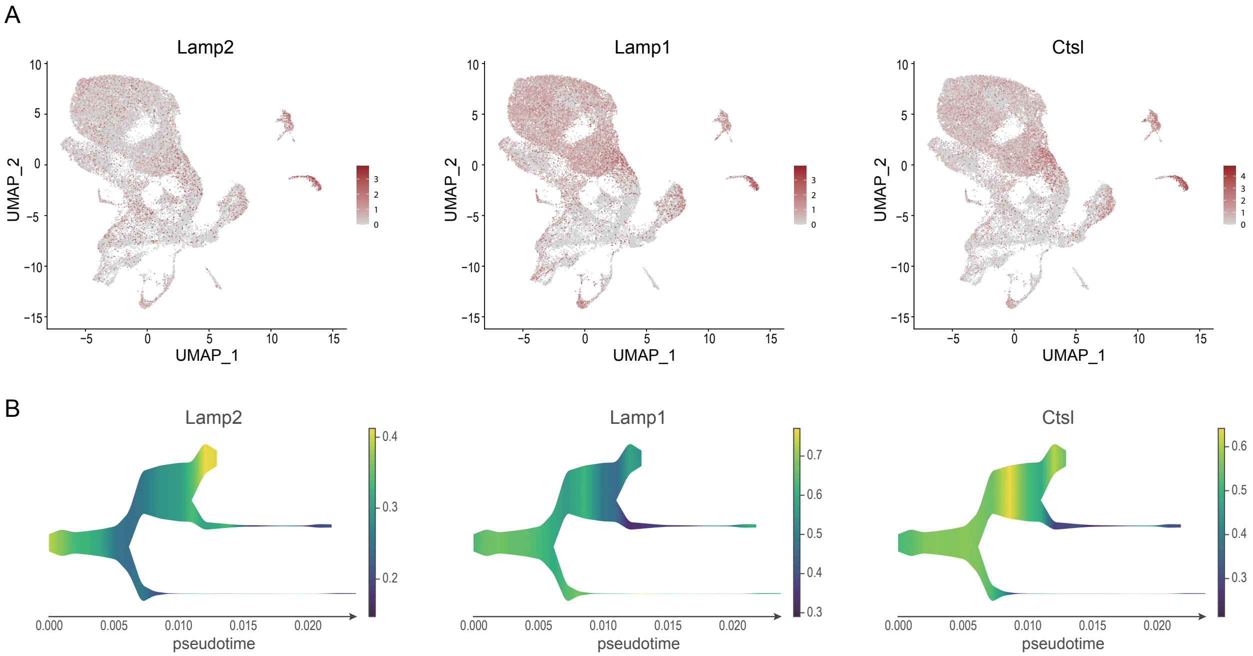
A
Lamp2
Lamp1
Ctsl
UMAP_1
UMAP_2
B
Lamp2
Lamp1
Ctsl
pseudotime
0.000
0.005
0.010
0.015
0.020
