# Supplementary material for: Integrative analysis of the embryonic origin of adult neural stem cells in forebrain subventricular zones
Source: Natl Sci Rev. 2026 Aug 29;13(18):nwag516. doi: 10.1093/nsr/nwag516 (PMC13629234; doi:10.1093/nsr/nwag516)
Supplement: Supplementary file 1 [file nwag516_supplemental_files.zip › supplementary files/figs2.170.wuhuan.0717.pdf]

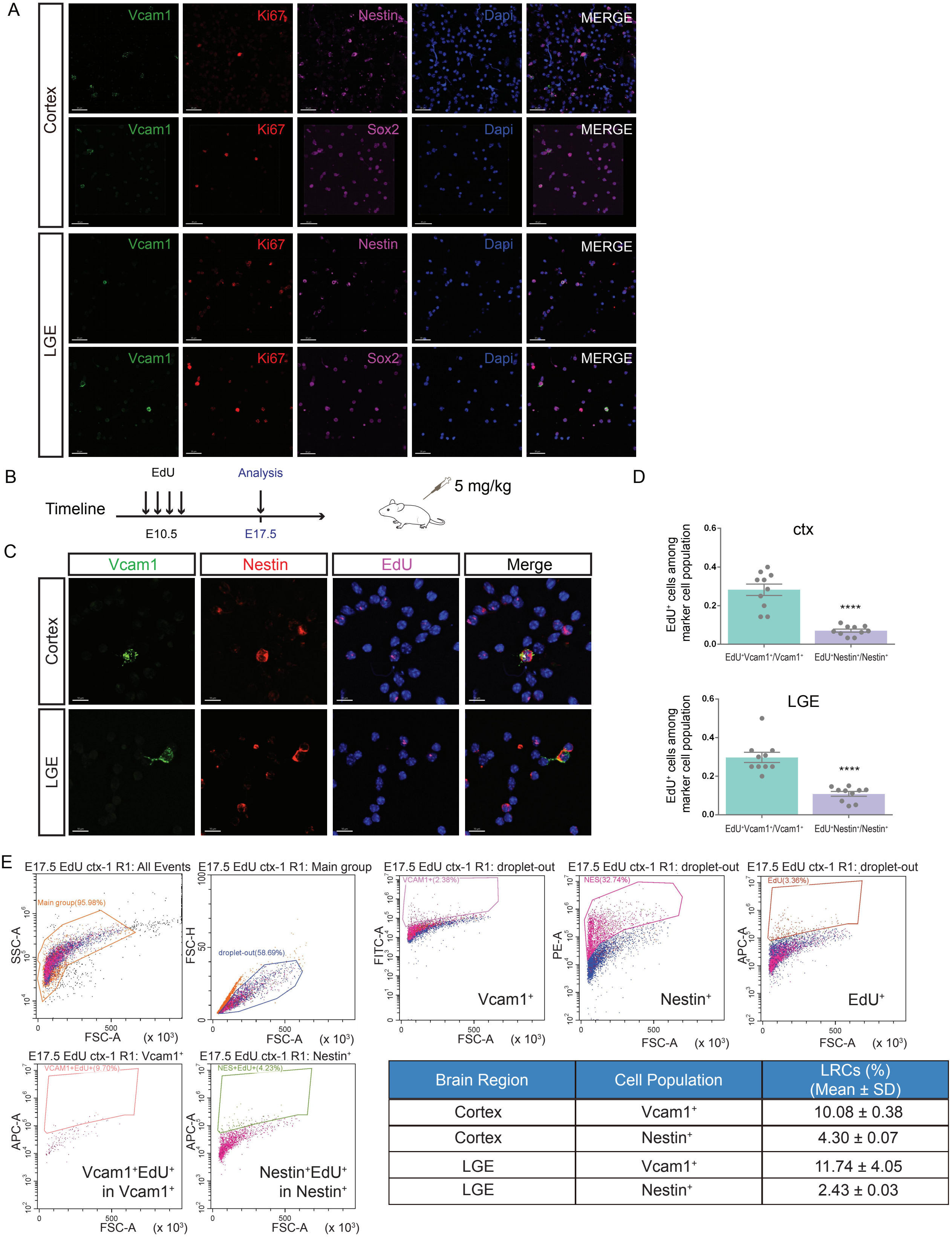

Brain Region	Cell Population	LRCs (%) (Mean ± SD)
Cortex	Vcam1$^+$	10.08 ± 0.38
Cortex	Nestin$^+$	4.30 ± 0.07
LGE	Vcam1$^+$	11.74 ± 4.05
LGE	Nestin$^+$	2.43 ± 0.03
