# Supplementary material for: Integrative analysis of the embryonic origin of adult neural stem cells in forebrain subventricular zones
Source: Natl Sci Rev. 2026 Aug 29;13(18):nwag516. doi: 10.1093/nsr/nwag516 (PMC13629234; doi:10.1093/nsr/nwag516)
Supplement: Supplementary file 1 [file nwag516_supplemental_files.zip › supplementary files/figs3.170.wuhuan.0717.pdf]

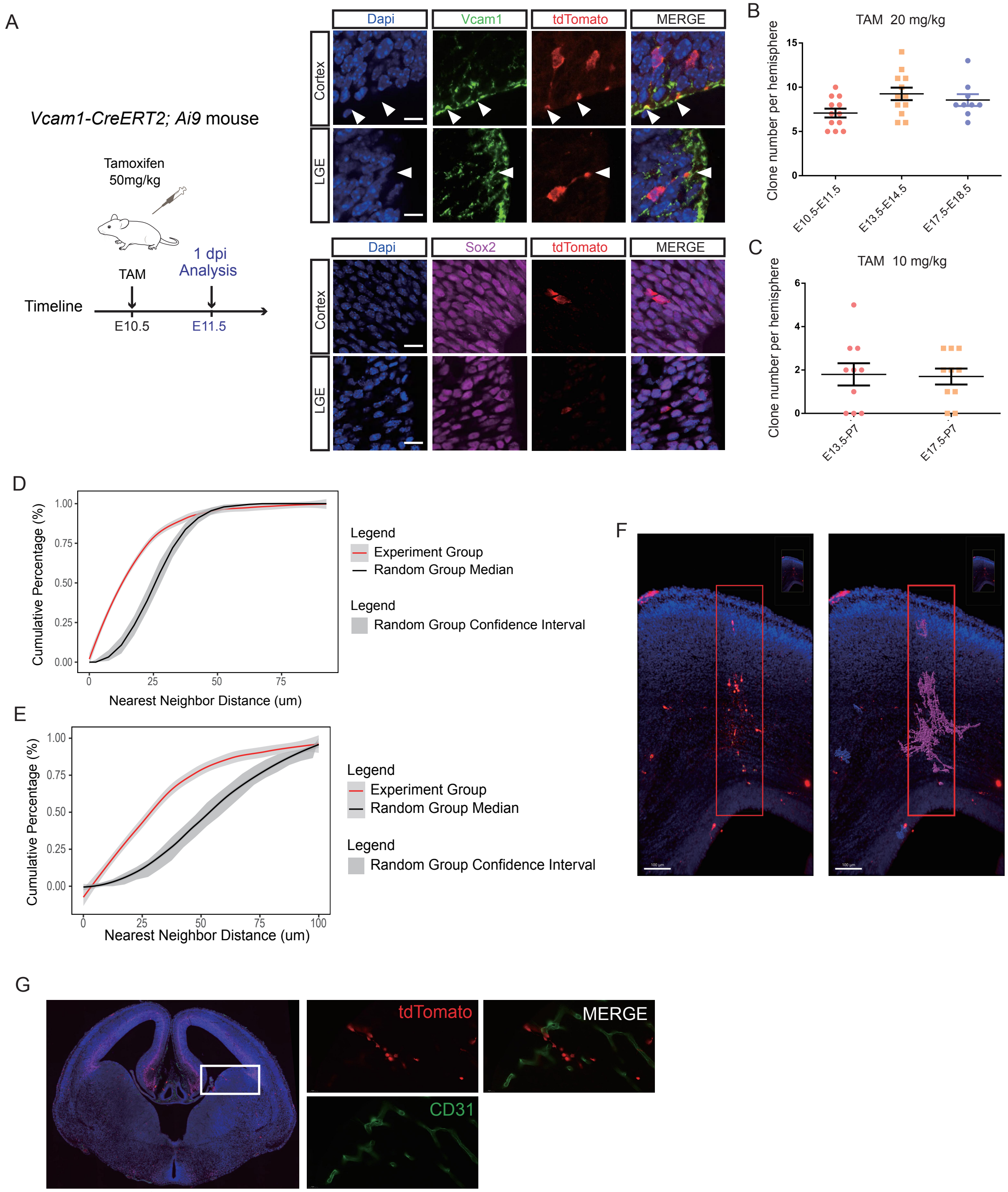
A
Vcam1-CreERT2; Ai9 mouse
Tamoxifen
50mg/kg
1 dpi
Analysis
TAM
Timeline
E10.5
E11.5
Dapi
Vcam1
tdTomato
MERGE
Cortex
LGE
Dapi
Sox2
tdTomato
MERGE
Cortex
LGE
B
TAM 20 mg/kg
Clone number per hemisphere
E10.5-E11.5
E13.5-E14.5
E17.5-E18.5
C
TAM 10 mg/kg
Clone number per hemisphere
E13.5-P7
E17.5-P7
D
Cumulative Percentage (%)
Nearest Neighbor Distance (um)
Legend
Experiment Group
Random Group Median
Legend
Random Group Confidence Interval
E
Cumulative Percentage (%)
Nearest Neighbor Distance (um)
Legend
Experiment Group
Random Group Median
Legend
Random Group Confidence Interval
F
G
tdTomato
MERGE
CD31
