# Supplementary material for: Integrative analysis of the embryonic origin of adult neural stem cells in forebrain subventricular zones
Source: Natl Sci Rev. 2026 Aug 29;13(18):nwag516. doi: 10.1093/nsr/nwag516 (PMC13629234; doi:10.1093/nsr/nwag516)
Supplement: Supplementary file 1 [file nwag516_supplemental_files.zip › supplementary files/figs4.170.wuhuan.0717.pdf]

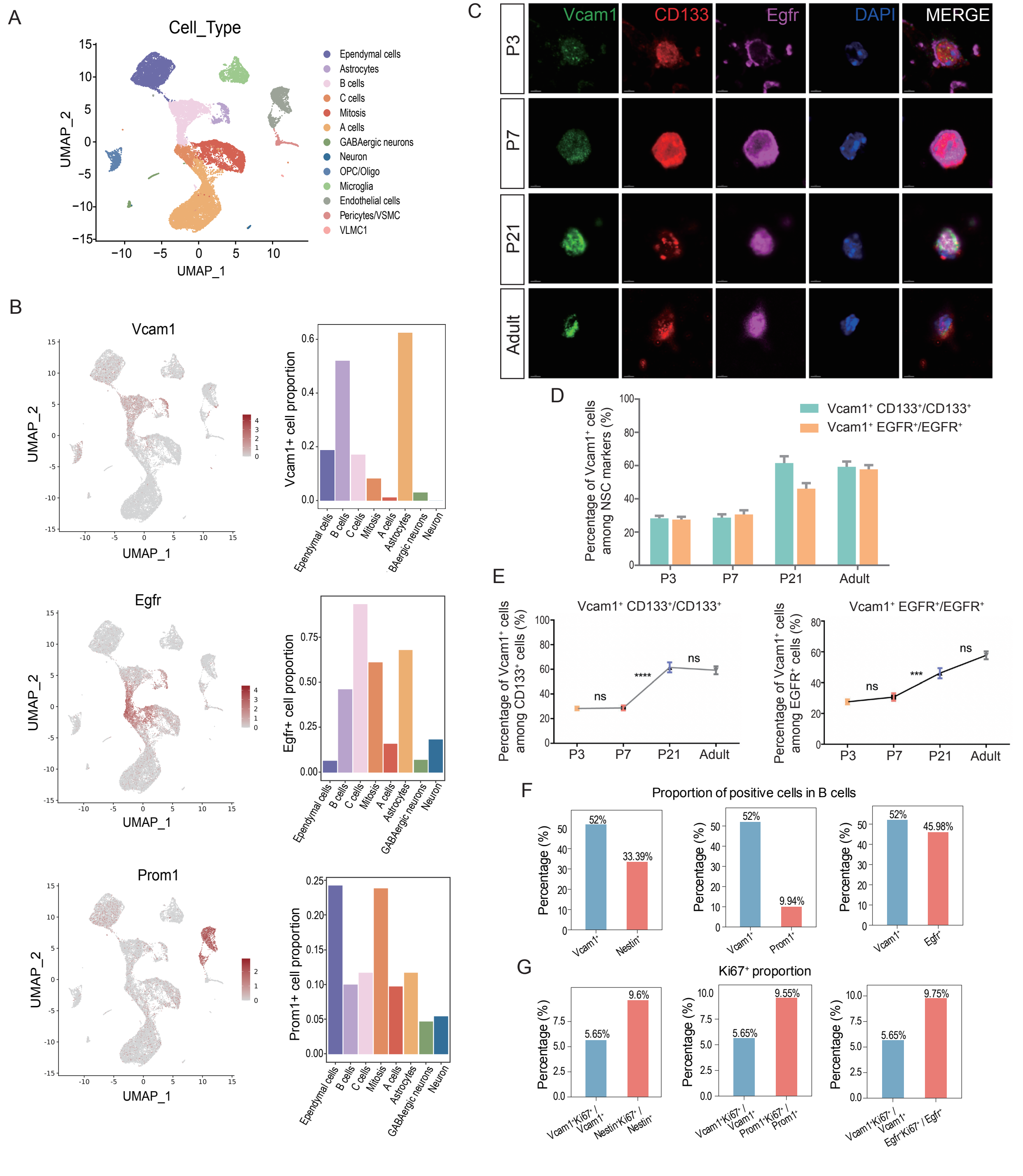

A
Cell_Type
Ependymal cells
Astrocytes
B cells
C cells
Mitosis
A cells
GABAergic neurons
Neuron
OPC/Oligo
Microglia
Endothelial cells
Pericytes/VSMC
VLMC1
UMAP_1
UMAP_2
B
Vcam1
Vcam1+ cell proportion
Egfr
Egfr+ cell proportion
Prom1
Prom1+ cell proportion
C
Vcam1
CD133
Egfr
DAPI
MERGE
P3
P7
P21
Adult
D
Vcam1+ CD133+/CD133+
Vcam1+ EGFR+/EGFR+
Percentage of Vcam1+ cells among NSC markers (%)
E
Vcam1+ CD133+/CD133+
Vcam1+ EGFR+/EGFR+
Percentage of Vcam1+ cells among CD133+ cells (%)
Percentage of Vcam1+ cells among EGFR+ cells (%)
ns
****
***
F
Proportion of positive cells in B cells
Percentage (%)
52%
33.39%
9.94%
45.98%
Vcam1+
Nestin+
Prom1+
Egfr+
G
Ki67+ proportion
5.65%
9.6%
9.55%
9.75%
Vcam1+Ki67+ / Vcam1+
Nestin+Ki67+ / Nestin+
Prom1+Ki67+ / Prom1+
Egfr+Ki67+ / Egfr+
