# Supplementary material for: Integrative analysis of the embryonic origin of adult neural stem cells in forebrain subventricular zones
Source: Natl Sci Rev. 2026 Aug 29;13(18):nwag516. doi: 10.1093/nsr/nwag516 (PMC13629234; doi:10.1093/nsr/nwag516)
Supplement: Supplementary file 1 [file nwag516_supplemental_files.zip › supplementary files/figs5.170.wuhuan.0717.pdf]

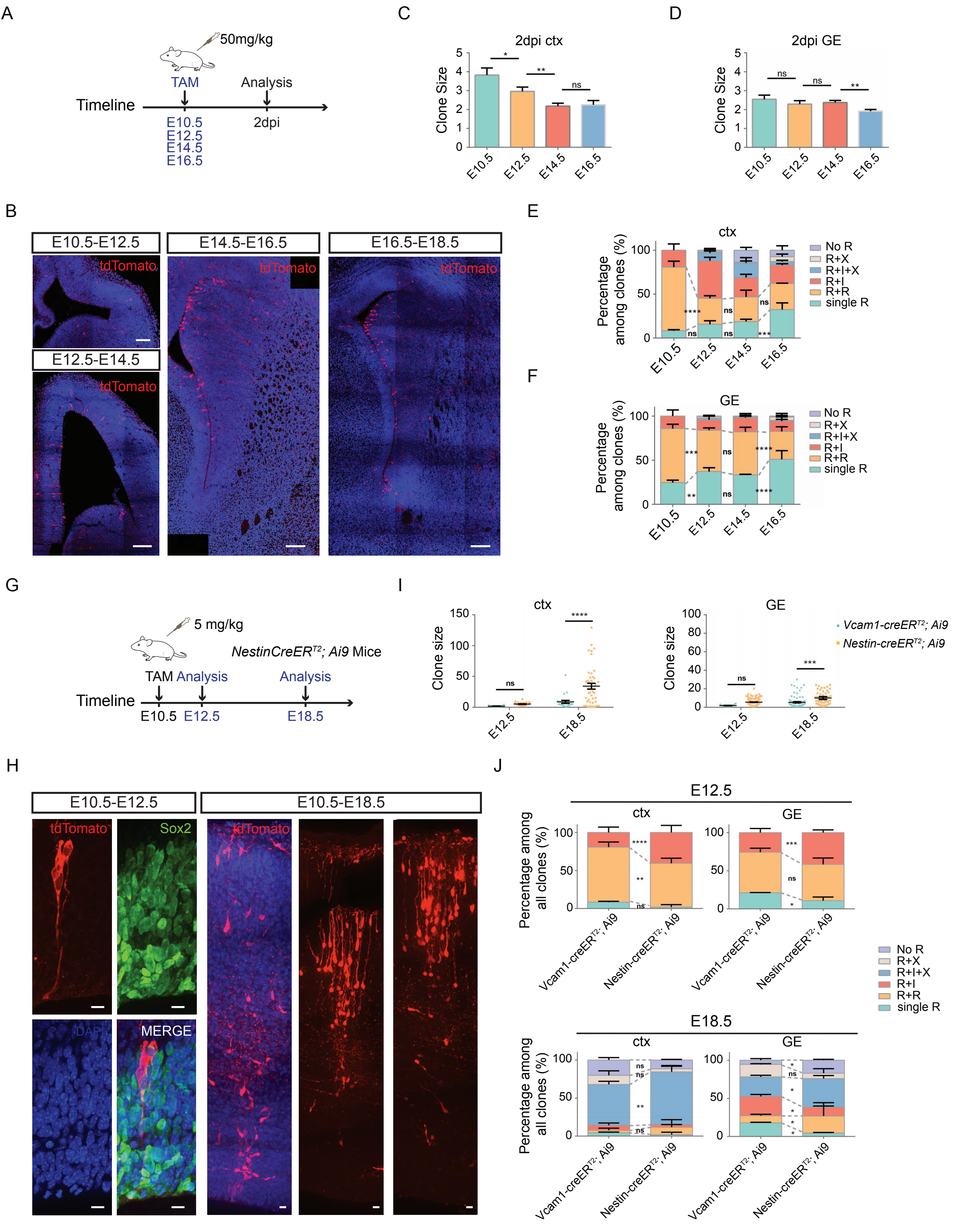

A
50mg/kg
TAM
Analysis
Timeline
E10.5
E12.5
E14.5
E16.5
2dpi
B
E10.5-E12.5
E12.5-E14.5
E14.5-E16.5
E16.5-E18.5
tdTomato
C
2dpi ctx
Clone Size
D
2dpi GE
E
ctx
Percentage among clones (%)
No R
R+X
R+I+X
R+I
R+R
single R
F
GE
G
5 mg/kg
NestinCreER$^{T2}$; Ai9 Mice
TAM Analysis
Analysis
Timeline
E10.5 E12.5
E18.5
I
ctx
GE
Clone size
Vcam1-creER$^{T2}$; Ai9
Nestin-creER$^{T2}$; Ai9
H
E10.5-E12.5
E10.5-E18.5
tdTomato
Sox2
DAPI
MERGE
J
E12.5
E18.5
ctx
GE
Percentage among all clones (%)
Vcam1-creER$^{T2}$; Ai9
Nestin-creER$^{T2}$; Ai9
