# Supplementary material for: Integrative analysis of the embryonic origin of adult neural stem cells in forebrain subventricular zones
Source: Natl Sci Rev. 2026 Aug 29;13(18):nwag516. doi: 10.1093/nsr/nwag516 (PMC13629234; doi:10.1093/nsr/nwag516)
Supplement: Supplementary file 1 [file nwag516_supplemental_files.zip › supplementary files/figs6.170.wuhuan.0717.pdf]

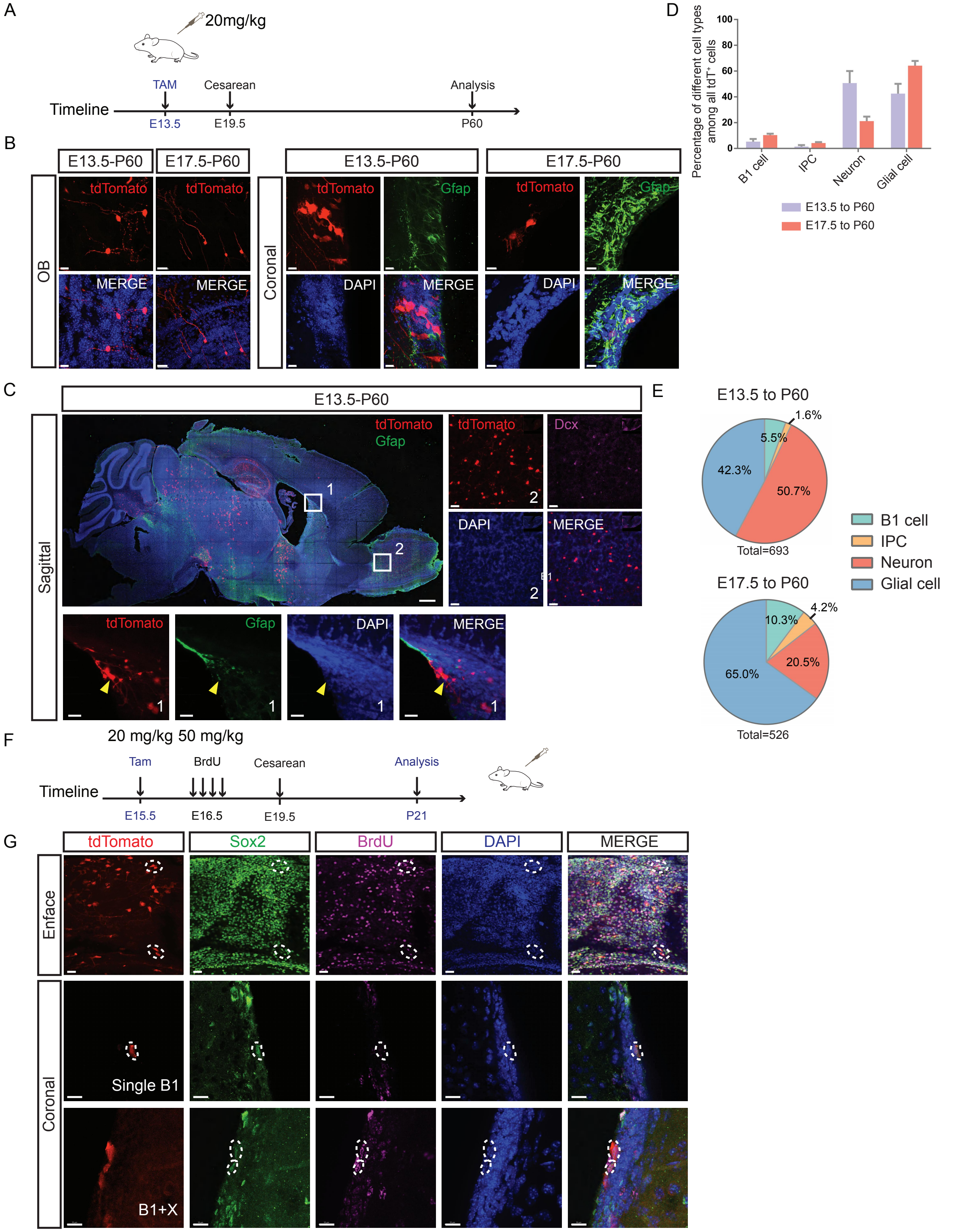

A
20mg/kg
Timeline
TAM
Cesarean
Analysis
E13.5
E19.5
P60
B
E13.5-P60
E17.5-P60
OB
tdTomato
MERGE
Coronal
Gfap
DAPI
C
Sagittal
Dcx
D
Percentage of different cell types among all tdT+ cells
100
80
60
40
20
0
B1 cell
IPC
Neuron
Glial cell
E13.5 to P60
E17.5 to P60
E
1.6%
5.5%
42.3%
50.7%
Total=693
4.2%
10.3%
65.0%
20.5%
Total=526
F
20 mg/kg 50 mg/kg
Tam
BrdU
E15.5
E16.5
P21
G
Sox2
BrdU
Enface
Single B1
B1+X
