# Supplementary material for: Integrative analysis of the embryonic origin of adult neural stem cells in forebrain subventricular zones
Source: Natl Sci Rev. 2026 Aug 29;13(18):nwag516. doi: 10.1093/nsr/nwag516 (PMC13629234; doi:10.1093/nsr/nwag516)
Supplement: Supplementary file 1 [file nwag516_supplemental_files.zip › supplementary files/figs7.170.wuhuan.0717.pdf]

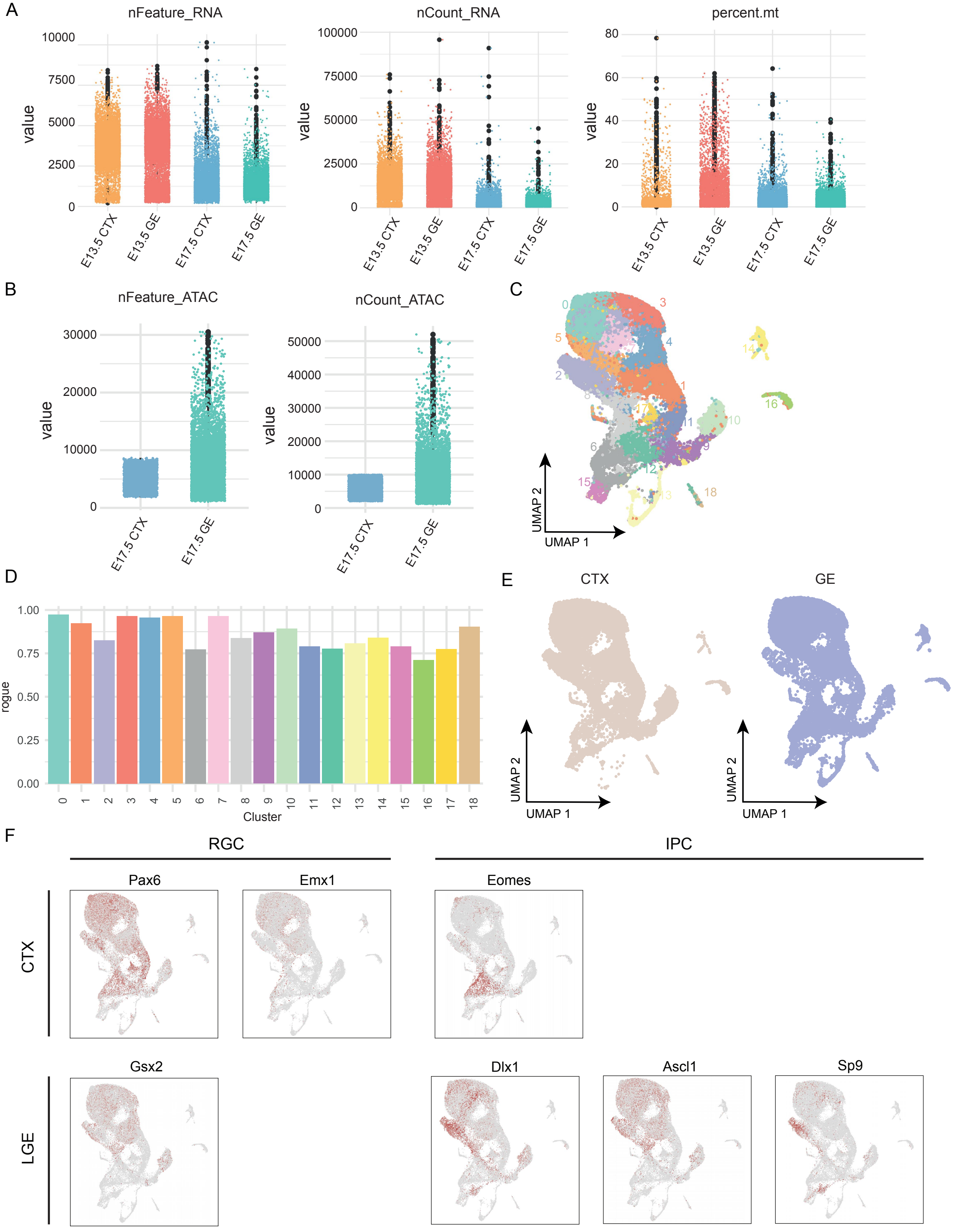

A
nFeature_RNA
nCount_RNA
percent.mt
value
E13.5 CTX
E13.5 GE
E17.5 CTX
E17.5 GE
B
nFeature_ATAC
nCount_ATAC
C
UMAP 1
UMAP 2
D
rogue
Cluster
E
CTX
GE
F
RGC
IPC
Pax6
Emx1
Eomes
Gsx2
Dlx1
Ascl1
Sp9
CTX
LGE
