# Supplementary material for: Integrative analysis of the embryonic origin of adult neural stem cells in forebrain subventricular zones
Source: Natl Sci Rev. 2026 Aug 29;13(18):nwag516. doi: 10.1093/nsr/nwag516 (PMC13629234; doi:10.1093/nsr/nwag516)
Supplement: Supplementary file 1 [file nwag516_supplemental_files.zip › supplementary files/figs8.170.wuhuan.0717.pdf]

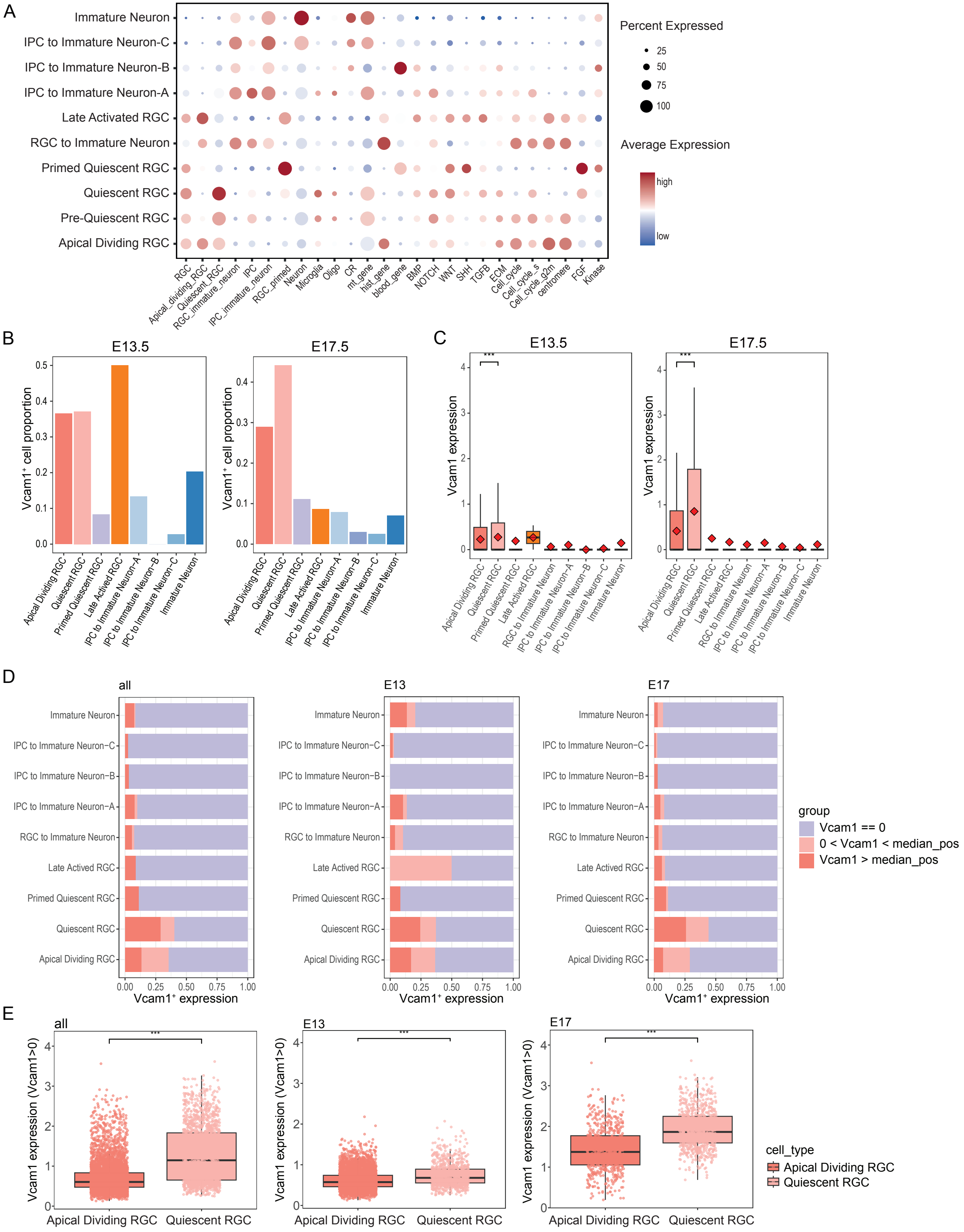

A
Immature Neuron
IPC to Immature Neuron-C
IPC to Immature Neuron-B
IPC to Immature Neuron-A
Late Activated RGC
RGC to Immature Neuron
Primed Quiescent RGC
Quiescent RGC
Pre-Quiescent RGC
Apical Dividing RGC
RGC
Apical_dividing_RGC
Quiescent_RGC
RGC_immature_neuron
IPC
IPC_immature_neuron
RGC_primed
Neuron
Microglia
Oligo
CR
mt_gene
hist_gene
blood_gene
BMP
NOTCH
WNT
SHH
TGFB
ECM
Cell_cycle
Cell_cycle_s
Cell_cycle_g2m
centromere
FGF
Kinase
Percent Expressed
25
50
75
100
Average Expression
high
low
B
E13.5
E17.5
Vcam1⁺ cell proportion
Apical Dividing RGC
Quiescent RGC
Primed Quiescent RGC
Late Actived RGC
IPC to Immature Neuron-A
IPC to Immature Neuron-B
IPC to Immature Neuron-C
Immature Neuron
C
E13.5
E17.5
Vcam1 expression
***
Apical Dividing RGC
Quiescent RGC
Primed Quiescent RGC
Late Actived RGC
RGC to Immature Neuron
IPC to Immature Neuron-A
IPC to Immature Neuron-B
IPC to Immature Neuron-C
Immature Neuron
D
all
E13
E17
Immature Neuron
IPC to Immature Neuron−C
IPC to Immature Neuron−B
IPC to Immature Neuron−A
RGC to Immature Neuron
Late Actived RGC
Primed Quiescent RGC
Quiescent RGC
Apical Dividing RGC
Vcam1⁺ expression
group
Vcam1 == 0
0 < Vcam1 < median_pos
Vcam1 > median_pos
E
all
E13
E17
Vcam1 expression (Vcam1>0)
***
Apical Dividing RGC
Quiescent RGC
cell_type
Apical Dividing RGC
Quiescent RGC
