# Supplementary material for: Integrative analysis of the embryonic origin of adult neural stem cells in forebrain subventricular zones
Source: Natl Sci Rev. 2026 Aug 29;13(18):nwag516. doi: 10.1093/nsr/nwag516 (PMC13629234; doi:10.1093/nsr/nwag516)
Supplement: Supplementary file 1 [file nwag516_supplemental_files.zip › supplementary files/figs9.170.wuhuan.0717.pdf]

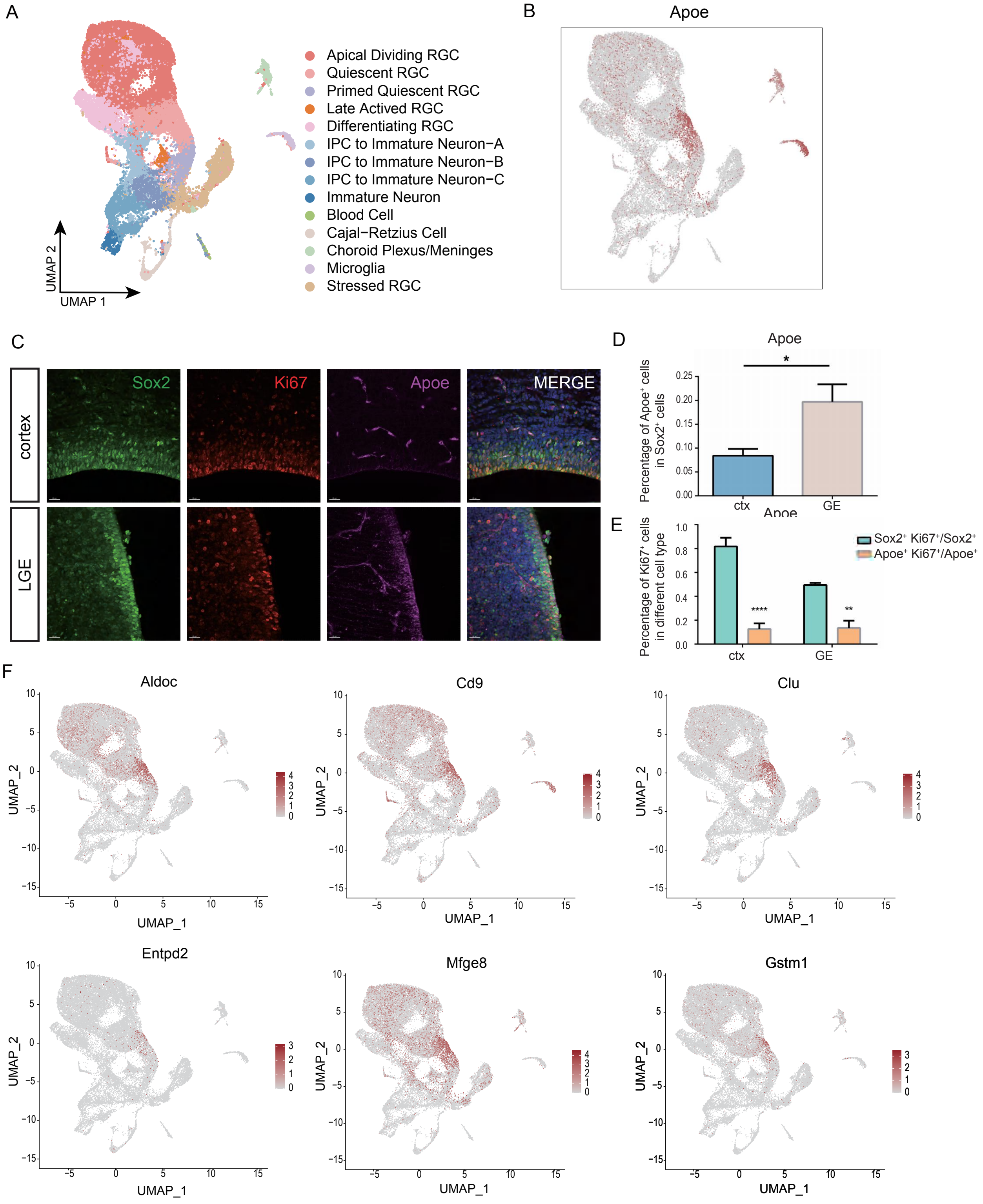

A
Apical Dividing RGC
Quiescent RGC
Primed Quiescent RGC
Late Actived RGC
Differentiating RGC
IPC to Immature Neuron−A
IPC to Immature Neuron−B
IPC to Immature Neuron−C
Immature Neuron
Blood Cell
Cajal−Retzius Cell
Choroid Plexus/Meninges
Microglia
Stressed RGC
UMAP 2
UMAP 1
B
Apoe
C
cortex
LGE
Sox2
Ki67
Apoe
MERGE
D
Apoe
Percentage of Apoe+ cells in Sox2+ cells
ctx
GE
E
Percentage of Ki67+ cells in different cell type
Sox2+ Ki67+/Sox2+
Apoe+ Ki67+/Apoe+
ctx
GE
F
Aldoc
Cd9
Clu
Entpd2
Mfge8
Gstm1
UMAP_1
UMAP_2
