# Supplementary material for: Integrative analysis of the embryonic origin of adult neural stem cells in forebrain subventricular zones
Source: Natl Sci Rev. 2026 Aug 29;13(18):nwag516. doi: 10.1093/nsr/nwag516 (PMC13629234; doi:10.1093/nsr/nwag516)
Supplement: Supplementary file 1 [file nwag516_supplemental_files.zip › supplementary files/Supplementary Figure Legends.docx]

### **Integrative Analysis of the Embryonic Origin of Adult Neural Stem Cells in Forebrain Subventricular Zones**

Huan Wu^1,†^, Ke Tang^2,†^, Ruimin Xu^1,†^, Tao Li^3^, Keke Liang^3^, Xuanxuan Wang^1^, Deyuan Huang^1^ , Yijia Yang^1^, Yanxuan Yin^1^, Jiayang Xie^1^，Chong Li^1^ , Xiaoyu Liu^1^, Qing-Ran Bai^2,*^, Chenfei Wang^2, *^, Qin Shen^2,4, ‡, *^ and Shaorong Gao^1, *^

1. *Shanghai Key Laboratory of Maternal and Fetal Medicine, Clinical and Translational Research Center of Shanghai First Maternity and Infant Hospital, School of Life Sciences and Technology, Tongji University, Shanghai 200092, China; Frontier Science Center for Stem Cell Research, Tongji University, Shanghai, 200092, China.*
2. *Key Laboratory of Spine and Spinal Cord Injury Repair and Regeneration of Ministry of Education, Orthopedic Department of Tongji Hospital, School of Life Science and Technology, Tongji University, Shanghai 200092, China; Frontier Science Center for Stem Cell Research, Tongji University, Shanghai, 200092, China.*
3. *Department of Neurology, Shanghai Tongji Hospital, School of Medicine, Tongji University, Shanghai 200092, China*
4. *Tongji University Brain and Spinal Cord Clinical Research Center, 200092, Shanghai, China*

^†^ Equally contributed to the work

^*^Corresponding authors. E-mails: baiqingran@tongji.edu.cn; 08chenfeiwang@tongji.edu.cn; shenqin@tongji.edu.cn; gaoshaorong@tongji.edu.cn

^‡^Deceased on May 31, 2021.

**Figure S1．Co-expression of Vcam1 Protein and EGFP in *Vcam1* Reporter Mice**

1. Representative images of EGFP (green) and Vcam1 (magenta) immunostaining at the indicated embryonic stages of the *Vcam1* Reporter mouse in the LGE. Scale bar, 20 μm. LGE: lateral ganglionic eminence.
2. Representative images of EGFP (green) and Vcam1 (magenta) immunostaining at the indicated embryonic stages of the *Vcam1* Reporter mouse in the cortex. Scale bar, 20 μm.
3. Schematic diagram of striatal wall of LV in sagittal and coronal view (top). En face view of the SVZ wholemount showing Vcam1 (red) on EGFP (green) cells in the center of pinwheel structures revealed by anti-Ctnnb (white) in *Vcam1* Reporter mouse at P4. Scale bar, 10 μm. OB, olfactory bulb; RMS, rostral migratory stream.
4. Representative image of EGFP^+^ and tdT^+^ cells in the brain of *Nestin-cre;Vcam1* Roporter mouse co-labeled with Vcam1 at E14.5. n = 4 brains. Scale bars, 200 μm, 10um.
5. Representative image of EGFP^+^ and tdT^+^ cells in the brain of *Emx1-cre;Vcam1* Roporter mouse co-labeled with Vcam1 at E14.5. n = 3 brains. Scale bars, 200 μm, 10um.

**Figure S2．Vcam1 exhibits higher specificity for labeling the quiescent NSC subpopulation during embryonic development**

1. Co-staining of Vcam1 with Nestin, Sox2, Ki67 in acutely isolated single cells from the cortex and LGE. Scale bar, 10um.
2. Schematic illustration of the EdU-label-retaining assay.
3. Co-staining of EdU(magenta) with Vcam1(green), Nestin(red) in acutely isolated single cells from the cortex and LGE. Owing to semiconservative DNA replication, the EdU label is halved with each cell cycle, resulting in a gradual transition from a pan‑nuclear to a punctate cytoplasmic pattern. Scale bar, 8um.
4. Statistic analysis of label-retaining EdU^+^ cells in Vcam1^+^ and other NSC population in the cortex and LGE at E17.5. Data were presented as mean ± SEM. Statistical significance was tested by two‐way ANOVA. ***P < 0.001. ****P < 0.0001.
5. Flow cytometry analysis of LRCs (EdU^+^) within Vcam1^+^ and Nestin^+^ populations in the cortex and LGE at E17.5. Gating strategies are shown, including main population, dropout, and marker-positive cell gates. For the cortex, the percentages of EdU^+^ LRCs among Vcam1^+^ cells were 9.7% and 10.45%, while those among Nestin^+^ cells were 4.23% and 4.37%. For the LGE, Vcam1^+^ cells contained 7.69% and 15.79% EdU^+^ LRCs, and Nestin^+^ cells contained 2.40% and 2.45% EdU^+^ LRCs.

**Figure S3．Verification of Cell Labeling Accuracy and Analysis of Cell Clones**

1. Schematic illustration of the tamoxifen induction assay (left). Representative images of tdTomato (red), Vcam1 (green) and Sox2 (magenta) immunostaining in the VZ region of tamoxifen induced brain (right). Arrowheads signifies the apical endfeet of tdT^+^ Vcam1^+^ cells. Scale bar, 10 μm. TAM, tamoxifen.
2. Cell numbers labeled by tamoxifen injection in *Vcam1-CreER^T2^;Ai9* mice. 20mg/kg Tamoxifen were used to induce embryonic clonal analysis. Each plot represents a hemisphere (E11.5, n = 12; E13.5 , n = 12; E17.5, n = 9).
3. Cell numbers labeled by tamoxufen injection in *Vcam1-CreER^T2^;Ai9* mice. 10mg/kg Tamoxifen were used to induce long-term lineage tracing from embryonic stage to postnatal. Each plot represents a hemisphere (E13.5 to P7, n = 10; E17.5 to P7, n = 10).
4. Clustering of laterally migrated cells. NND analysis of representative individual 3-dimensional reconstructed E18 brain slices with clonally labeled cells. Red lines represent experiment datasets and grey/black lines represent random simulations repeated 100 times.
5. Clustering of rostrally migrated cells. NND analysis of representative individual 3-dimensional reconstructed P7 brain slices with clonally labeled cells. Red lines represent experiment datasets and grey/black lines represent random simulations repeated 100 times.
6. Representative images of 3D surfaces for the cells constructed by Imaris (Scale bar: 100 μm).
7. Confocal images of CD31 (green) immunostaining at E14.5 (left). The boxed area is shown at a higher magnification (right). Box containing a clone in GE (bottom right). Scale bars, 200 μm (left) and 20 μm (right).

**Figure S4．Proportion of Vcam1^+^ cells in NSC Population from Birth to Adult**

1. UMAP of cells in adult V-SVZ colored by cluster from published single-cell RNA seq data [1].
2. The expression in UMAP and cell proportion of Vcam1^+^, Egfr^+^, CD133 (Prom1) ^+^ cells in each cluster.
3. Co-staining of Vcam1 with CD133 and Egfr in acutely isolated single cells at P3, P7, P21 and adult. Scale bars, 3 μm.

(D-E) [Quantitative analysis](https://www.sciencedirect.com/topics/biochemistry-genetics-and-molecular-biology/quantitative-technique) of the percentage of Vcam1-expressing cell in different cell types. Data represent mean ± SEM, (n = 3 repeats, at least 2,000 cells counted in each experiment, ∗∗∗p < 0.001, ∗∗∗∗p < 0.0001; One-way ANOVA with Tukey’s multiple comparision test).

1. Bar graphs showing the proportion of B cells positive for the indicated markers. For each pair, the percentage of Vcam1⁺ B cells (blue) is compared with the percentage of Nestin⁺, Prom1⁺, or Egfr⁺ B cells (red).
2. Bar graphs displaying the proportion of Ki67⁺ proliferating cells within the Vcam1⁺ population versus the Nestin⁺, Prom1⁺, or Egfr⁺ populations. All values are presented as percentages of the total B cell population, illustrating the relative proliferative activity of each marker-defined subset.

**Figure S5．Divergent Clonal Dynamics of Embryonic Vcam1^+^ Precursors Underlies Region-Specific Lineage Establishment**

1. Schematic illustration of the experimental paradigm used to label Vcam1^+^ RGCs in the embryonic. Timed pregnant *Vcam1-CreER^T2^;Ai9* mice were given a single injection of tamoxifen (TAM) to label embryos at E10.5, E12.5, E14.5 and E16.5, respectively. Brains were analyzed at 2 dpi of each group for clonal lineage-tracing (O)–(S).
2. Representative images of clones labeled at E10.5, E12.5, E14.5 and E16.5, respectively. Brain sections were stained with the antibody tdTomato(red) and with DAPI (blue). Scale bar, 300 μm.

(C-D) Quantifications of the clone size in the cortex (C) and GE (D) within two days. Values represent mean ± SEM (n = 3–6 brains; 60-100 total clone numbers, ∗p < 0.05, ∗∗p < 0.005; One-way ANOVA with Tukey’s multiple comparision test).

(E-F) Quantifications of the percentage of clone compositions in the cortex (E) and GE (F). Clones were classified into six categories, similar to Figure 3H-I. Values represent mean (n = 3–6 brains; 60-100 total clone numbers, ∗∗p < 0.005, ∗∗∗p < 0.001, ∗∗∗∗p < 0.0001; Two-way ANOVA with Tukey’s multiple comparision test).

(G) Experimental timeline for *Nestin-CreER^T2^;Ai9* lineage tracing. Tamoxifen (TAM, 5 mg/kg) was administered at E10.5, with analysis performed at E12.5 and E18.5.

(H) Representative immunofluorescence images of tdTomato-labeled clones in the developing cortex at E12.5 (left) and E18.5 (right). Scale bars, 5 um.

(I) Quantification of clone size in the cortex (ctx) and ganglionic eminence (GE) at E12.5 and E18.5. No significant difference in clone size was observed between *Vcam1-CreER^T2^;Ai9* and *Nestin-CreER^T2^;Ai9* lineages at E12.5. At E18.5, Nestin⁺ progenitors generated significantly larger clones than Vcam1⁺ cells in both regions, indicating greater long-term proliferative capacity.

(J) Classification of traced clones by cellular composition (single R, R+R, R+I, R+I+X, no R) in ctx and GE at E12.5 and E18.5. Data are presented as mean ± SEM. ns, not significant; *p < 0.05, **p < 0.01, ***p < 0.001, ****p < 0.0001.

**Figure S6．Long-Term Population Fate Mapping of Embryonic Vcam1^+^ Precursors to Postnatal and Adult NSCs in the V-SVZ**

1. Schematic illustration of the long-term fate mapping experimental paradigm. Timed pregnant *Vcam1-CreER^T2^;Ai9* mice were given a single injection of tamoxifen (TAM) to label embryos at E13.5 and E17.5, respectively. Live embryos were recovered by cesarean section at E19.5 and then brains were analyzed at P60 of each group for population fate mapping.
2. Confocal images of tdT^+^ interneurons in the OB (left) and tdT^+^ cells in V-SVZ (right) induced by TAM at E13.5 and E17.5, respectively. Brain sections were stained with the antibody tdTomato (red) Gfap (green) and with DAPI (blue). Scale bar, 10 μm.
3. Confocal images of tdT^+^ cells in the sagittal sections induced by TAM at E13.5 and stained with the antibodies tdTomato (red), Gfap (green) and DAPI (blue) at P60. The boxed areas are shown at a higher magnification of V-SVZ (Box1) and rostral migratory stream (RMS) towards OB (Box2). Scale bar, 200 μm, 30um.
4. The quantification of the tdT^+^ population of different cell types is shown. Values represent mean ± SEM (n=3 mouse brain in each experimental group, at least 9 sections are quantified in each brain, at least 30 z stacks in each section).
5. Quantitative comparison of the tdT^+^ population of different cell types between two experimental groups. Shown are pie charts of the percentage among all tdT^+^ cells among each cell type. Values represent mean (n=3 mouse brain in each experimental group, at least 9 sections are quantified in each brain, at least 30 z stacks in each section).
6. Experimental timeline. Tamoxifen (Tam, 20 mg/kg) was administered to *Vcam1-CreER^T2^;Ai9* embryos at E15.5 to induce tdTomato labeling in Vcam1⁺ cells. BrdU (50 mg/kg) was injected at E16.5 to label actively proliferating cells. Cesarean section was performed at E19.5, and brains were analyzed at postnatal day 21 (P21)
7. Representative immunofluorescence images of the lateral ventricle wall at P21. (Top) En face view of the ventricular wall, showing tdTomato (red, Vcam1 lineage), Sox2 (green, NSCs), BrdU (magenta, proliferative history), and DAPI (blue, nuclei). (Bottom) Coronal section views of the ventricular wall. The top outlined clone is a single tdTomato⁺Sox2⁺ B1 cell that is BrdU-negative, indicating continuous quiescence from E15.5 to P21. The bottom outlined clone contains BrdU-retaining tdTomato⁺Sox2⁺ B1 cells and their differentiated progeny, demonstrating transient dividing at E16.5 before entry into long-term quiescence. Scale bars, 10 μm.

**Figure S7. Heterogeneity of Vcam1^+^ NSCs revealed by scRNA-Seq**

1. Quality results of scRNA-Seq in different samples. The box plots show the number of reads, number of genes, and mitochondrial (mt) gene fraction per cell in each sample.
2. Quality results of scATAC-Seq in different samples. The box plots show the number of fragments and fraction of reads in peaks per cell in each sample.
3. UMAP of cells colored by unmerged cluster (C0-C18).
4. ROGUE values of unmerged 19 clusters, proving the quality of each putative cluster.
5. UMAP plots of cells from Cortex and LGE, respectively.
6. UMAP expression of specific regional and cell type markers.

**Figure S8. Integration of sub-clusters based on gene sets expression**

1. Gene set expression dot plot of the 10 filtered neurogenic cell clusters (Table S1). Dot size indicates the percentage of cells expressing each gene set, and dot color reflects average gene set expression (red, high; blue, low). Non-neurogenic cell populations and stressed mitochondrial signature-high RGCs were excluded from this analysis.
2. Bar plots showing the proportion of Vcam1^+^ cells within each cell subtype at E13.5 (left) and E17.5 (right). At both stages, Apical Dividing RGCs and Quiescent RGCs exhibit the highest fractions of Vcam1 expressing cells, with Quiescent RGCs showing the highest proportion at E17.
3. Box plots illustrating the Vcam1 expression levels across cell subtypes at E13.5 (left) and E17.5 (right). Red diamonds represent the mean expression value for each population, as the high frequency of Vcam1^-^ cells would obscure the trend in positive populations when using the median. At both stages, Quiescent RGCs display significantly higher mean Vcam1 expression compared to other subtypes, including Apical Dividing RGCs. Statistical significance was assessed using a two-sided Wilcoxon rank-sum test. p < 0.001
4. Bar plots showing the proportion of cells with different levels of Vcam1 expression across major cell subtypes. Cells were stratified into three groups: Vcam1 negative (Vcam1 == 0, blue), low positive (0 < Vcam1 < median_pos, light pink), and high positive (Vcam1 ≥ median_pos, red), where median_pos refers to the median expression of Vcam1 among all Vcam1^+^ cells. Data are presented for all stages combined (left), E13 (middle), and E17 (right).
5. Box plots comparing the expression level of Vcam1 (in Vcam1^+^ cells only) between Apical Dividing RGCs (AD RGCs) and Quiescent RGCs (Q RGCs). Q RGCs exhibit significantly higher Vcam1 expression than AD RGCs across all stages combined (left), E13 (middle), and E17 (right). Boxplots show the median (black line), interquartile range (box), and individual data points (dots). Statistical significance was assessed using a two-sided Wilcoxon rank-sum test. **p < 0.001.

**Figure S9. Validation of quiescent RGC markers**

1. UMAP of cells colored by cluster.
1. Expression patterns of Apoe in UMAP plot.
2. Immunofluorescent staining of Apoe at E17.5 in both the cortex and LGE. Sox2 and Ki67 were co‐stained. Scale bars: 20 μm.
3. Quantification of the percentage of Apoe^+^ cells ain Sox2^+^ cells, respectively. Values represent mean ± SEM (n = 3 brains in each group; ∗p < 0.05; unpaired t test).
4. Quantification of the percentage of Ki67^+^Apoe^+^ cells versus Ki67^+^Sox2^+^ cells, respectively. Values represent mean ± SEM (n = 3 brains in each group; ∗p < 0.05; unpaired t test).
5. Expression of quiescent NSC markers in the adult hippocampus [2] [3]

**Figure S10. Lineage specification of Vcam1^+^ NSCs along the pseudotime trajectory**

1. Developmental trajectories of cells in different time, region and samples.
2. Line charts showing transition proportion of cells from different time, region and samples along the pseudotime trajectory.
3. Pseudotemporal expression profiles of Vcam1, the proliferation marker Mki67 and canonical RGC markers (Sox2, Vim, Slc1a3/Glast). The quiescent RGC cluster at the trajectory endpoint is characterized by sustained expression of stem cell markers and downregulation of Mki67, confirming a quiescent state.
4. Pseudotemporal expression profiles of representative B1 cell markers (Zfp36, Maff, Anxa2) at E13.5 and E17.5 show significant enrichment along the S3–S4 trajectory, supporting that this branch corresponds to the developmental path leading to adult quiescent neural stem cells (B1 cells). These marker genes were derived from the set of genes enriched in adult V‑SVZ B1 cells versus parenchymal astrocytes, as reported by Cebrián‑Silla et al. [1].

**Figure S11. Potential fate determine molecules specific to branch S3-S4**

1. Heatmap of top 10 genes enriched in each branches.
2. Expression of predicted TFs along developmental pseudotime.
3. Expression of genes specific to branch S3-S4 along developmental pseudotime.
4. Expression of gene sets related to different pathways along developmental pseudotime.
5. Expression of Prdm16 along developmental pseudotime.

**Figure S12 Expression Pattern of Smad3**

1. Expression of TGF-β gene sets and Smad3 along developmental pseudotime.
2. Expression of Smad3 in the UMAP.
3. Representative images of Sox2 (red), Ki67 (green) and Smad3 (magenta) immunostaining in the cortex and LGE at E17.5. Scale bar, 15 μm.
4. In situ hybridization data of Smad3 in E11.5, E13.5, E15.5, E18.5. Source: Allen Institute for Brain Science, Allen brain atlas. [http://developingmouse.brain-map.org/gene/show/16897].

**Figure S13 Comparative analysis of Vcam1 and Nestin as NSC markers**

1. Expression of Nestin and Vcam1 on UMAP in embryonic single-cell RNA-seq data.
2. Statistic analysis of Ki67- cells in Vcam1+ and Nestin+ cell population (top row). And expression of Ki67 on UMAP(bottom row).
3. Expression of Nestin and Vcam1 on UMAP in published single-cell RNA-seq data from the adult V-SVZ region[1].

**Figure S14 Lysosomal-related genes showed relative enrichment in the quiescent cluster**

(A) Expression of lysosome-related genes on UMAP [4].

(B) Expression of lysosome-related genes along developmental pseudotime.

**Reference**

1.	Cebrian-Silla, A., et al., *Single-cell analysis of the ventricular-subventricular zone reveals signatures of dorsal and ventral adult neurogenesis.* Elife, 2021. **10**.

2.	Harris, L., et al., *Coordinated changes in cellular behavior ensure the lifelong maintenance of the hippocampal stem cell population.* Cell Stem Cell, 2021. **28**(5): p. 863-876.e6.

3.	Shin, J., et al., *Single-Cell RNA-Seq with Waterfall Reveals Molecular Cascades underlying Adult Neurogenesis.* Cell Stem Cell, 2015. **17**(3): p. 360-372.

4.	Berg, D.A., et al., *A Common Embryonic Origin of Stem Cells Drives Developmental and Adult Neurogenesis.* Cell, 2019. **177**(3): p. 654-668 e15.
