# Supplementary material for: Integrative analysis of the embryonic origin of adult neural stem cells in forebrain subventricular zones
Source: Natl Sci Rev. 2026 Aug 29;13(18):nwag516. doi: 10.1093/nsr/nwag516 (PMC13629234; doi:10.1093/nsr/nwag516)
Supplement: Supplementary file 1 [file nwag516_supplemental_files.zip › supplementary files/Supplementary Materials and Methods.docx]

**Integrative Analysis of the Embryonic Origin of Adult Neural Stem Cells in Forebrain Subventricular Zones**

Huan Wu^1,†^, Ke Tang^2,†^, Ruimin Xu^1,†^, Tao Li^3^, Keke Liang^3^, Xuanxuan Wang^1^, Deyuan Huang^1^ , Yijia Yang^1^, Yanxuan Yin^1^, Jiayang Xie^1^，Chong Li^1^ , Xiaoyu Liu^1^, Qing-Ran Bai^2,*^, Chenfei Wang^2, *^, Qin Shen^2,4, ‡, *^ and Shaorong Gao^1, *^

1. *Shanghai Key Laboratory of Maternal and Fetal Medicine, Clinical and Translational Research Center of Shanghai First Maternity and Infant Hospital, School of Life Sciences and Technology, Tongji University, Shanghai 200092, China; Frontier Science Center for Stem Cell Research, Tongji University, Shanghai, 200092, China.*
2. *Key Laboratory of Spine and Spinal Cord Injury Repair and Regeneration of Ministry of Education, Orthopedic Department of Tongji Hospital, School of Life Science and Technology, Tongji University, Shanghai 200092, China; Frontier Science Center for Stem Cell Research, Tongji University, Shanghai, 200092, China.*
3. *Department of Neurology, Shanghai Tongji Hospital, School of Medicine, Tongji University, Shanghai 200092, China*
4. *Tongji University Brain and Spinal Cord Clinical Research Center, 200092, Shanghai, China*

^†^ Equally contributed to the work

^*^Corresponding authors. E-mails: [baiqingran@tongji.edu.cn](mailto:baiqingran@tongji.edu.cn); 08chenfeiwang@tongji.edu.cn; [shenqin@tongji.edu.cn](mailto:shenqin@tongji.edu.cn); [gaoshaorong@tongji.edu.cn](mailto:gaoshaorong@tongji.edu.cn)

^‡^ Deceased on May 31, 2021.

**Supplementary Materials**

**Antibodies**

| **REAGENT or RESOURCE** | **SOURCE** | **IDENTIFIER** |
| --- | --- | --- |
| Rat monoclonal anti-VCAM1 | BD Bioscience | Cat# 550547; RRID: AB_393741 |
| Mouse monoclonal anti-Pax6 | Santa Cruz | Cat# sc-81649;RRID:AB_1127044 |
| Goat polyclonal anti-Doublecortin (C-18) | Santa Cruz | Cat# sc-8066; RRID: AB_2088494 |
| Mouse monoclonal anti-SOX2 | Thermo Fisher Scientific | Cat# MA1014;RRID:AB_2536667 |
| Rabbit monoclonal anti-Ki67 | Thermo Fisher Scientific | Cat# RM-9106-s1;RRID:AB_149792 |
| Rabbit polyclonal anti-Tbr2/Eomes | Abcam | Cat# ab23345;RRID:AB_778267 |
| Mouse monoclonal anti-[Mash1](https://scicrunch.org/resources/data/record/nif-0000-07730-1/RRID:AB_2572887/resolver?q=Mash1*&i=rrid:ab_2572887-2508343)/Ascl1 | Thermo Fisher Scientific | Cat# 14-5794-82 (also 14-5794), RRID:AB_2572887 |
| Rat monoclonal anti-BrdU | Abcam | Cat# ab6326;RRID:AB_305426 |
| Chicken polyclonal anti-GFP | Abcam | Cat# ab13970;RRID:AB_300798 |
| Goat polyclonal anti-tdTomato | SICGEN | Cat# AB8181-200; RRID:AB_2722750 |
| Mouse monoclonal anti-Nestin | Developmental study Hybridoma bank | Cat# rat-401;RRID:AB_2235915 |
| Rabbit polyclonal anti-GFAP | Dakotomation | Cat# Z0334;RRID:AB_10013382 |
| Rabbit polyclonal anti-Olig2 | Millipore | Cat# AB15328, RRID:AB_2299035 |
| [Mouse monoclonal anti-S-100 beta](https://scicrunch.org/resources/data/record/nif-0000-07730-1/AB_632378/resolver?q=S100B&l=S100B&facet%5b%5d=Host%20Organism:mouse&facet%5b%5d=Vendor:santa%20cruz%20biotechnology&i=rrid:ab_632378-2097389) | Santa Cruz | Cat# sc-58841, RRID:AB_632378 |
| [Mouse monoclonal anti-C](https://scicrunch.org/resources/data/record/nif-0000-07730-1/AB_632378/resolver?q=S100B&l=S100B&facet%5b%5d=Host%20Organism:mouse&facet%5b%5d=Vendor:santa%20cruz%20biotechnology&i=rrid:ab_632378-2097389)D31/PECAM-1 | BD Biosciences | Cat# 550300, RRID:AB_393595 |
| [Mouse monoclonal anti-Catenin, beta](https://scicrunch.org/resources/data/record/nif-0000-07730-1/RRID:AB_397560/resolver?q=catenin&i=rrid:ab_397560-2022253) | BD Biosciences | Cat# 610159, RRID:AB_397560 |
| Goat anti-Mouse IgG, Alexa Fluor 488 | Invitrogen | Cat# A-11029;RRID:AB_2534088 |
| Goat anti-Mouse IgG, Alexa Fluor 546 | Invitrogen | Cat# A-11030;RRID:AB_2534089 |
| Goat anti-Mouse IgG, Alexa Fluor 647 | Invitrogen | Cat# A-21236;RRID:AB_2535805 |
| Donkey anti-Rabbit IgG, Alexa Fluor 488 | Invitrogen | Cat# A-21206;RRID:AB_2535792 |
| Donkey anti-Rabbit IgG, Alexa Fluor 546 | Invitrogen | Cat# A-10040;RRID:AB_2534016 |
| Donkey anti-Rabbit IgG, Alexa Fluor 647 | Invitrogen | Cat# A-31573;RRID:AB_2536183 |
| Goat anti-Rat IgG, Alexa Fluor 555 | Invitrogen | Cat# A-21434;RRID:AB_2535855 |
| Donkey anti-Goat IgG, Alexa Fluor 546 | Invitrogen | Cat# A-11056;RRID:AB_2534103 |

**Chemicals, peptides, and recombinant proteins**

| **REAGENT or RESOURCE** | **SOURCE** | **IDENTIFIER** |
| --- | --- | --- |
| Tamoxifen | Sigma | Cat#T5648 |
| Sesame oil | Sigma | Cat#S3547 |
| Paraformaldehyde (PFA) | Sigma | Cat#158127 |
| DAPI | Invitrogen | Cat#1612980 |
| N2 | Invitrogen | Cat#17502048 |
| B27 | Invitrogen | Cat#17504044 |
| Basic Fibroblast Growth Factor (bFGF) | Invitrogen | Cat#PHG0261 |
| N-acetyl-L-cysteine (NAC) | Amresco | Cat#0108-25G |
| Deoxyribonuclease (Dnase I) | Takara | Cat#2212-1000U |
| Poly-L-Lysine (PLL) | Sigma | Cat#P4707-50ml |
| Triton X-100 | Sigma | Cat#9002-93-1 |
| Papain | Worthington | Cat#LS003126-100g |
| Fluoromount-G | Thermo Fisher Scientific | Cat#0100-01 |
| Superfect | QIAGEN | Cat#301307 |
| Fastgreen | Sigma | Cat#F7258-25g |
| TRIzol | GIBCO | Cat#15596018 |
| Sorbitol | Sigma | Cat#S8143-1kg |
| Na-pyruvate | Sigma | Cat#P-5280 |
| O.C.T. compound | Sakura | Cat#4583 |
| Proteinase K | Thermo Fisher Scientific | Cat#25530049 |

**Experimental models**

| **REAGENT or RESOURCE** | **SOURCE** | **IDENTIFIER** |
| --- | --- | --- |
| Vcam1-Reporter^T2A-loxP-EGFP-WPRE-polyA-loxP-tdTomato-WPRE^ | This paper | N/A |
| Vcam1-CreERT2^T2A-CreERT2^ | This paper | N/A |
| Ai9 | Jackson Laboratory  [1] | B6.Cg-Gt(ROSA)26Sortm9(CAG-tdTomato)Hze/J; RRID: IMSR_JAX:007909 |
| Nestin-Cre | Jackson Laboratory [2] | B6.Cg-Tg(Nes-cre)1Kln)/J; RRID:IMSR_JAX:003771 |
| Emx1-IRES-Cre | Jackson Laboratory  [3] | B6.129S2-Emx1tm1(cre)Krj/J; RRID: IMSR_JAX:005628 |
| Nestin-CreERT2 | Cyagen | B6.JGpt-Tg(Nes-CreERT2)/J; Stock No:C001422 |
| CAG-Cre | GemPharmatech | B6.JGpt-Tg(CAG-Cre)/Gpt; Stock No:T004055 |
| ICR | Vital River | CD-1(ICR) IGS |

**Oligonucleotides**

| **REAGENT or RESOURCE** | **SOURCE** | **IDENTIFIER** |
| --- | --- | --- |
| VCAM1-F: AGTTGGGGATTCGGTTGTTCT | This paper | N/A |
| VCAM1-R: CCCCTCATTCCTTACCACCC | This paper | N/A |
| VReporter-F5: GAGTCTCGATGGTCCTCAGTGC | This paper | N/A |
| VReporter-R5: GGAAGTCTGAATGCATGGCTTG | This paper | N/A |
| VReporter-R6: CAGATGAACTTCAGGGTCAGCTT | This paper | N/A |
| VReporter-R7: TCTTTGATGACCTCCTCGCCCTT | This paper | N/A |
| VCreER-F: AGTGAAATCTCCAAAGACCATCAGT | This paper | N/A |
| VCreER-R: TAGCCATGAACAGAAATGCCTAAC | This paper | N/A |
| Ai9-F: CTGTTCCTGTACGGCATGG | This paper | N/A |
| Ai9-R: GGCATTAAAGCAGCGTATCC | This paper | N/A |
| GFAP-F: CAGGCTGATGTGTGGGAACT | This paper | N/A |
| GFAP-R: GCAAGGTGCTGGGAATTTGG | This paper | N/A |
| Nestin-WT-F: TTGCTAAAGCGCTACATAGGA | This paper | N/A |
| Nestin-WT-R: GCCTTATTGTGGAAGGACTG | This paper | N/A |
| Emx1-Cre-F: GTGATGCTCTCTTCTGCCTGG | This paper | N/A |
| Emx1-Cre-R: CAGCCAGCCCATTCTCTTGTC | This paper | N/A |
| cre-F: TTCCTCGAGGCCACCATGGCCAATTTACTGACCG | This paper | N/A |
| cre-R: TCCGCGGCCGCCAGATCTTCTTCAGAAATAAG | This paper | N/A |
| NestinCreER-F: CACTTTGGTTCTTCTCTTCTGCAC | This paper | N/A |
| NestinCreER-R: GAAGCATTTTCCAGGTATGCTCAGAA | This paper | N/A |

**Software and algorithms**

| **REAGENT or RESOURCE** | **SOURCE** | **IDENTIFIER** |
| --- | --- | --- |
| GraphPad Prism | GraphPad Software | N/A |
| ZEN | Zeiss | N/A |
| ImageJ | NIH | N/A |
| Imaris | Bitplane | N/A |
| Seurat V4.3.0 | [4] | https://satijalab.org/seurat/ |
| MAESTRO V1.5.1 | [5] | https://liulab-dfci.github.io/MAESTRO/ |
| clusterProfiler V4.2.2 | [6] | https://bioconductor.org/packages/release/bioc/html/clusterProfiler.html |
| Signac V1.7.0 | [7] | https://stuartlab.org/signac/ |
| Rsamtools V2.10.0 | [8] | https://bioconductor.org/packages/release/bioc/html/Rsamtools.html |
| STREAM V1.1.0 | [9] | https://github.com/pinellolab/STREAM |
| EnsDb.Mmusculus.v79 V2.99.0 | [10] | https://bioconductor.org/packages/release/data/annotation/html/EnsDb.Mmusculus.v79.html |
| LISA V2.3.0 | [11] | https://github.com/liulab-dfci/lisa2 |
| Cistrome Data Browser | [12] | http://cistrome.org/db/ |

**Supplementary Methods**

**Generation of Vcam1-reporter and Vcam1-CreERT2 alleles**

For the *Vcam1* reporter allele, a *T2A-loxP-EGFP-WPRE-polyA-loxP-tdTomato-WPRE* cassette was inserted between the coding sequences of exon 9 and the 3’UTR of the Vcam1 gene using a CRISPR/Cas9 strategy. The sgRNA, targeting vector, and Cas9 mRNA were injected into C57BL/6 zygotes. Founder screening was performed by PCR to detect integration, and positive junctions were confirmed by Sanger sequencing. Correct targeting was further validated by Southern hybridization.

For the *Vcam1-CreER^T2^* allele, a *T2A-CreERT2* cassette was inserted into the same genomic locus via homologous recombination in embryonic stem (ES) cells. Correctly targeted ES clones were identified by PCR and Southern blot, and subsequently injected into blastocysts to generate chimeric mice.

**Detailed tamoxifen, BrdU and EdU protocols**

Tamoxifen (Sigma) was dissolved in corn oil with 10% ethanol at 37 °C with occasional vortexing to prepare a 20 mg/mL stock. For timed pregnancies, E0.5 was determined by vaginal plug detection. For postnatal fostering of embryonically injected mice, live embryos were recovered by cesarean section at E19.5 and raised by ICR foster mothers. For BrdU (Sigma) detection, frozen sections were pretreated with 2N HCl for 30 min at 37 °C prior to immunofluorescence staining for BrdU. To label slowly dividing cells, EdU was injected into pregnant mice 4 times at 2hr interval at E10.5. Embryos were removed and fixed at E17.5 for EdU [staining](https://www.sciencedirect.com/topics/neuroscience/staining-technique) (Thermo, C10634).

**Wholemount staining details**

SVZ wholemounts were dissected from postnatal brains following established protocols. [13, 14]. After overnight fixation in 4% PFA at 4 °C, tissues were blocked in PBS containing 5% BSA and 0.3% Triton X-100 for 1 h at RT. Primary antibodies were incubated for 48 h at 4 °C, followed by three 10-min washes in PBS/0.3% Triton. Secondary antibody incubation lasted 24 h at RT. Nuclei were counterstained with DAPI, and wholemounts were mounted with Prolong Gold antifade (Life Tech) using 100 μm adhesive spacers.

**Acute cell staining and dissociation**

Embryonic cortex or LGE were dissected and dissociated into single cells. Cells were seeded onto poly-L-lysine (PLL)-precoated coverslips. To minimize in vitro differentiation, cells were fixed with 4% PFA for 15 min immediately after adherence (3 h post-plating), then stained and reversely mounted onto slides.

**Detailed flow cytometry gating strategy**

Sequential gating was applied: events were first separated into main cell population and debris based on FSC-A/SSC-A plots. The gated population was further analyzed for VCAM1⁺, Nestin⁺, and EdU⁺ subsets. For each channel, the positive threshold was set where the test sample signal exceeded the vast majority of corresponding isotype-control events. The same hierarchy and thresholds were applied consistently across all samples.

**Clonal analysis criteria and NND parameters**

To ensure sparse labeling, we adjusted the dose of tamoxifen to label fewer than 10 clones per hemisphere. Serial coronal brain sections through the entire forebrain were cut using a cryostat. Every section was kept in order. All sections were stained with tdTomato antibody to locate cells within clones. Each clone was imaged in its entirety, and cells within the clone were morphologically assessed. Individual sections were then re-stained with specific marker antibodies to confirm cell type, followed by a second round of imaging..

Under sparse labeling conditions, the physical distance between independent clones is large, and incidental contact of processes from different clones is extremely unlikely. Therefore, when we observed direct process connections among labeled cells within a spatially restricted cluster, we interpreted this as evidence that these cells belong to the same clonal unit. This approach, combined with the low clonal density, served as a reliable indicator of lineage relationship.

We did not assume that cortical progenitors give rise only to cortical cells or that LGE cells only populate the striatum/cortex. Instead, clone origin (cortex vs. LGE) was determined primarily by migration path, not by terminal location:

· Radial migration (cells migrating perpendicular to the ventricular/pial surface) → cortical origin.

· Tangential migration (cells migrating parallel to the ventricular/pial surface) → LGE origin.

The nearest neighbor distance (NND) analysis were performed to the three-dimensional reconstruction data sets to quantitatively analyze the distribution of clonally labeled progenies. We used Imaris for automatic cell recognition and nearest neighbor distance analysis in three-dimensional images of each hemisphere. The laterally migrated cluster was defined as the columnar/radial assembly of neurons perpendicular to the neocortical pial surface. And the rostrally migrated cluster was defined as the lateral assembly of neurons parallel to the neocortical pial surface.

For detecting laterally migrated (radial) clusters, a rectangular cuboid with a lateral width of 200 μm (X-axis) and a vertical length corresponding to the radial length of the cortex between the pial surface and the ventricular zone (600-800 μm at different embryonic stages, 1,000 μm at P7 and 1,200 μm at P21-30; Y-axis) was used to scan through every three consecutive sections (45 μm each; Z-axis) along the pial surface. The 200 µm lateral width was determined based on the observed maximum distribution width of individual clones in the tangential plane[15]. This threshold ensures that all cells within a laterally dispersed clone are encompassed within the analysis. The 600–1200 µm vertical length corresponds to the average maximum distance from the ventricular zone to the pial surface across embryonic to postnatal developmental stages. By defining a rectangular cuboid with these dimensions, we aimed to capture the full spatial extent of clonally related cells migrating radially from the ventricular zone to the cortical plate.

For rostrally migrated(tangential) cells, a similar process was used involving cylinders with a cylindrical length of 1000 μm and a diameter of 200 μm. The 200 µm diameter and 800 µm cylindrical length were derived from empirical measurements of RMS geometry and migratory dispersion (Allen Brain Atlas). These parameters define 3-5 sequential cylindrical segments along the RMS axis, ensuring that cells within each segment are positioned along the migratory pathway while accounting for natural path curvature and dispersion. We compared the data sets of clonally labeled progenies with spatially random simulated data sets containing the same number of data points within the same volume repeated 100 times.

Cell types within clones were assessed by a combination of marker expression and morphology. In the developing brain, neural progenitors were defined by their expression of Nestin and Sox2 and/or by the presence of a process anchored to the ventricle. IPCs were defined by their expression of Tbr2 in cortex and Ascl1 in LGE. In postnatal and adult, B1 cells were defined by their expression of GFAP with apical process anchored to the ventricle. IPCs were defined by their expression of Tbr2 or Ascl1 and their horizontal morphology with few short processes. Glial cells were defined by their morphology.

**In utero electroporation (IUE) and rescue experiments**

Timed-pregnant mice were anesthetized by [isoflurane](https://www.sciencedirect.com/topics/biochemistry-genetics-and-molecular-biology/isoflurane) and put on a heated pad. The uterine [horn](https://www.sciencedirect.com/topics/agricultural-and-biological-sciences/horn) was exposed and 0.5-1 μL of plasmid solution (2-3 μg/μl) mixed with Fast Green dye (Sigma) was injected into the [lateral ventricle](https://www.sciencedirect.com/topics/neuroscience/lateral-ventricle) of the embryonic brain with a fine glass micropipette. Embryos were then electroporated by using ECM830 Electroporation system (BTX). In rescue experiments of Smad3, a Smad3-WT plasmid and a Smad3-Mut plasmid were mixed at 1:1 ratio and injected into the lateral ventricle.

**scRNA-Seq and scATAC-Seq library preparation**

Brains were rapidly dissected in ice-cold HIB solution (30 mM KCl, 5 mM NaOH, 5 mM NaH₂PO₄, 0.5 mM MgCl₂, 20 mM Na Pyruvate, 5.5 mM Glucose, 200 mM Sorbitol). Tissues were digested with papain (Worthington) in DMEM containing NAC (Amresco) for 20 min at 37 °C, with gentle trituration (5-10 times) using a 1-mL pipette. After digestion, cells were washed three times with DMEM and centrifuged at 400g for 10 min. TdT⁺ cells were sorted on a BD FACS Aria III with a 100-µm nozzle. E13.5 samples pooled 22 pups; E17.5 pooled 18 pups.

**Procession of mouse scRNA-seq data**

The raw single-cell RNA-seq (scRNA-seq) data underwent initial processing with the Cell Ranger software (v3.1.0) by 10x Genomics, converting the data to the fastq format. Subsequently, the reads were aligned to the mm10 reference genome and quantified using the 'cellranger-arc count' module. The resulting gene-by-cell matrix, reflecting the expression profiles of individual cells, was then imported into the Seurat package for comprehensive downstream analysis (v4.1.3). Cells containing detected genes numbering greater than 200 have been included in the analysis. The expression levels of mitochondrial genes are indicative of a cell's state. Establishing a criterion for mitochondrial gene expression is inherently subjective. Following the clustering process, clusters demonstrating elevated mitochondrial gene expression will be excluded to filter out cells that are potentially in a compromised state. In total, 36,893 cells have been retained for subsequent downstream analyses. The four filtered gene-by-cell matrices were individually utilized as inputs for the CreateSeuratObject function, leading to the creation of distinct Seurat objects. Subsequently, the NormalizeData function was applied to each gene expression matrix, implementing log-normalization for standardized analysis across samples. The identification of the top 5000 most variable genes, indicative of high cell-to-cell variations, was accomplished using the FindVariableFeatures function. The dimensionality of this subset of variable features was then reduced through the application of the RunPCA and RunUMAP functions. In executing the RunPCA function, the parameter 'dims' was configured to encompass dimensions '1:30'. To mitigate batch effects and safeguard the biological variation, the canonical correlation analysis was employed through the FindIntegrationAnchors function for seamless data integration. Cell clustering was performed using the FindNeighbors and FindClusters functions, with a specified cluster resolution of 0.6. Subsequently, 2-dimensional representations were generated employing uniform manifold approximation and projection (UMAP).

Given that the cell types under investigation in this study pertain to cell subtypes, their definition proves challenging with the reliance on only one or two genes. To address this, an extensive collection of genes or pathways previously reported to delineate cell subtypes was compiled. Subsequently, the AddModuleScore function was employed to compute the average expression of each program (feature list) at the single-cell level, facilitating a nuanced characterization of cell subtypes. Detailed gene lists employed for defining these cell subtypes are provided in Supplementary Table 1.

**Procession of mouse scATAC-seq data**

The raw single-cell RNA-seq (scRNA-seq) data underwent conversion to the fastq format employing the Cell Ranger software (10x Genomics, v3.1.0). Subsequently, the reads were aligned to the mm10 reference genome and quantified using the 'cellranger-arc count' module. The resulting processed feature-by-cell matrix was then imported into the Signac package for comprehensive downstream analysis (v1.10.0). Cells with detected fragments ranging from 1000 to 20000 were selectively included in the analysis. The scATAC-seq data from cortex and GE region of embryonic day 17 (E17.5) were merged. To identify the most frequently observed features, the FindTopFeatures function was applied with default parameters. The RunTFIDF function, employing default parameters, was employed for data normalization. Subsequently, singular values were computed based on the normalized counts. Utilizing UMAP, 2-dimensional representations were generated with the 'reduction' parameter set to 'lsi', and dimensions 2 through 30 were utilized. The integration of multiome single-cell RNA-seq (scRNA-seq) data and single-cell ATAC-seq (scATAC-seq) data is facilitated through matching cell barcodes. Subsequently, the scATAC-seq data can be enriched with cell type annotations inferred from the paired scRNA-seq data.

**Construction of the mouse embryonic neurogenic trajectory**

Several cell types—mitochondrial cells, Cajal-Retzius cells, choroid plexus/meninges cells, microglia, and blood cells—were excluded, because they are not directly involved in nervous system development. To construct the developmental trajectory, we converted the Seurat object into a SingleCellExperiment object and then ran the STREAM pipeline (v1.1), which performs variable gene selection, dimensionality reduction, and elastic principal graph construction. his comprehensive approach allows for the delineation of the developmental path, focusing on the pertinent cell types involved in nervous system development.

**Comparison of quiescent state and dividing state**
To scrutinize the differential expression between the quiescent and dividing states, the FindMarkers function from Seurat was employed. The ensuing analysis included genes with a p-value less than 0.05, resulting in the identification of 489 differentially expressed genes (DEGs). Subsequently, an enrichment analysis for quiescent-specific genes was conducted using the ClusterProfile package (v4.6.2). Specifically, Gene Ontology (GO) terms with a false discovery rate (FDR)-adjusted p-value below 0.05 were retained. To forecast potential transcription factors (TFs) with upstream regulatory roles influencing the differential expression of genes (DEGs) and subsequently modulating cell states, the LISA package (v2.3.0) was employed. The top 100 most significant TFs predicted by LISA were selected. Subsequently, the Cistrome DB database was utilized to identify targets of these TFs based on ChIP-seq data. Targets that overlapped with the DEGs were retained for further downstream analysis. Additionally, leveraging single-cell ATAC-seq (scATAC-seq) data, differential expression of peaks (DEPs) between the quiescent and dividing states was calculated using the FindMarkers function from Signac. Differential expression peaks (DEPs) with a p-value less than 0.05 were included, and their nearest associated genes were inferred using the ClosestFeature function in Signac. These genes are referred to as DEPs-gene. The overlapping subset of filtered DEGs and DEPs-gene was subjected to network analysis. The transcription factors (TFs) inferred to regulate these overlapping genes were then defined.

**Quantification and statistical analysis**

Statistical analyses were performed with Student’s t test or one-way ANOVA followed by Dunnett’s posttest using Prism (GraphPad Software). Data are presented as mean ± SEM p < 0.05 was considered significant. The value of n for each graph was stated in Figure legend.

**Reference**

1.	Madisen, L., et al., *A robust and high-throughput Cre reporting and characterization system for the whole mouse brain.* Nat Neurosci, 2010. **13**(1): p. 133-40.

2.	Tronche, F., et al., *Disruption of the glucocorticoid receptor gene in the nervous system results in reduced anxiety.* Nat Genet, 1999. **23**(1): p. 99-103.

3.	Gorski, J.A., et al., *Cortical excitatory neurons and glia, but not GABAergic neurons, are produced in the Emx1-expressing lineage.* J Neurosci, 2002. **22**(15): p. 6309-14.

4.	Hao, Y., et al., *Integrated analysis of multimodal single-cell data.* Cell, 2021. **184**(13): p. 3573-3587.e29.

5.	Wang, C., et al., *Integrative analyses of single-cell transcriptome and regulome using MAESTRO.* Genome Biol, 2020. **21**(1): p. 198.

6.	Yu, G., et al., *clusterProfiler: an R package for comparing biological themes among gene clusters.* Omics, 2012. **16**(5): p. 284-7.

7.	Stuart, T., et al., *Single-cell chromatin state analysis with Signac.* Nat Methods, 2021. **18**(11): p. 1333-1341.

8.	Etherington, G.J., R.H. Ramirez-Gonzalez, and D. MacLean, *bio-samtools 2: a package for analysis and visualization of sequence and alignment data with SAMtools in Ruby.* Bioinformatics, 2015. **31**(15): p. 2565-7.

9.	Chen, H., et al., *Single-cell trajectories reconstruction, exploration and mapping of omics data with STREAM.* Nat Commun, 2019. **10**(1): p. 1903.

10.	Stanstrup, J., et al., *The metaRbolomics Toolbox in Bioconductor and beyond.* Metabolites, 2019. **9**(10).

11.	Qin, Q., et al., *Lisa: inferring transcriptional regulators through integrative modeling of public chromatin accessibility and ChIP-seq data.* Genome Biol, 2020. **21**(1): p. 32.

12.	Zheng, R., et al., *Cistrome Data Browser: expanded datasets and new tools for gene regulatory analysis.* Nucleic Acids Res, 2019. **47**(D1): p. D729-d735.

13.	Mirzadeh, Z., et al., *The subventricular zone en-face: wholemount staining and ependymal flow.* J Vis Exp, 2010(39).

14.	Hu, X.L., et al., *Persistent Expression of VCAM1 in Radial Glial Cells Is Required for the Embryonic Origin of Postnatal Neural Stem Cells.* Neuron, 2017. **95**(2): p. 309-325 e6.

15.	Gao, P., et al., *Deterministic progenitor behavior and unitary production of neurons in the neocortex.* Cell, 2014. **159**(4): p. 775-88.
